# Versatile CRISPR‐Cas Tools for Gene Regulation in Zebrafish via an Enhanced Q Binary System

**DOI:** 10.1002/advs.202511485

**Published:** 2026-02-11

**Authors:** Miaoyuan Shi, Weiqi Ge, Changheng Li, Bin Liu, Xiaoyi Deng, Chengjie Liu, Meijun Zheng, Pu Zhang, Lei Li, Ying Guo, Yunqi Han, Yu Yang, Yanxun V. Yu, Youngnam N. Jin

**Affiliations:** ^1^ Department of Nuclear Medicine, Medical Research Institute, Zhongnan Hospital of Wuhan University Wuhan University Wuhan China; ^2^ State Key Laboratory of Biocatalysis and Enzyme Engineeringhubei Hongshan Laboratory, School of Life Sciences Hubei University Wuhan China; ^3^ Department of Neurology, Medical Research Institute, Frontier Science Center for Immunology and Metabolism, Zhongnan Hospital of Wuhan University Wuhan University Wuhan China; ^4^ Department of Nuclear Medicine, Medical Research Institute, Frontier Science Center for Immunology and Metabolism, Zhongnan Hospital of Wuhan University Wuhan University Wuhan China

**Keywords:** amyotrophic lateral sclerosis, Cas13, CRISPRa, CRISPR‐Cas, QF/QUAS, transgenics, zebrafish

## Abstract

CRISPR‐Cas systems revolutionize gene regulation across diverse organisms, including zebrafish. However, most zebrafish studies still rely on transient delivery of CRISPR components, with limited use of transgenic models, primarily restricted to Cas9‐mediated knockouts. This limitation arises from challenges in achieving sustained, tissue‐specific, and efficient expression of transgenic CRISPR effectors. To address these challenges, we introduce CRISPR‐Q, a transgenic system that combines the QFvpr/QUAS binary expression platform with CRISPR‐Cas technologies. CRISPR‐Q overcomes the drawbacks of transient mRNA or protein delivery and circumvents the toxicity and transgene silencing issues associated with other binary systems, such as Gal4/UAS. The system enables robust and spatiotemporal expression of CasRx or dCas9vpr, allowing precise transcript knockdown (CRISPR‐Q_KD_) or gene activation (CRISPR‐Qa). Using CRISPR‐Q_KD_, we achieve effective knockdown of *smn1* and simultaneous knockdown of *tardbp* and *tardbpl*, modeling spinal muscular atrophy and amyotrophic lateral sclerosis, respectively. CRISPR‐Qa activates endogenous *lin28a* and *sox9b*, demonstrating its functional versatility. We further validate CRISPR‐Q's tissue‐specific applicability in heart‐specific transgenic zebrafish. Together, CRISPR‐Q represents a robust and versatile platform for studying gene function and modeling human diseases in zebrafish, with broad potential for adaptation in other model organisms.

## Graphical abstract

This study introduces CRISPR‐Q, a transgenic CRISPR‐Cas system leveraging the QFvpr/QUAS binary expression platform in zebrafish. CRISPR‐Q overcomes previous challenges in achieving stable and efficient gene regulation. By enabling precise spatiotemporal control of transcript knockdown (CRISPR‐Q_KD_) and gene activation (CRISPR‐Qa), it provides a powerful and versatile tool for gene function studies and disease modeling in transgenic zebrafish.

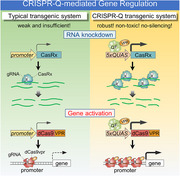

## 1 Introduction

Controlling gene expression is essential for comprehending the function of a target gene. The CRISPR‐Cas9 system has been widely used in zebrafish for various genetic modifications, including targeted gene knockout (KO) [1], knockin [2], multiple gene KOs [3], biallelic gene disruption [3, 4], and reverse genetic screening [4, 5, 6, 7]. Beyond inducing indel mutations, Cas9 can also be utilized to control gene expression. By combining dCas9 (catalytically inactive Cas9) with a transcriptional activator or repressor, it can be transformed into either an activator (CRISPRa) or CRISPR interference (CRISPRi) [8, 9, 10]. In zebrafish, introducing dCas9‐VP160 mRNA for CRISPRa (or referred to as CRISPR‐on) or dCas9‐KRAB (or dCas9‐Eve) mRNA as CRISPRi into zebrafish embryos led to the upregulation or reduction of target gene expression, respectively [11, 12].

In the context of transcript knockdown in zebrafish, morpholinos (MOs) have been in widespread use for over two decades. Lately, however, concerns about the effectiveness of MOs have arisen due to documented instances of toxicity and off‐target effects. While CRISPRi can potentially suppress transcriptional activity, the initial stages of embryogenesis heavily rely on maternally inherited mRNAs, not controllable by CRISPRi, prior to zygotic gene activation. Cas13, classified as a class 2 type VI CRISPR‐Cas RNA endonuclease, has recently been employed to induce the degradation of RNA in various organisms, including yeast, plants, and mammalian cell lines [13, 14, 15, 16, 17]. In zebrafish embryos, RfxCas13d (also known as CasRx), Cas13d from *Ruminococcus flavefaciens*, efficiently and precisely downregulated specific mRNA transcripts expressed both zygotically and maternally, without apparent toxicity, a distinction from PspCas13b, PguCas13b, or LwaCas13a [18].

Despite these advances, the majority of zebrafish experiments have depended primarily on the injection of either mRNA or purified Cas effector proteins, along with one or multiple guide RNAs (gRNAs), into embryos. However, understanding the molecular mechanisms of a target gene or modeling human diseases often requires the use of transgenic animals that allow for tissue‐specific or temporal control of gene expression. Although transgenic Cas9 zebrafish have been used for gene inactivation by KO [19, 20, 21, 22], there have been no documented cases of transgenic CRISPR zebrafish that facilitate controlled gene activation, downregulation, or transcript knockdown. Unlike Cas9‐driven gene knockout, achieving transcript knockdown with CasRx (or other Cas13 effectors), or up‐ or downregulation of a gene through CRISPRa or CRISPRi, requires sustained high expression of CRISPR effector proteins. While the injection of mRNA or protein of Cas effectors can transiently introduce high level expression, transgenic expression of these effectors often falls short in inducing the desired effect. Similarly, in previous endeavors, we dedicated substantial effort to creating transgenic zebrafish that express Cas effectors for regulating gene expression, but we mostly encountered challenges in achieving noticeable changes. We postulated that elevating the expression level of the Cas effector would enhance the efficiency of gene regulation. With this in mind, we opted to integrate the binary expression system with the Cas effector in zebrafish.

Binary expression systems offer a powerful approach to enhance gene expression with flexibility for tissue‐specific or temporal control. They consist of a transcriptional activator along with multiple repeats of the corresponding DNA‐binding element. The Gal4/UAS system has been effectively employed as a primary binary system in *Drosophila*. However, its utility in transgenic zebrafish is constrained by two limitations: UAS silencing and Gal4 toxicity [23, 24]. In vertebrates, the accumulation of methylations on CpG dinucleotides within the UAS element can lead to its silencing, posing challenges in the use of these systems in zebrafish transgenesis. Although modified versions of UAS have shown reduced variegation and gene expression silencing, transcriptional silencing in the UAS promoter persists across multiple generations [23]. In order to circumvent the methylation issue in the GAL4/UAS‐based system, a CpG‐free element (tUAS) was introduced in zebrafish along with the tryptophan repressor (TrpR). However, the excessive toxicity of this driver still restricted its use [25]. The Gal4 transactivator itself is toxic in zebrafish, particularly when expressed ubiquitously [23]. While Gal4FF, which includes a modified VP16 [23], and KalTA4, which incorporates a potent Kozak sequence, codon‐optimized Gal4 DNA binding domain, and a modified activation domain referred to as TA4 [26], reportedly reduce the toxicity, transgenerational silencing remains a drawback in these Gal4/UAS systems. Thus, a demand exists for a binary expression system in zebrafish that is both non‐toxic and remains unaffected by DNA methylation‐induced silencing over successive generations. The Q binary expression system, originating from the fungus *Neurospora crassa*, is advantageous due to the absence of CpG dinucleotides in the consensus binding site for the QF transactivator, effectively preventing silencing through CpG‐mediated methylation. The Q binary system has been successfully employed to regulate gene expression in *Drosophila* [27, 28], cultured mammalian cells [28], *C. elegans* [29], and zebrafish [30].

In this study, we present a method for precise and robust control over transcript knockdown and gene activation in transgenic zebrafish. This accomplishment is made possible by integrating CRISPR‐Cas effector expression with an enhanced QFvpr (a fusion of QF DNA binding domain with three transcriptional activators: VP64, p65, and Rta)/QUAS binary expression system, which we have termed the CRISPR‐Q system. With the CRISPR‐Q system, we successfully achieved robust expression of CRISPR effector proteins, such as CasRx and dCas9vpr, without toxicity or gene silencing. This allows for sustained transcript knockdown and gene activation by CasRx and dCas9vpr, respectively. Using this robust expression, we demonstrate that transcript knockdown by CRISPR‐Q coupled with CasRx, termed CRISPR‐Q_KD_, can efficiently knock down not only a single gene, *smn1*, but also two paralogous genes, *tardbp* and *tardbpl*, replicating disease phenotypes of spinal muscular atrophy (SMA) and amyotrophic lateral sclerosis (ALS), respectively. Furthermore, CRISPR‐Q coupled with dCas9vpr, termed CRISPR‐Qa, was able to activate two endogenous genes, *lin28a* and *sox9b*, in transgenic zebrafish. Additionally, we demonstrated the heart‐specific applicability of the CRISPR‐Q system in transgenic zebrafish. In conclusion, our study demonstrates the promising potential of the CRISPR‐Q system to overcome current limitations in regulating gene expression using transgenic animals.

## 2 Results

### 2.1 QFvpr/QUAS System Is a Superior Binary System

**FIGURE 1:**
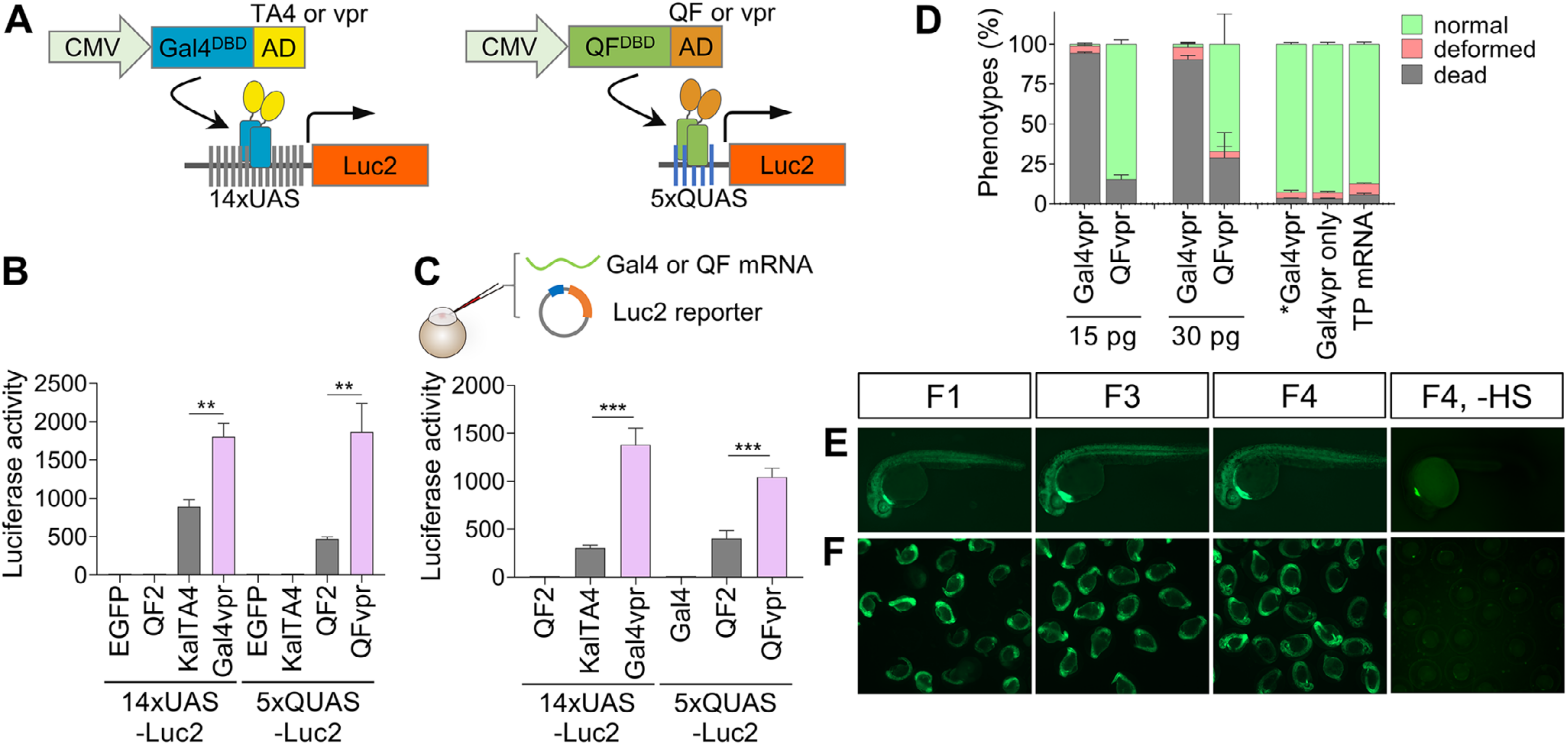
QFvpr/QUAS outperforms other binary systems. (A) Schematic illustration of the Gal4/UAS (left) and QF/QUAS (right) binary systems, both driven by the CMV (cytomegalovirus) promoter, each paired with its corresponding luciferase reporter. DBD, DNA‐binding domain. (B) In vitro luciferase activity test for various binary transactivation systems. HEK293T cells were co‐transfected with the indicated transcription activator and a corresponding luciferase reporter plasmid. (C). In vivo luciferase activity test for various binary transactivation systems. Zebrafish embryos at the one‐cell stage were co‐injected with transactivator mRNA and a designated reporter plasmid, with luciferase activity evaluated at 6–7 h post‐fertilization (hpf). (B, C) Relative luciferase activity was quantified using pooled lysates from 20–30 embryos per condition, as described in the methods. *n* = 3. Data are presented as mean ± s.e.m. *P* values are derived from an unpaired two‐tailed *t‐*test. ^**^ *p* < 0.01; ^***^ *p* < 0.001. (D) Toxicity comparison between Gal4vpr and QFvpr. A specified amount of Gal4vpr or QFvpr plasmid, expressed under the *ubb* (ubiquitin B) promoter, was co‐injected with 25 pg of Tol2 transposase (TP) mRNA into zebrafish embryos. The toxicity levels for each condition were evaluated by visual inspection at 5 days post‐fertilization (dpf) ^*^Gal4vpr (30 pg injection) refers to a frame shift mutant of Gal4vpr that results in premature translation termination. Data were obtained from three independent experiments, with 68–130 embryos analyzed per condition. Data are shown as mean ± s.e.m. (E, F) The Q system remains resistant to gene silencing across successive generations. The double transgenic, 2Tg, zebrafish line was generated by crossing Tg(*hsp70l*:QFvpr‐CK) with Tg(5*×QUAS*:CasRx‐2A‐EGFP‐CG), yielding F1 2Tg zebrafish. F3 or F4 generations were produced by mating F2 or F3 adult zebrafish, respectively. Embryos at 24 hpf were subjected to heat treatment at 37°C for 1 h. Fluorescence images of EGFP were captured for individual dechorionated embryos (E) or groups of embryos within their chorions (F) at 32 hpf. A heat shock‐free (‐ HS) group derived from F4 embryos was included as a negative control. CK, *cmlc2*:mKate2, and CG, *cmlc2*:EGFP, were used as heart markers.

In an effort to develop a more robust binary transgenic system in zebrafish, we tested whether increasing the number of VP16 repeats could enhance transcription activation. To this end, we generated a series of Gal4 constructs with varying numbers of the minimal VP16 transactivation domain (AD) [31], hereafter referred to as VP16, from VP64 (4×VP16) to VP224 (14×VP16). We compared their transactivation activity with that of KalTA4, an improved version of Gal4 for zebrafish [26], which consists of a strong Kozak sequence, a codon‐optimized Gal4 DNA binding domain, and a modified AD termed TA4, containing one copy of VP16 and two VP16 variants. KalTA4 was shown to be similarly potent as, but less toxic than, Gal4, especially in zebrafish [26]. Unexpectedly, we did not observe any significant increase in transcription activation by Gal4 with an increasing number of VP16 repeats, except for VP64, which was modestly more active (Figure S1). We next tested the effect of enhancing the nuclear localization of Gal4 on its activity. However, we still did not find any noticeable change in transcription activity (Figure S1). Given these results, we selected VPR, which is a fusion of three transcriptional activators, VP64, p65, and Rta (VP64‐p65‐Rta), as an AD because it includes VP64 and has been shown to be one of the most effective and reliable transactivators across a wide range of organisms [32, 33, 34, 35]. As expected, in vitro experiment showed that Gal4vpr significantly increased the transcription of luciferase reporter compared to KalTA4 (Figure 1A,B).

Similarly, building on the increased activation of Gal4 by VPR, we constructed the QFvpr transactivator by combining the QF DNA binding domain (QF DBD) with VPR as an AD to test whether VPR could enhance QF‐mediated transactivation. The original QF transactivator was quite toxic when expressed broadly [28]. The subsequent study discovered a non‐toxic variant, QF2, which lacks a toxic middle domain but retains its activation domain [27]. Notably, QFvpr showed much greater reporter gene activation than QF2 and comparable activity to Gal4vpr (Figure 1A,B). Next, we compared the activities of Gal4vpr and QFvpr in zebrafish embryos. We injected transactivator mRNA along with the indicated reporter plasmids into zebrafish embryos and obtained similar results, showing that VPR drove substantially higher activation than TA4 in KalTA4 or QF‐AD in QF2 (Figure 1C).

Having confirmed that QFvpr displays similar transactivation to Gal4vpr in zebrafish, we next examined the relative toxicity of QFvpr and Gal4vpr in zebrafish embryos. Like the original QF transactivator, Gal4 is also known to exhibit toxicity, especially when expressed pan‐neuronally or ubiquitously [23, 24]. To assess toxicity, we injected embryos with a plasmid containing either QFvpr or Gal4vpr under the control of the zebrafish *ubb* (ubiquitin B) promoter, which drives constitutive and ubiquitous expression, together with Tol2 transposase (TP) mRNA (Figure 1D). Embryos injected with Gal4vpr could not survive even at the low concentration, such as 15 pg. In stark contrast, most of the embryos were able to tolerate QFvpr much better than Gal4vpr. Notably, no toxicity was observed in embryos injected with a frameshift mutant of Gal4vpr (^*^Gal4vpr), the Gal4vpr plasmid alone (without TP mRNA), or TP mRNA alone, suggesting that the observed toxicity is specifically due to the expression of the Gal4vpr protein. Given that Gal4vpr and QFvpr share the same AD (VPR) but differ in their DBDs (Gal4 or QF), these results indicate that the Gal4 DBD is inherently more toxic than the QF DBD. We raised the surviving embryos following injection with *ubb*:QFvpr‐CK or *ubb*:Gal4vpr‐CK. We successfully identified founder zebrafish for *ubb*:QFvpr‐CK but not for *ubb*:Gal4vpr‐CK, despite multiple injections and rigorous screening. Additionally, we had previously attempted to generate transgenic zebrafish carrying *ubb*:KalTA4. Although KalTA4 is believed to be less toxic than Gal4, we failed to establish any founder lines (Figure S2A), suggesting that KalTA4 is still too toxic when expressed ubiquitously. Collectively, these results indicate that QFvpr exhibits substantially less toxicity compared to Gal4vpr as well as KalTA4.

To test whether the QFvpr/QUAS system could drive the expression of CRISPR effector proteins, double transgenic (2Tg) zebrafish were generated by crossing the *ubb*:QFvpr‐CK and 5×*QUAS*:CasRx‐2A‐EGFP‐CG lines. The expression of CasRx (a.k.a. RfxCas13d) containing two nuclear localization signal (NLS) sequences was driven by 5×*QUAS* upon binding of QFvpr. To knock down the target gene *smn1*, the 2Tg zebrafish was crossed with another transgenic line expressing CasRx guide RNA (gRNA) targeting *smn1*, generating triple transgenic (3Tg) zebrafish, Tg(*ubb*:QFvpr‐CK; 5×*QUAS*:CasRx‐2A‐EGFP‐CG; zU6:*smn1‐*AV) (Figure S2B). However, we found that CasRx expression appeared too weak, as indicated by the fluorescence of EGFP via the 2A ribosome‐skipping sequence, and no noticeable knockdown of *smn1* transcripts was observed (Figure S2C). We reasoned that the *ubb*:QFvpr‐CK founder line may carry only a single copy, as higher copy numbers could cause excessive QFvpr expression and toxicity, similar to that observed with high doses in the acute injection experiment (Figure 1D). This notion is further supported by the difficulty in establishing a *ubb*:QFvpr‐CK founder line (Figure S2A). Therefore, we conclude that ubiquitous QFvpr expression may still exert a certain degree of toxicity.

In addition to *ubb* transgenic zebrafish lines, we generated the transgenic zebrafish line Tg(*hsp70l*:QFvpr‐CK), whose expression is driven by heat shock, allowing for temporal control of transactivation. Upon heat treatment of embryos with 2Tg transgenic expression of *hsp70l*:QFvpr‐CK and 5×*QUAS*:CasRx‐2A‐EGFP‐CG, we observed bright expression of EGFP, suggesting robust transcriptional activation of CasRx (Figure 1E,F). As a result, we chose to focus on transgenic zebrafish lines driven by the heat shock promoter, *hsp70l*, for subsequent studies.

Given that a significant drawback of the Gal4/UAS system is gene silencing due to methylation of the UAS promoter, we tested whether the QFvpr/QUAS system could sustain transactivation over generations. Unlike Gal4/UAS, we did not find any gene silencing in the QFvpr/QUAS binary system over the F4 generation as of now (Figure 1E,F), consistent with previous reports [30, 36]. Interestingly, a recent study showed that 5×*QUAS* elements are heavily methylated on CpG dinucleotides, but the QF DBD still binds to this cis‐regulatory element regardless of methylation status [37], supporting our findings of no discernible transgene silencing.

### 2.2 Experimental Design for CRISPR‐mediated Gene Regulation in Transgenic Zebrafish Using a Binary Expression System

**FIGURE 2:**
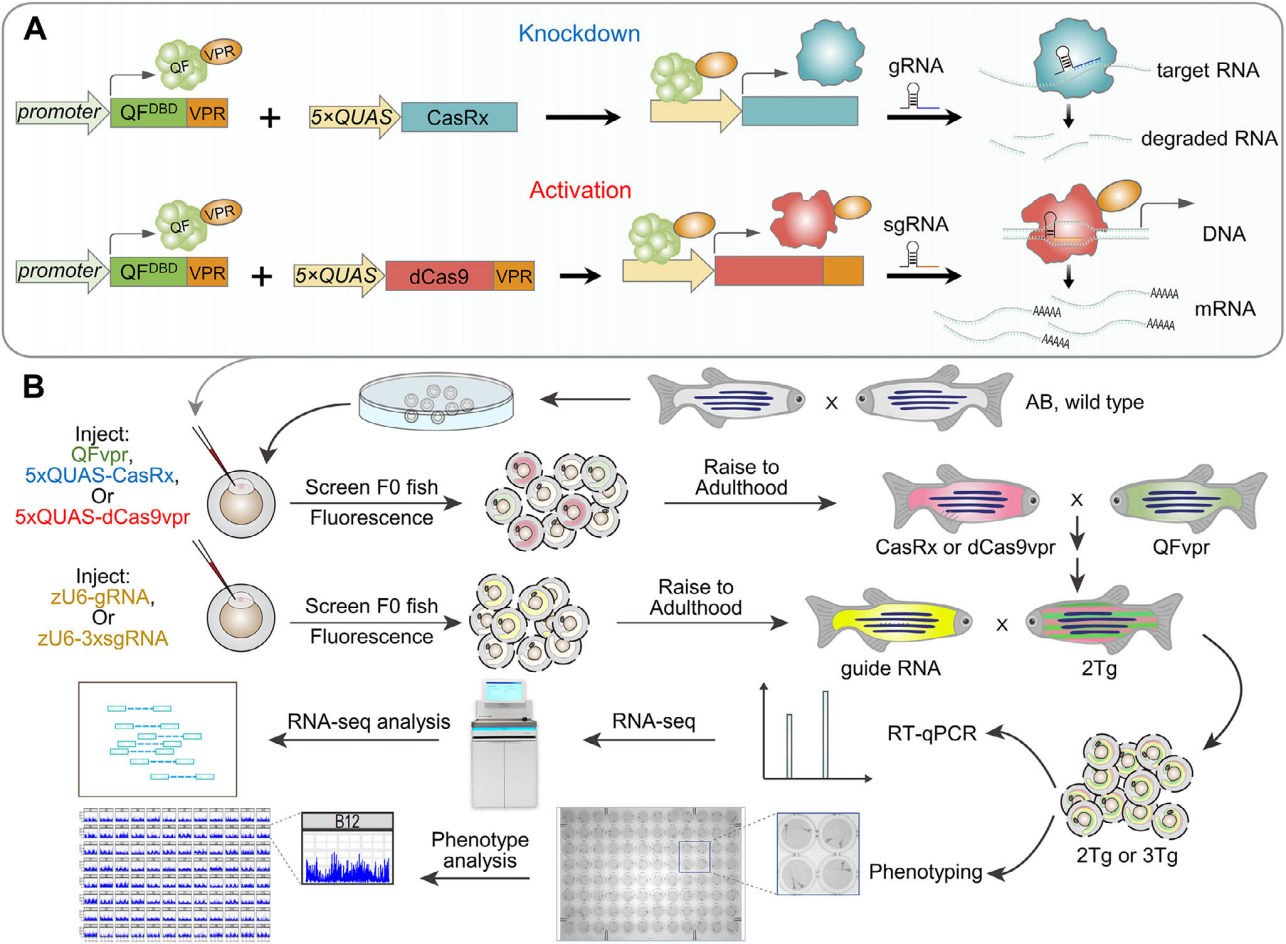
Schematic of the CRISPR‐Q system: an enhanced CRISPR‐Cas tool via the Q binary system for generating transgenic zebrafish with RNA knockdown or gene regulation. (A) Schematic diagram of knockdown or gene activation by the CRISPR‐Q system, which consists of three components: QFvpr under the control of a specific promoter, 5*×QUAS‐*mediated CRISPR effectors (CasRx for knockdown and dCas9vpr for gene activation), and guide RNA (gRNA or sgRNA). QF DBD, QF DNA binding domain. (B) Plasmid constructs, including QFvpr regulated by a specific promoter for spatiotemporal control, CasRx or dCas9vpr under the control of a 5*×QUAS* promoter, or guide RNA driven by zebrafish U6 (zU6) promoter, were injected into one‐cell embryos. The injected embryos were raised to adulthood as F0 transgenic zebrafish and then screened for germline transmission. Following a series of outcrossings to obtain double or triple transgenic embryos, 2Tg or 3Tg, respectively, these embryos underwent heat shock treatment. Subsequently, the embryos or larvae were analyzed for transcriptional levels or subjected to phenotypic behavioral assays.

To achieve robust and reliable expression of CRISPR effector proteins, we coupled the QFvpr/QUAS binary system with CRISPR effectors driven by a 5×*QUAS* promoter (Figure 2), thereby generating the CRISPR‐Q system. For upregulating endogenous gene expression, dCas9vpr, reported as one of the most robust activators [32], was selected as the effector for transcription activation. For transcript knockdown by Cas13 proteins, we first compared the knockdown efficiencies of two Cas13 proteins, CasRx and Cas13X (a.k.a. Cas13bt3), as both have been reported to exhibit minimal collateral activity, unlike other Cas13 proteins such as Cas13a and Cas13b [13, 38]. Overall, CasRx displayed more robust knockdown activity than Cas13X (Figure S3), although a previous study reported comparable activities between the two proteins [39]. Therefore, CasRx was chosen to generate transgenic zebrafish for transcript knockdown.

The expression of the CRISPR effectors is driven by the 5×*QUAS* promoter upon binding of the QF transactivator in 2Tg zebrafish carrying a promoter‐specific QFvpr and 5×*QUAS*:Cas effector. 3Tg zebrafish were generated by crossing 2Tg fish with another transgenic line expressing gRNAs specific for either CasRx or dCas9, together with AV (α‐*crystallin*:Venus) as a transgenic marker. Venus is a derivative of YFP. The 2Tg embryos were identified by CK (*cmlc2*:mKate2), CG (*cmlc2*:EGFP), or AK (α‐*crystallin*: mKate2) markers, indicating red heart, green heart, or red lens, respectively. The 3Tg embryos were distinguished by the additional AV marker, which produces a green lens. These 3Tg embryos or larvae were then used to assess RNA expression levels and evaluate behavioral responses to various stimuli in comparison with 2Tg controls.

### 2.3 CRISPR‐Q System Enables Effective mRNA Knockdown via CasRx in Transgenic Zebrafish

**FIGURE 3:**
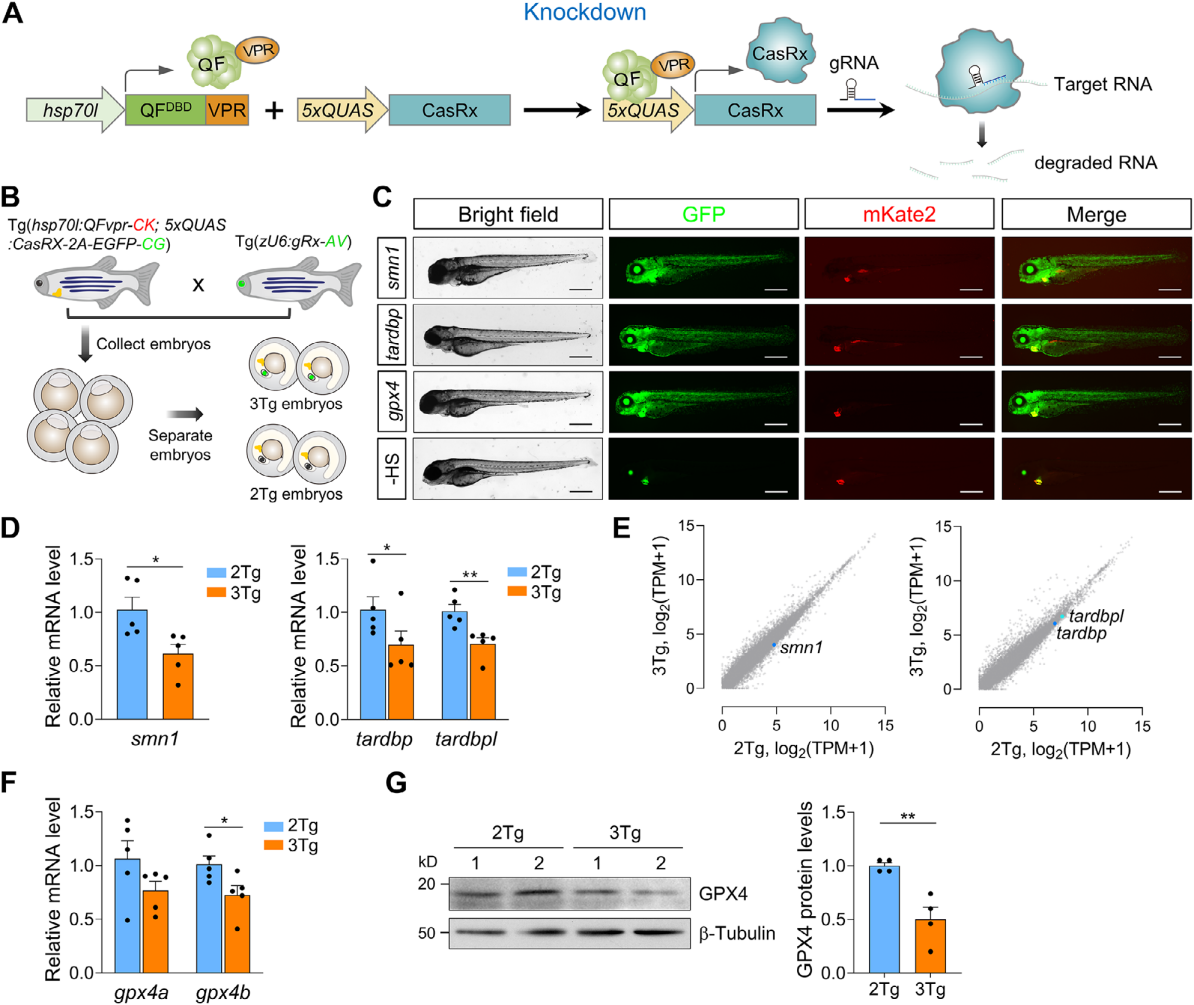
QFvpr binary system facilitates sufficient knockdown of endogenous genes by CasRx in vivo. (A) Diagram illustrating the application of the CRISPR‐Q system for transcript knockdown. (B) Experimental procedure: The 2Tg zebrafish, Tg(*hsp70l*:QFvpr‐CK; 5×*QUAS*:CasRx‐2A‐EGFP‐CG) were outcrossed with transgenic zebrafish expressing gRNA targeting *smn1*, *tardbp* covering both *tardbp* and *tardbpl*, or *gpx4* covering both *gpx4a* and *gpx4b*. The resulting offspring embryos were divided into 3Tg or 2Tg groups for subsequent experiments. CK, *cmlc2*:mKate2; CG, *cmlc2*:EGFP; AV, α‐*crystallin*:Venus. (C) Representative images of 3Tg embryos following heat shock treatment. Embryos underwent two heat shock treatments at 37°C for 1 h on 2 dpf, followed by two additional treatments at 38°C for 30 min on 3 dpf, with an 8‐h interval between the two heat treatments. Larvae were collected for analysis at 4 dpf. A heat shock‐free (‐ HS) larva derived from 3Tg *smn1* line was included as a negative control. Scale bar, 500 µm. (D) The qPCR assays were conducted for *smn1* (left) and *tardbp* (right). *n* = 5. (E) Scatter plots derived from RNA‐seq analysis revealed minimal global transcriptome changes in transcript abundance between 2Tg and 3Tg samples, with a slight but noticeable reduction in *smn1* (left) and *tardbp* (right). Three independent biological replicates were performed for each condition. The blue dots highlight the *smn1* or *tardbp* gene, and the light blue dot indicates the *tardbpl* gene. (F) The qPCR assays were conducted for *gpx4*. *n* = 5. (G) Western blot analysis of GPX4 protein levels in 4‐day‐old 2Tg and 3Tg *gpx4* zebrafish larvae. A representative blot from two independent biological repeats is shown. The plot on the right shows quantitation of the Western blot data. *n* = 4. Data are presented as mean ± s.e.m. Each replicate was performed with 20–30 pooled larvae (D–G). *P* values are derived from an unpaired two‐tailed *t‐*test. ^*^ *p* < 0.05; ^**^ *p* < 0.01.

To test whether the CRISPR‐Q system, coupled with CasRx, termed CRISPR‐Q_KD_, can effectively knock down transcripts in zebrafish, 2Tg zebrafish carrying *hsp70l*:QFvpr‐CK and 5×*QUAS*:CasRx‐2A‐EGFP‐CG were crossed with transgenic zebrafish expressing different gRNAs, producing 3Tg zebrafish embryos (Figure 3A,B). The resulting embryos were subjected to daily transient heat treatment from 2 days post‐fertilization (dpf). Notably, the following day we observed strong transactivation of CasRx, as assessed by EGFP fluorescence (Figure S4A). Most of the embryos developed normally at 2 dpf, and the heat treatment could be repeated in the following days for a longer duration of gene regulation (Figure 3C; Figure S4B).

Next, we tested the knockdown efficiency mediated by CRISPR‐Q_KD_. To this end, 3Tg zebrafish embryos expressing QFvpr, 5×*QUAS*:CasRx, and a gRNA targeting *smn1* or *urod* underwent heat treatment. Upon induction of CasRx, *smn1* expression was significantly decreased (0.61±0.088) (Figure 3D), whereas *urod* expression showed only a trend toward reduction (Figure S5A). Insufficient gRNA target site activity for *urod* is likely the main reason for the limited knockdown, as *urod* is not duplicated, and *smn1* was efficiently knocked down under comparable CasRx expression levels. The zebrafish genome often harbors duplicated genes, which can hinder accurate phenotypic analysis by a single KO or may require time‐consuming and laborious work to establish double homozygous KO mutant lines [40, 41, 42]. Given that CasRx has the ability to process pre‐CRISPR RNA (pre‐crRNA), we tested whether CRISPR‐Q_KD_ could simultaneously knock down two mRNA targets from duplicated genes. We generated 3Tg zebrafish lines expressing an array of two gRNAs targeting two paralogs of *tardbp* (*tardbp* and *tardbpl*, or termed *tardbpb* and *tardbpa*, respectively), or *gpx4* (*gpx4a* and *gpx4b*). The 3Tg zebrafish larvae showed significantly reduced levels of both *tardbp* (0.70±0.129) and *tardbpl* (0.71±0.058) (Figure 3D). Similarly, we observed a substantial reduction in the expression levels of *gpx4a* (0.77±0.084) and *gpx4b* (0.73±0.088) (Figure 3D,F). RNA‐seq analysis showed that CRISPR‐Q_KD_ exhibits modest reductions in the expression of *smn1* and *tardbp* paralogs (*tardbp* and *tardbpl*) with limited off‐target knockdown (Figure 3E), consistent with previous studies [15, 18]. Furthermore, we also performed Western blot analysis to assess GPX4 protein levels and observed a remarkable 50% reduction in 3Tg *gpx4* larvae (Figure 3G). It is noteworthy that all gRNAs were designed to minimize off‐target effects and have no potential off‐target sites with fewer than five mismatches. Taken together, the CRISPR‐Q system coupled with CasRx, namely CRISPR‐Q_KD_, achieved effective transcript knockdown in transgenic zebrafish.

Collateral activity is a critical concern when using Cas13 proteins. Although CasRx has been reported to exhibit lower toxicity [18], it can still display collateral activity, particularly when targeting highly abundant endogenous or ectopic RNAs [43, 44]. To assess CasRx collateral activity in zebrafish, we co‐injected CasRx mRNA and gRNAs targeting mGreenLantern (mGL) along with mRNAs for mGL and mKate2. We observed that mKate2 fluorescence decreased when the injection amount of mGL mRNA exceeded 50 pg (Figure S6A–C), indicating CasRx‐mediated collateral activity. Consistently, developmental toxicity attributable to collateral activity increased with higher amounts of mGL mRNA (Figure S6D). RNA integrity has been reported to be sensitive to collateral activity [43]. Accordingly, we performed RNA integrity number (RIN) analysis on RNA samples isolated from injected embryos at 6 h post‐fertilization (hpf). We observed pronounced rRNA fragmentation and a marked reduction in RIN in mGL knockdown embryos injected with an excess amount of mGL mRNA (Figure S6E,F). In addition, we examined phosphorylation of p38, a known marker of collateral activity [44], and detected elevated phospho‐p38 levels in mGL knockdown embryos at 6 hpf (Figure S6G). These results indicate that CasRx can exhibit collateral activity when targeting excessive ectopic RNAs in zebrafish. Next, we tested whether CRISPR‐Q_KD_ induces collateral effects in transgenic fish. We injected mKate2 mRNA to 2Tg and 3Tg embryos as a reporter for collateral activity and applied a 45‐min heat shock at 12 hpf to induce CasRx expression. We observed no reduction in mKate2 fluorescence at 30 hpf, no developmental toxicity at 24 hpf (Figure S7A–D), and intact rRNA at 30 hpf (Figure S7E,F). Additionally, phospho‐p38 levels were unchanged (Figure S7G,H), and RNA integrity remained unaffected (Figure S7I,J) in both *smn1* 2Tg and 3Tg larvae at 4 dpf. Together, these results demonstrate that the transgenic CRISPR‐Q_KD_ system does not induce detectable collateral activity or toxicity.

Knockdown efficiency can be influenced by the expression levels of both the Cas effector and gRNA. To determine the required levels of CasRx mRNA (mCasRx) and gRNA for effective knockdown, we compared their expression in 4‐day‐old transgenic larvae with embryos at 24 hpf following injection. We found that CasRx mRNA levels induced by the QFvpr/QUAS system were comparable to those in 24‐hpf embryos injected with 300 pg mCasRx, although mCasRx levels likely decrease substantially after injection (Figure S8A). This result further confirms the ability of CRISPR‐Q_KD_ to drive robust CasRx expression, although CasRx levels in CRISPR‐Q_KD_ transgenic embryos do not reach the high levels observed during the early hours following injection. Interestingly, gRNA injection ranging from 1 pg to 100 pg induced comparable knockdown, with only marginal improvement at higher amounts, suggesting that 10 pg of gRNA may be sufficient (Figure S8B,D). Consistently, gRNA levels in *smn1* 3Tg larvae were higher than those in embryos injected with 10 pg gRNA (Figure S8C), while gRNA levels in *tardbp* 3Tg larvae were comparable to 10 pg injected embryos (Figure S8E). Additionally, injection of mCasRx into gRNA transgenic embryos resulted in significant knockdown comparable to that observed in WT embryos co‐injected with gRNA at 24 hpf (Figure S8K,L). Notably, the knockdown efficiency achieved by these injection experiments at 24 hpf was comparable to that observed in CRISPR‐Q_KD_ transgenic larvae. Collectively, these results suggest that while CasRx expression levels and gRNA abundance significantly contribute to overall efficacy, target site selection plays an important role in knockdown efficiency.

The copy number of transgenes can affect their expression levels, and Tol2‐mediated transgenesis typically yields from a few to over ten copies per line [45, 46, 47]. To investigate the relationship between transgene expression and copy number, we quantified the copy numbers of each component of the CRISPR‐Q_KD_ system in 2Tg and 3Tg lines targeting *smn1* and *tardbp*. Consistent with previous studies, the copy numbers across transgenic lines and among embryos from the same batch ranged from 1–2 copies for QFvpr, 1–3 copies for CasRx, and 3–12 copies for gRNAs, with the *smn1* transgenic line showing higher gRNA copy numbers (Figure S8G,H). These findings demonstrate that the CRISPR‐Q system can be efficiently established using the Tol2 system. Moreover, the copy number analysis was consistent with the expression profiles of CasRx mRNA and gRNA. The copy number and expression levels of CasRx were comparable between the *smn1* and *tardbp* transgenic lines, whereas the *smn1* line exhibited higher gRNA levels. These results suggest that generating transgenic lines with higher copy numbers may further enhance the efficiency of the CRISPR‐Q system.

### 2.4 CRISPR‐Q_KD_‐Mediated Knockdown of *smn1* Leads to SMA‐Related Phenotypes in Zebrafish

Loss of survival motor neuron (SMN) protein, due to mutations in the SMN1 gene, causes functional defects and even death in motor neurons, leading to SMA, in which patients suffer from muscle weakness and wasting, and eventually die [48]. The levels of SMN protein determine the severity and age of onset of SMA in humans, which range from types 1 to 4 [49]. Similarly, loss of *smn1* in zebrafish results in deficits in motor neurons and muscles, reduced motor activity, and early lethality at 4–5 dpf in cases of maternal and zygotic deficiency [50, 51].

**FIGURE 4:**
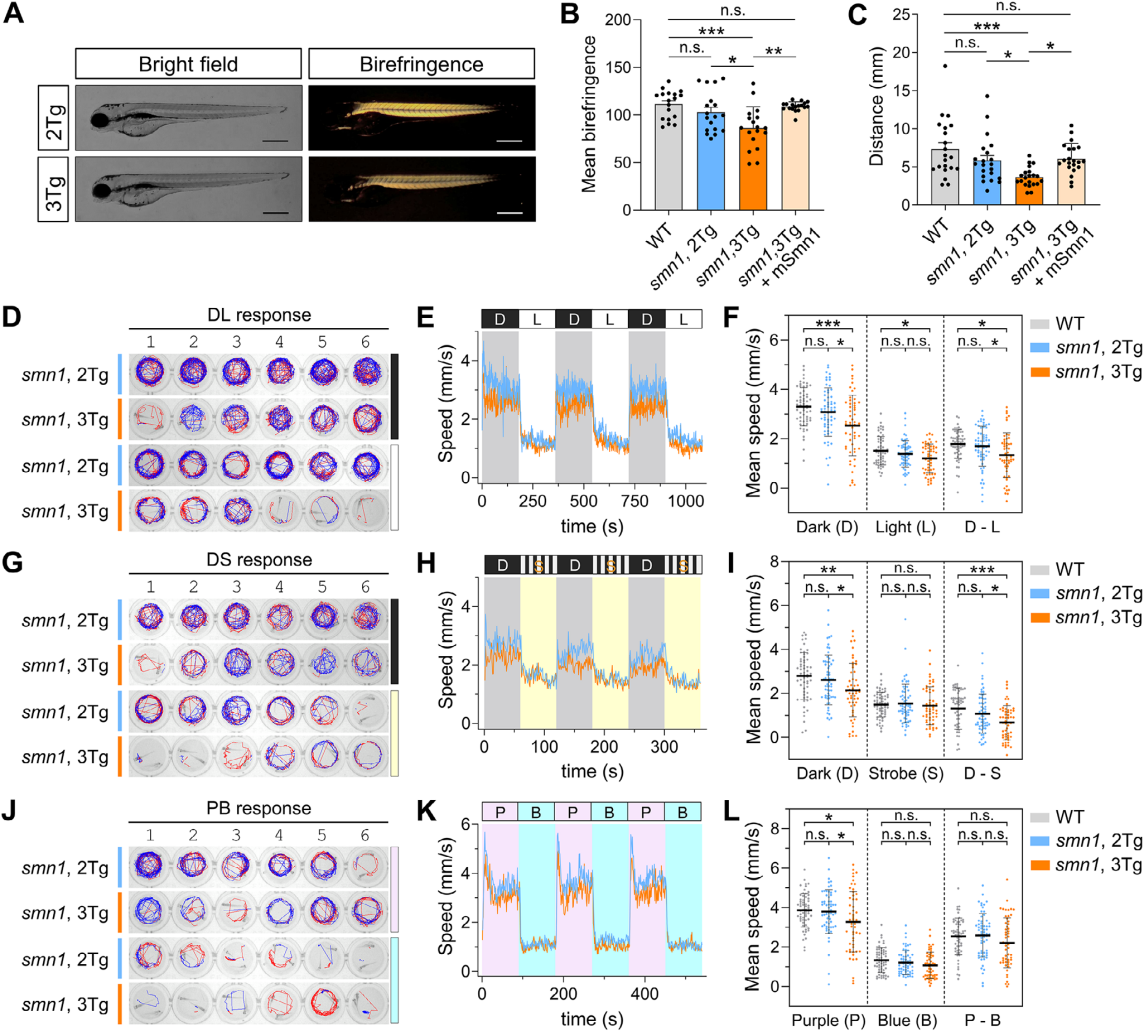
Knocking down *smn1* with CRISPR‐Q_KD_ results in behavioral deficits. The 3Tg embryos were obtained by crossing 2Tg zebrafish, Tg(*hsp70l*:QFvpr‐CK; 5×*QUAS*:CasRx‐2A‐EGFP‐CG), with transgenic zebrafish Tg(zU6:*smn1‐*AV). (A to C) Embryos underwent two heat shock treatments at 37°C for 1 h on 2 dpf, followed by two additional treatments at 38°C for 30 min on 3 dpf, with an 8‐h interval between the two heat treatments. Larvae were collected for analysis at 4 dpf. (D to L) Embryos were subjected to heat treatment starting at 3 dpf as described in Figure S9A, and were analyzed at 7 dpf. (A) Representative bright‐field and birefringence images of 2Tg or 3Tg embryos at 4 dpf. Scale bar, 500 µm. (B) The plot shows the quantitative analysis of birefringence of 2Tg and 3Tg embryos, as shown in A. *n* = 18 (number of animals). (C) Quantification of the touch‐evoked response for wild‐type, 2Tg, and 3Tg embryos. *n* = 21 (number of animals). mSmn1 refers to *smn1* mRNA containing mutations within the target site, synthesized by in vitro transcription, and used for the rescue experiments shown in panels B and C. Statistical analyses (B and C) were performed using one‐way ANOVA followed by Tukey's multiple comparisons test. ^*^ *p* < 0.05; ^**^ *p* < 0.01; ^***^ *p* < 0.001; n.s., not significant. (D–L) Behavioral analysis of locomotor activities in 2Tg and 3Tg larvae. Representative movement traces of two larvae per well (D, G, J), average speed curves for all samples under each behavioral condition (E, H, K), and corresponding quantitative scatter plots (F, I, L) are displayed for three behavioral settings: dark‐light cycle (D–F), dark‐strobe cycle (G–I), and purple‐blue cycle (J–L). (F, I, L) Each data point represents the average speed of two larvae in a single well during three specified cycles. Minus signs in D—L, D—S, and P—B denote the difference in average speeds between two specified cycles, reflecting changes in locomotor activity. Speed curves for 96 wells are presented in Figure S11A–C. D, dark; L, white light; S, strobe light; P, purple light; B, blue light. *n* = 60 for WT, *n* = 62 for 2Tg, *n* = 54 for 3Tg. Data are shown as mean ± s.e.m. (B, C) or mean ± s.d. (F, I, L). *P* values are derived from an unpaired two‐tailed *t*‐test. ^*^ *p* < 0.05; ^**^ *p* < 0.01; n.s., not significant.

We next asked whether CRISPR‐Q_KD_‐mediated knockdown of *smn1* could recapitulate phenotypes observed with MO knockdown or KO of *smn1*. The 3Tg embryos, Tg(*hsp70l*:QFvpr‐CK; 5×*QUAS*:CasRx‐2A‐EGFP‐CG; zU6:*smn1‐*AV) at 1 dpf, were subjected to daily heat treatment, and at 4 dpf, muscle integrity was assessed by birefringence, a physical property generated by the diffraction of polarized light through muscle sarcomeres [52]. While 2Tg larvae displayed typical bright birefringence, 3Tg larvae exhibited a slight but significant reduction in birefringence (Figure 4A,B), suggesting impairment in muscle. The next day, larval swimming behavior in response to tactile stimulation was observed in the touch‐evoked escape assay. Compared to 2Tg larvae, 3Tg larvae often displayed a weak or slow escape response, resulting in significantly reduced escape distance (Figure 4C). Similarly, 3Tg larvae showed significantly reduced birefringence and impaired escape response compared to larvae expressing gSmn1 alone (Figure S10A–C). Notably, the deficits in both birefringence and escape response were rescued by injection of *smn1* mRNA harboring mutations within the target site, confirming that these phenotypes resulted from *smn1* knockdown (Figure 4B,C).

To investigate potential motor function deficits, we examined whether 3Tg larvae exhibited impaired locomotor behaviors. Zebrafish larvae display specific movement patterns in response to various stimuli, including alternating dark and light [53, 54], alternating dark and 10‐Hz strobe light [55], violet light stimuli [56], and acoustic stimuli [56, 57]. We integrated these environmental stimuli into a custom‐built locomotion recording system that encompassed a series of light stimulation assays and a vibrational startle response assay (VSRA) (Figure S9). We sought to assess the behavioral response patterns of 2Tg and 3Tg larvae following *smn1* knockdown at 7 dpf. We observed overall slightly but significantly reduced locomotor activities in 3Tg larvae during dark conditions, and upon exposure to purple light (Figure 4D–L; Figure S11A–C). Similarly, 3Tg larvae showed a slight but significant reduction in VSRA compared to 2Tg larvae (Figures S10D,E and S11D and Movie S1). Larvae expressing gSmn1 alone displayed behavioral phenotypes comparable to wild‐type fish, whereas 3Tg larvae exhibited significant locomotor impairments (Figure S12A–D). These results demonstrate that CRIPSR‐Q_KD_‐mediated knockdown of *smn1* in zebrafish larvae sufficiently recapitulated the phenotypes associated with SMA diseases.

### 2.5 Double Knockdown of *Tardbp* and *Tardbpl* via CRISPR‐Q_KD_ Replicates ALS Features in Zebrafish

TAR DNA‐binding protein 43 (TDP‐43) is a widely expressed nuclear RNA/DNA‐binding protein that regulates diverse RNA metabolic processes, such as transcription repression, splicing, microRNA maturation, transport, stabilization, and translation [58, 59, 60, 61]. Dysregulation of TDP‐43 and its cytoplasmic deposits have been implicated in a variety of neurodegenerative diseases, including over 95% of ALS cases, ∼50% of frontotemporal lobar degeneration (FTLD), and more than 50% of Alzheimer's disease (AD) cases, from which limbic predominant age‐related TDP‐43 encephalopathy (LATE) has recently been characterized [62, 63]. This suggests that TDP‐43 is a common downstream pathogenic factor in a wide range of neurodegenerative diseases. TARDBP, the gene encoding TDP‐43, is highly conserved across species. While TDP‐43 deficient mice die during embryonic development [64], *tardbp* zebrafish mutants did not develop any overt phenotypes due to the presence of its paralogue *tardbpl* [65]. However, double homozygous mutants of *tardbp* and *tardbpl* exhibit various defects in muscle, motor neurons, and vasculature, leading to early death by 7–8 dpf [65]. Recent studies indicate that neuromuscular junction (NMJ) dysfunction and denervation precede motor neuron death in ALS pathogenesis [60, 66].

**FIGURE 5:**
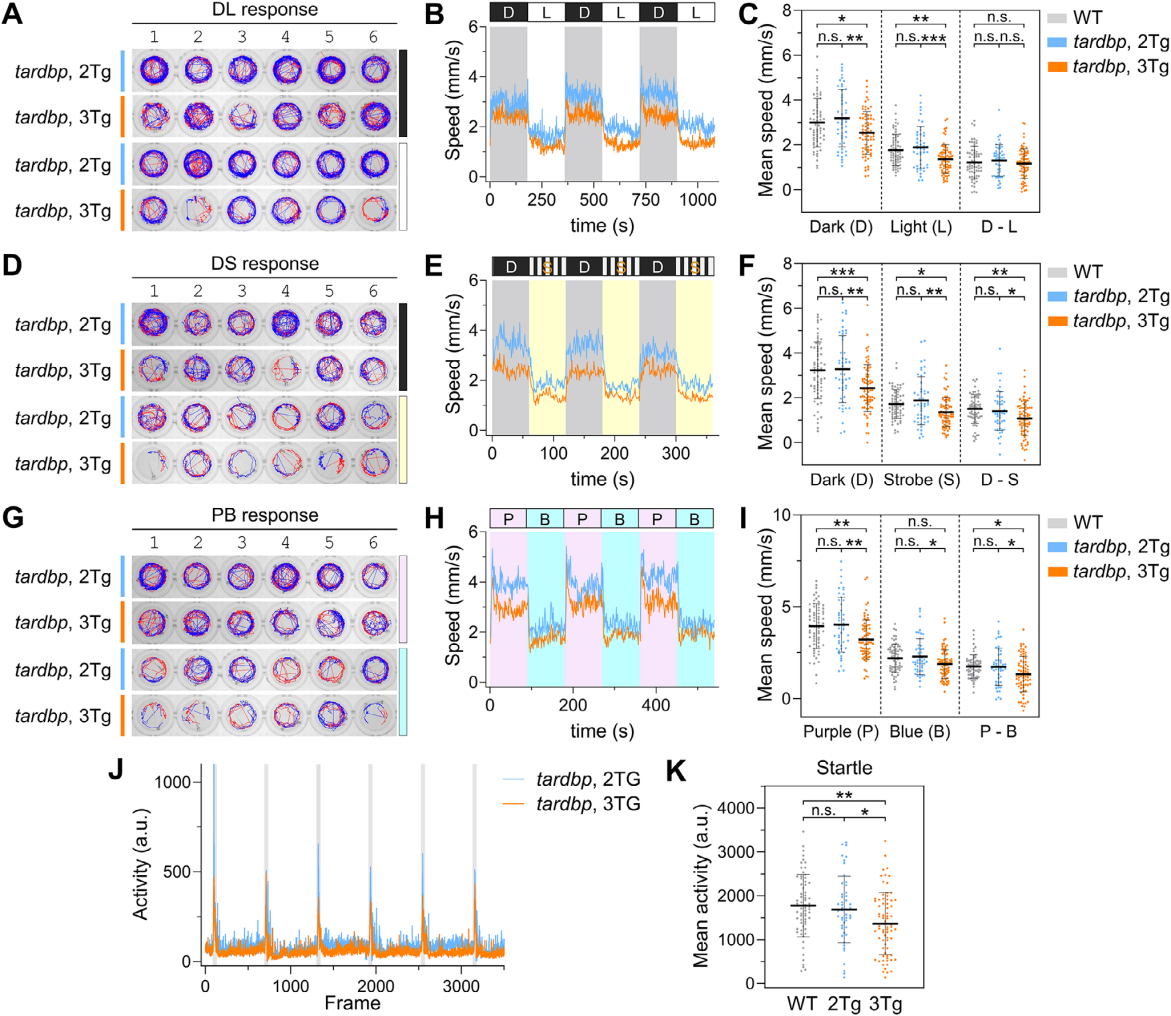
Simultaneous knockdown of two *tardbp* isoforms by CRISPR‐Q_KD_ leads to behavioral deficits. Embryos of 2Tg, Tg(*hsp70l*:QFvpr‐CK; 5×*QUAS*:CasRx‐2A‐EGFP‐CG) and 3Tg, which additionally express gRNAs targeting both *tardbp* and *tardbpl*, were subjected to daily heat treatment starting from 3 dpf as described in Figure S9A, and analyzed at 7 dpf. (A–K) Behavioral analysis of locomotor activities in 2Tg and 3Tg larvae. Representative movement traces of two larvae per well (A, D, G), average speed curves for all samples in each behavioral setting (B, E, H), and quantitative scatter plots (C, F, I) are displayed for three behavioral settings: dark‐light cycle (A–C), dark‐strobe cycle (D–F), and purple‐blue cycle (G–I). The average speed curves for all samples and the quantitative scatter plot of each well in the vibrational startle response assay (VSRA) are displayed in (J) and (K), respectively. Each data point represents the average speed of two larvae within a single well during three specified cycles (C, F, I) or the six maximum speeds within the 5‐frame period following six startle events for a single well (K). The gray bars in J indicate the vibrational tapping events. Minus signs in D—L, D—S, and P—B denote the difference in average speeds between two specified cycles, reflecting changes in locomotor activity. Speed curves for 96 wells are presented in Figure S11E–H. D, dark; L, white light; S, strobe light; P, purple light; B, blue light. *n* = 66 for WT, *n* = 48 for 2Tg, *n* = 73 for 3Tg (C, F, I, K). Data are shown as mean ± s.d. *P* values are derived from an unpaired two‐tailed *t*‐test. ^*^ *p* < 0.05; ^**^ *p* < 0.01; ^***^ *p* < 0.001; n.s., not significant.

**FIGURE 6:**
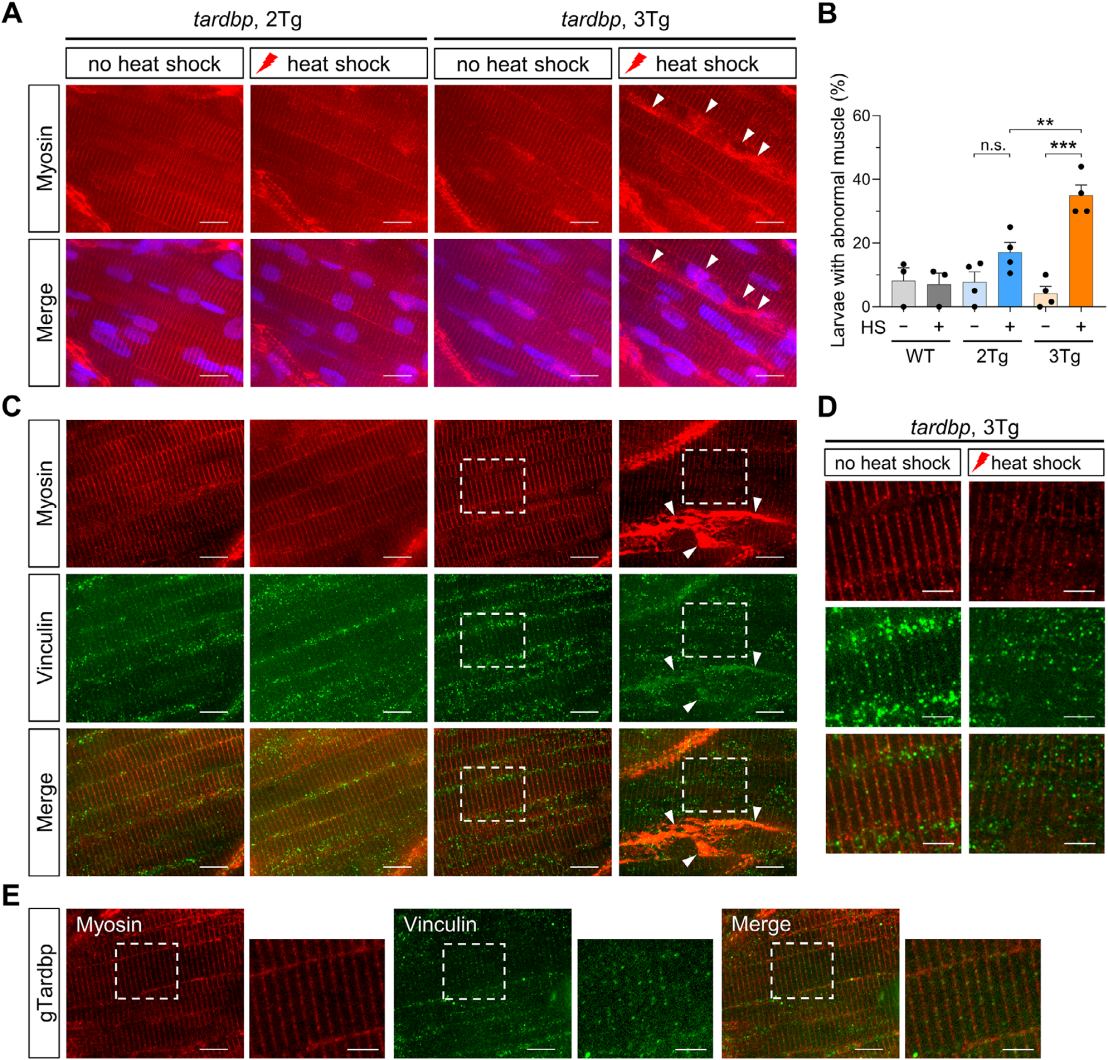
Simultaneous knockdown of two *tardbp* isoforms by CRISPR‐Q_KD_ leads to muscle impairment. (A) Immunostaining of 6‐dpf‐old 2Tg and 3Tg larvae, with or without heat treatment (37°C for 1 h at 2 dpf and 38°C for 1 h at 3–5 dpf), was performed using an antibody against myosin (MYH4) (red) to examine muscle integrity. (B) Quantitative analysis of immunostaining in 2Tg and 3Tg larvae as shown in A. The frequency of larvae with abnormal muscle staining was determined. *n* = 3 for WT, *n* = 4 for 2Tg and 3Tg. Data are presented as mean ± s.e.m. Each replicate was conducted with 20 larvae per condition. Statistical analyses were performed using one‐way ANOVA followed by Tukey's multiple comparisons test. ^**^ *p* < 0.01; ^***^ *p* < 0.001; n.s., not significant. (C) Representative images of immunostaining of 6‐dpf‐old 2Tg and 3Tg larvae, with or without heat treatment, using antibodies against myosin (MYH4) (red) and Vinculin (green). (A, C) White arrowheads indicate abnormal myocytes, defined by irregular or patchy MYH4 staining patterns and characterized by disrupted or aggregated sarcomeric structures. Scale bar, 10 µm. (D) Zoomed‐in images from the boxes shown in C. Scale bar, 5 µm. (E) Representative immunostaining images of 6‐dpf transgenic larvae expressing gRNA for *tardbp* following heat treatment. Scale bar, 10 µm. Zoomed‐in images from the white boxes are shown on the right, with a scale bar of 5 µm.

Given that both *tardbp* and *tardbpl* were significantly knocked down by CRISPR‐Q_KD_ (Figure 3D), we sought to observe motor dysfunction using this zebrafish model. Compared to 2Tg larvae, 3Tg larvae displayed significantly reduced locomotor activities in all light conditions, especially in the dark or under purple light, where zebrafish are typically more active (Figure 5A–I; Figure S11E–G), suggesting dysfunctional NMJ in 3Tg zebrafish larvae. Additionally, the double knockdown of *tardbp* and *tardbpl* led to a significant reduction in startle response (Figure 5J,K; Figure S11H and Movie S2). Zebrafish larvae carrying gTardbp alone did not show any noticeable phenotypic differences compared to wild‐type controls, whereas 3Tg larvae showed significant locomotor impairments (Figure S12E–H). Next, we assessed muscle integrity in 3Tg larvae by immunostaining for myosin heavy chain. Most larvae displayed a regular structural array of muscle fibers. However, we found a significant increase in abnormal or degenerating myocytes in 3Tg larvae heat‐treated for double knockdown of *tardbp* and *tardbpl*, compared to 2Tg or non‐heat‐shocked larvae (Figure 6A,B), consistent with the previous report showing muscle defects [65]. Abnormal myocytes were defined by irregular or patchy myosin heavy chain (MYH4) staining patterns, characterized by disrupted or aggregated sarcomeric structures, as indicated by white arrows. Occasionally, we observed severely disorganized (Figure 6C) or misaligned and unaligned (Figure 6D) muscle structures stained with the myosin heavy chain and vinculin, a focal adhesion protein present in the myotendinous junction. Notably, expression of gTardbp alone in zebrafish larvae did not affect muscle integrity (Figure 6E). Taken together, simultaneous knockdown of *tardbp* and *tardbpl* by CRISPR‐Q_KD_ results in phenotypic developments in zebrafish larvae, including impaired muscle and locomotor activity, features of ALS.

To test whether acute knockdown of *smn1* or *tardbp*/*tardbpl* via embryo injection could induce behavioral phenotypes at 7 dpf larvae, we injected embryos with mCasRx and gRNA targeting *smn1* or *tardbp*/*tardbpl* and measured locomotor activity at 7 dpf. No behavioral phenotypes were observed following acute injection in any of the assays (Figure S13A,B). Consistent with this, no significant knockdown of *smn1* or *tardbp/tardbpl* was detected at 4 dpf after acute injection. By contrast, Tg(zU6:*tardbp*/*tardbpl*) transgenic larvae injected with mCasRx retained slightly but significantly reduced *tardbpl* mRNA levels at 4 dpf, reflecting residual CasRx protein expression from mRNA injection (Figure S13C,D). Furthermore, gRNAs targeting either *smn1* or *tardbp/tardbpl* from injected embryos were nearly completely degraded by 4 dpf, whereas strong gRNA expression persisted in transgenic larvae at the same stage (Figure S13E,F). These results highlight the necessity of the CRISPR‐Q system for gene perturbation at later developmental stages, as conventional acute injection approaches are largely ineffective beyond 4 dpf.

### 2.6 CRISPR‐Qa Effectively Activates Transcription in Transgenic Zebrafish

**FIGURE 7:**
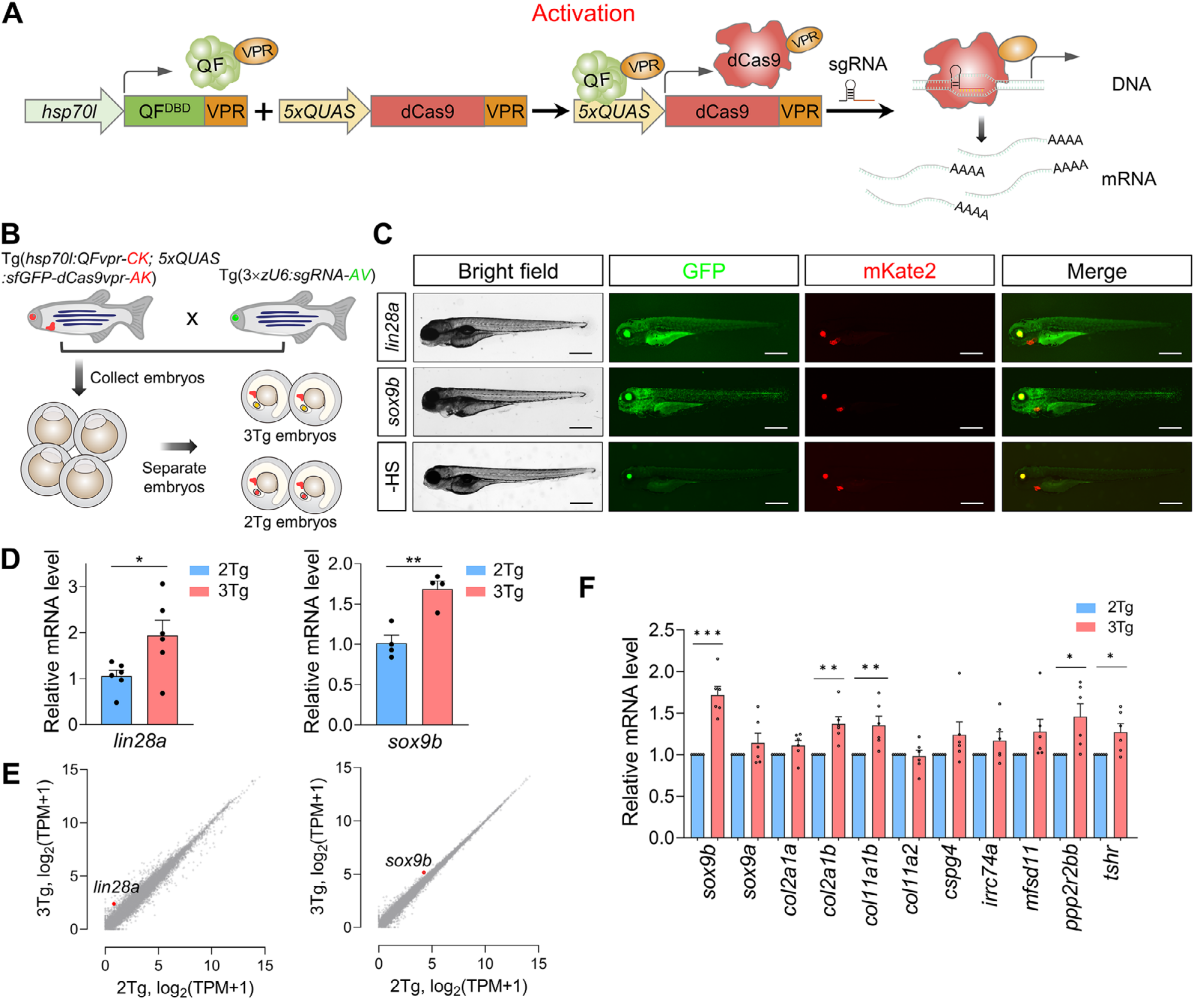
CRISPR‐Qa effectively upregulates endogenous genes in zebrafish. (A) Diagram illustrating the application of the CRISPR‐Qa‐induced gene activation. (B) Experimental procedure: The 2Tg zebrafish line, Tg(*hsp70l*:QFvpr‐CK; 5×*QUAS*:sfGFP‐dCas9vpr‐AK), was outcrossed with transgenic zebrafish expressing 3×sgRNAs targeting three sites in the promoter region of the *lin28a* or *sox9b* gene. The resulting offspring embryos were divided into 3Tg or 2Tg groups for subsequent experiments. CK, *cmlc2*:mKate2; AK, α‐*crystallin*:mKate2; AV, α‐*crystallin*:Venus. (C) Representative images of 3Tg embryos following heat shock treatment. Embryos underwent two heat shock treatments at 37°C for 1 h on 2 dpf, followed by two additional treatments at 38°C for 30 min on 3 dpf, with a 8‐h interval between the two heat treatments. Larvae were collected for analysis at 4 dpf. A heat shock‐free (‐ HS) larva derived from 3Tg *lin28a* line was included as a negative control. Scale bar, 500 µm. (D) The qPCR assays were conducted for *lin28a* (left) and *sox9b* (right). *n* = 6 for *lin28a* and *n = 4* for *sox9b*. (E) Scatter plots from RNA‐seq analysis showed minimal global changes in transcript abundance between 2Tg and 3Tg samples, except for noticeable upregulation in *lin28a* (left) and *sox9b* (right). Three independent biological replicates were performed for each condition. The red dots indicate *lin28a* (E, left) or *sox9b* (E, right) gene. (F) CRISPR‐Qa‐mediated upregulation of *sox9b* significantly enhanced the mRNA levels of target genes of *sox9b*. Note that *sox9a* was selected as a negative control. *n* = 6. Data are shown as mean ± s.e.m. Each replicate was performed with 20–30 pooled larvae (D–F). *P* values are derived from an unpaired two‐tailed *t*‐test. ^*^ *p* < 0.05; ^**^ *p* < 0.01; ^***^ *p* < 0.001.

Given that our enhanced QFvpr/QUAS binary system can drive substantially increased expression of the CasRx protein (Figure 3; Figure S4), we next investigated whether an increased level of the dCas9vpr activator could induce sustained transcription activation of endogenous genes (Figure 7A). To this end, *lin28a*, *myca*, and *sox9b* were chosen to test gene activation. LIN28 is an RNA‐binding protein that plays important roles in promoting stem cell metabolism and repressing microRNA biogenesis [67]. It is one of the reprogramming factors for somatic cells to iPSCs. Aberrant regulation of LIN28 has been shown to facilitate cancer proliferation as well as metastasis [68]. MYC is a proto‐oncogenic transcription factor that regulates a broad spectrum of cellular programs such as metabolism, immune response, cell proliferation and differentiation, stem cell biology, reprogramming, and more [69, 70]. SOX9 is a transcription factor involved in organ development, stem cells, regeneration, and human diseases, including cancers [71]. SOX9 regulates a wide range of cancer biology, including proliferation, invasion, metastasis, and stemness [72].

To increase the probability of transcription activation, we designed three sgRNAs per gene targeting the proximal promoter region of a gene of interest and cloned them into a single plasmid, generating a 3×zU6:sgRNA construct. We constructed three such plasmids, each targeting *lin28a*, *myca*, or *sox9b*, and established three transgenic zebrafish lines: Tg(zU6: *lin28a*), Tg(zU6:*myca*), and Tg(zU6:*sox9b*). Next, 3Tg embryos were obtained by crossing 2Tg zebrafish Tg(*hsp70l*:QFvpr‐CK; 5×*QUAS*:sfGFP‐dCas9vpr‐AK) with the respective transgenic zebrafish expressing the zU6:3×sgRNA construct targeting the gene of interest (Figure 7B). The resulting embryos underwent daily transient heat treatment starting at 2 dpf. Remarkably, the following day, we observed pronounced expression of dCas9vpr, as indicated by EGFP fluorescence (Figure S14A). Most of the embryos developed normally by 2 dpf, and the heat treatment could be repeated on subsequent days to extend the duration of gene regulation (Figure 7C; Figure S14B). It is noteworthy that we previously made significant efforts to generate the transgenic zebrafish line expressing dCas9vpr under the direct control of *hsp70l* promoter, but the transactivation levels of dCas9vpr from multiple founder lines were mostly modest or too weak (data not shown).

The qPCR results demonstrate that, in comparison to 2Tg condition, the expression levels of *lin28a* (1.94±0.33) and *sox9b* (1.69±0.010) are significantly higher in 3Tg zebrafish larvae (Figure 7D). The expression of *myca* was also modestly, though not significantly, increased (Figure S5B). Transcriptome profiles show that *lin28a* and *sox9b* are markedly upregulated (Figure 7E). Given the activation of *sox9b*, we investigated whether the upregulation of *sox9b* could activate its target genes. We tested the expression levels of nine genes that are reported to be regulated by *sox9b*. Among them, four genes were significantly upregulated, and the remaining genes showed a general trend of increased expression (Figure 7F). By contrast, the expression of *sox9a*, serving as a negative control, remained unchanged. Thus, our CRISPR‐Qa system can sufficiently enhance CRISPRa activity, making it possible to sustain functional activation of endogenous genes in transgenic zebrafish.

Next, we assessed the copy numbers of CRISPR‐Qa components for *sox9b* and *lin28a* and found some differences among transgenic lines as well as among embryos within the same batch. The *sox9b* line carried 1–2 copies of QFvpr, one copy of dCas9vpr, and one copy of the sgRNA, whereas the *lin28a* line carried 2–4 copies of QFvpr, 1–2 copies of dCas9vpr, and 4–5 copies of the sgRNA, indicating overall higher copy numbers in the *lin28a* transgenic line (Figure S8I,J). Interestingly, this copy number pattern appeared to correlate with gene activation levels: *lin28a* showed approximately twofold upregulation with higher variation, while *sox9b* showed 1.7‐fold upregulation with less variation. Although the *sox9b* 3Tg line contained only one copy of the sgRNA, its expression level was comparable to that observed in embryos injected with 10 pg of sgRNA (Figure S8F). These findings suggest that transgenic lines with higher copy numbers could further improve the efficiency of the CRISPR‐Qa system.

Having confirmed the gene regulatory capabilities of both CRISPR‐Q_KD_ and CRISPR‐Qa, we next examined whether expression of the CRISPR‐Q system induces cellular stress or cell death. To assess cell death, we performed TUNEL (terminal deoxynucleotidyl transferase dUTP nick end labeling) assays on transgenic larvae expressing CRISPR‐Q_KD_ or CRISPR‐Qa, with or without heat shock, and found no detectable cell death under any condition (Figure S15A). Additionally, we evaluated ER stress, ROS stress, and immune responses by measuring the expression of marker genes for each pathway in the larvae using RT‐qPCR. The analysis revealed no detectable stress responses (Figure S16A,C,E).

### 2.7 CRISPR‐Q Effectively Modulates Gene Expression in Juvenile and Adult Zebrafish

Zebrafish offer a range of advantages for high‐throughput screening, drug discovery, and in vivo imaging using embryos and larvae. They also serve as powerful models for studying human diseases such as cancers, cardiomyopathies, and psychiatric disorders, as well as biological processes like hematopoiesis and neural stem cell regulation, spanning from embryos to juvenile and adult stages [73, 74, 75, 76].

**FIGURE 8:**
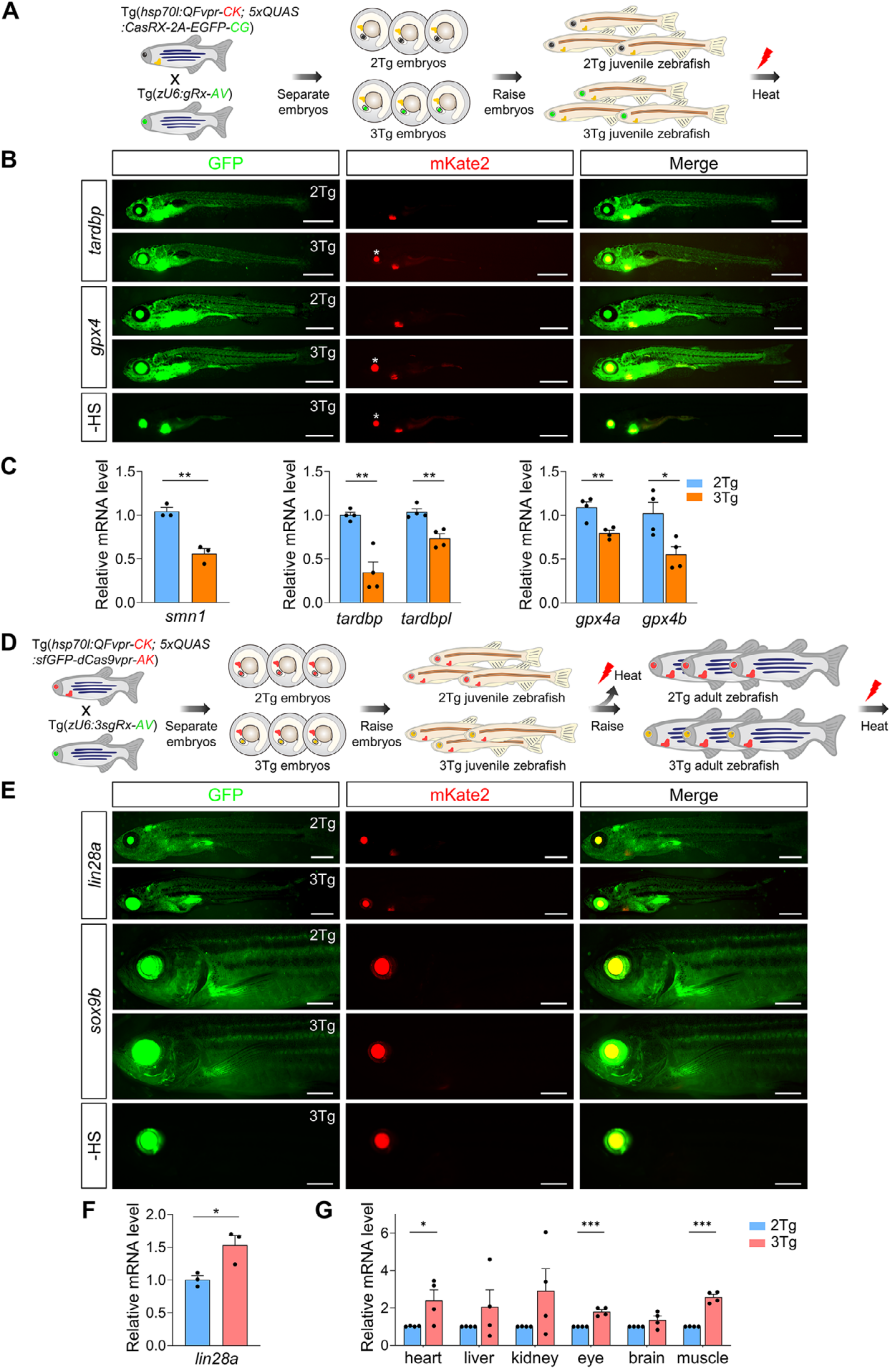
CRISPR‐Q efficiently regulates gene expression in juvenile and adult zebrafish. (A) Schematic of the CRISPR‐Q_KD_ gene knockdown procedure. The 2Tg zebrafish line, *Tg(hsp70l*:QFvpr‐CK; 5×*QUAS*:CasRx‐2A‐EGFP‐CG), was outcrossed with transgenic zebrafish expressing gRNAs targeting specific genes. Offspring embryos were sorted into 3Tg or 2Tg groups and raised to 2–3 weeks of age for downstream experiments. CK, *cmlc2*:mKate2; CG, *cmlc2*:EGFP; AV, *α‐crystallin*:Venus. (B) Representative images of 2Tg and 3Tg juvenile zebrafish following two heat shock treatments at 39°C for 1 h, with an 8‐h interval between treatments. Shown are 2‐week‐old *tardbp* and 3‐week‐old *gpx4* zebrafish. A heat shock‐free (‐ HS) 3‐week‐old zebrafish derived from 3Tg *gpx4* fish was included as a negative control. The asterisk denotes red fluorescence resulting from Venus signal bleed‐through. Scale bar, 1 mm. (C) qPCR analysis of *smn1* (left), *tardbp* (middle), and *gpx4* (right) expression. number of animals: *n* = 3 for *smn1*; *n* = 4 for *tardbp* and *gpx4*. Data are presented as mean ± s.e.m. (D) Schematic of the CRISPR‐Qa gene activation procedure. The 2Tg line, *Tg(hsp70l*:QFvpr‐CK; 5×*QUAS*:sfGFP‐dCas9vpr‐AK), was outcrossed with zebrafish expressing 3×sgRNAs targeting the promoter region of *lin28a* or *sox9b*. Offspring embryos were sorted into 3Tg or 2Tg groups and raised to 3–4 weeks or 3 months of age for analysis. AK, *α‐crystallin*:mKate2. (E) Representative images of 2Tg and 3Tg juvenile or adult zebrafish following two heat shock treatments at 39°C for 1 h, with an 8‐h interval between treatments. Shown are 4‐week‐old *lin28a* and 3‐month‐old *sox9b* zebrafish. A heat shock‐free (‐ HS) 3‐month‐old zebrafish derived from 3Tg *sox9b* fish was included as a negative control. Scale bars, 1 mm (*lin28a*); 2 mm (*sox9b*). (F, G) qPCR analysis of *lin28a* (F) and *sox9b* (G) expression. number of animals: *n* = 3 for *lin28a*; *n* = 4 for *sox9b*. Data are shown as mean ± s.e.m. *P* values were calculated using unpaired two‐tailed *t*‐tests. ^*^ *p* < 0.05; ^**^ *p* < 0.01; ^***^ *p* < 0.01.

Having established the effectiveness of the CRISPR‐Q system in zebrafish embryos and larvae, we next sought to determine whether it could achieve efficient gene modulation in older fish. We used juvenile and adult 2Tg and 3Tg zebrafish for CRISPR‐Q_KD_ and CRISPR‐Qa, followed by heat shock treatment (Figure 8A,D). Notably, we observed prominent GFP fluorescence the next day, indicating robust expression of CasRx or dCas9vpr, whereas control groups without heat shock showed only marker expression (Figure 8B,E). Importantly, no adverse effects, including apoptosis, ER stress, ROS stress, and immune responses, were detected in zebrafish after heat shock (Figures S15B,C and S16B,D,F and S17). Basal locomotor activity also remained unaffected following heat treatment (Figure S18). These results demonstrate that zebrafish can tolerate daily heat treatment over extended periods, highlighting the potential of the CRISPR‐Q system for long‐term applications.

We then assessed gene expression changes using RT‐qPCR. Remarkably, induction of CasRx led to significant reductions in the expression levels of *smn1* (0.56±0.059), *tardbp* (0.35±0.117), *tardbpl* (0.74±0.053), *gpx4a* (0.80±0.031), and *gpx4b* (0.56±0.087) in juvenile fish (Figure 8C), as well as *smn1* in liver, kidney, eye, and muscle tissues of adult fish (Figure S19). In contrast, activation of CRISPR‐Qa led to a significant upregulation of *lin28a* (1.53±0.147) in juvenile fish (Figure 8F). Furthermore, we observed a robust induction of *sox9b* in adult fish, with expression increased more than twofold in the heart (2.40±0.565) and muscle (2.57±0.151) tissues, while other tissues also showed a strong trend toward upregulation (Figure 8G).

These findings collectively demonstrate that the CRISPR‐Q system, driven by the heat shock promoter, can effectively modulate gene expression not only in early embryos and larvae but also in juvenile and adult zebrafish, thereby broadening its applicability for diverse studies.

### 2.8 CRISPR‐Q Enables Efficient Gene Regulation with Tissue‐Specific Expression

**FIGURE 9:**
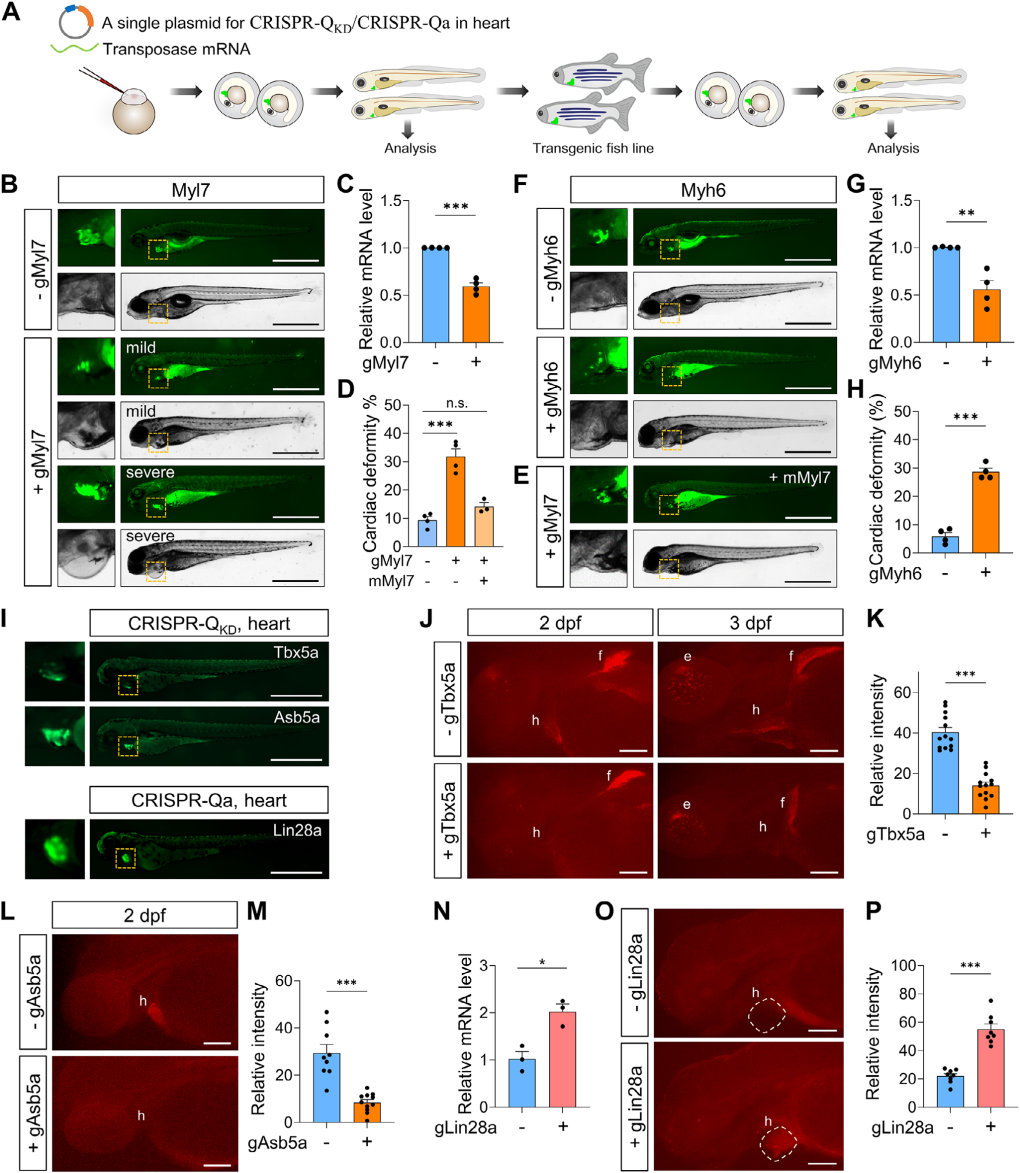
Tissue‐specific expression of CRISPR‐Q exhibits efficient gene regulation. (A) Schematic of CRISPR‐Q transgenic zebrafish generation using a single plasmid. Embryos at the one‐cell stage are injected with a single plasmid containing a tissue‐specific expression of QFvpr (e.g., *cmlc2*:QFvpr for heart expression), a Cas effector (e.g., 5×*QUAS*:CasRx/dCas9vpr‐2A‐EGFP), and gRNA/3×sgRNA. Embryos can be analyzed after injection or raised to establish transgenic zebrafish lines. (B, E, F) Representative images of larvae at 5 dpf injected with plasmids containing gMyl7 (B), gMyl7 and mMyl7 (E) or gMyh6 (F), or without gRNA. Zoomed‐in images from the boxed regions are shown on the left. Injected embryos often exhibit GFP fluorescence in the yolk. Scale bar, 1 mm. (C, G) RT‐qPCR analysis of *myl7* (C) and *myh6* (G) expression. *n* = 4. (D, H) Phenotypic analysis of cardiac deformation in larvae injected with gMyl7, gMyl7 and mMyl7 (D) or gMyh6 (H). *n* = 4 for ±gMyl7, *n* = 3 for +gMyl7 and mMyl7 in (D), *n* = 4 (H). mMyl7 denotes the in vitro‐transcribed mRNA of Myl7 containing mutations at the target sites. Each replicate included approximately 30 larvae per condition for analysis. (I) Representative images of F1 transgenic zebrafish larvae expressing CRISPR‐Q system in the heart, with corresponding gRNAs. Shown are Tbx5a and Asb5a CRISPR‐Q_KD_ lines at 3 dpf for knockdown and Lin28a at 4 dpf for gene upregulation by CRISPR‐Qa. Zoomed‐in images from the boxed regions are shown on the left. The green fluorescence observed in the body and yolk is attributed to autofluorescence. Scale bars, 1 mm. (J, L) Representative images of HCR staining for Tbx5a (J) and Asb5a (L) CRISPR‐Q_KD_ lines. h, heart; e, eye; f, fin. Scale bars, 100 µm. (K, M) Quantitative analysis of HCR staining for Tbx5a (K) at 3 dpf or Asb5a (M) at 2 dpf. number of animals: *n* = 13 (K); *n* = 9 for –gAsb5a, *n* = 11 for +gAsb5a in (M). (N) RT‐qPCR analysis of *lin28a* from transgenic embryos at 3 dpf with or without gLin28a. *n* = 3. (O) Representative images of HCR staining for Lin28a at 3 dpf in CRISPR‐Qa transgenic embryos. Heart chambers are marked with dotted lines. h, heart. Scale bars, 100 µm. (P) Quantitative analysis of HCR staining for Lin28a at 3 dpf. *n* = 8 (number of animals). Data in bar graphs are shown as mean ± s.e.m. Each replicate for RT‐qPCR was performed with 20–30 pooled larvae in (C, G, N). *P* values were calculated using unpaired two‐tailed *t*‐tests, except for panel C, which was analyzed using one‐way ANOVA followed by Tukey's multiple comparisons test. ^*^ *p* < 0.05; ^**^ *p* < 0.01; ^***^ *p* < 0.01; n.s., not significant.

Tissue‐specific expression is a critical component for transgenesis. After demonstrating a proof of concept for the CRISPR‐Q system under the control of the heat shock promoter, we next tested its efficiency in a tissue‐specific context. To facilitate this, we engineered a single plasmid containing the CRISPR‐Q system, which incorporates tissue‐specific expression of QFvpr driven by the *cmlc2* promoter for heart expression (*cmlc2*:QFvpr), a Cas effector (e.g., 5×*QUAS*:CasRx/dCas9vpr‐2A‐EGFP), and gRNA/3×sgRNA (Figure 9A; Figure S20B).

We selected *myl7* (a.k.a. *cmlc2*) and *myh6*, both heart‐specific genes crucial for heart development [77, 78, 79, 80], as targets for CRISPR‐Q_KD_. Following microinjection of the CRISPR‐Q_KD_ plasmid targeting *myl7* or *myh6*, embryos exhibiting GFP expression in the heart were selected for heart morphology analysis and mRNA level assessment (Figure 9B–G). Remarkably, larvae expressing CRISPR‐Q_KD_ with gMyl7 (0.59±0.162) or gMyh6 (0.56±0.247) exhibited significantly reduced mRNA levels compared to controls (Figure 9C,G). In addition, we observed a significantly higher incidence of cardiac deformation in larvae injected with CRISPR‐Q_KD_ containing the gRNA compared to those without gRNA (Figure 9D,H), consistent with previous reports [77, 78, 79, 80], and this cardiac defect in larvae expressing CRISPR‐Q_KD_ for *myl7* was rescued by co‐injection of *myl7* mRNA containing mutations at the target sites (Figure 9D,E). These results indicate that CRISPR‐Q_KD_ can be efficiently applied for transient, tissue‐specific gene modulation via microinjection, enhancing throughput, facilitating phenotypic analysis, and enabling mosaic expression studies.

Next, we generated heart‐specific transgenic zebrafish lines targeting *tbx5a* and *asb5a* for CRISPR‐Q_KD_ and *lin28a* for CRISPR‐Qa. To achieve more efficient knockdown, we first screened gRNAs targeting *tbx5a* and *asb5a*. Individual gRNAs showed variable knockdown efficiency, ranging from 30% to 50%. However, combining two gRNAs increased knockdown compared to each single gRNA (Figure S21). These results underscore the importance of selecting optimal target sites and applying multiple gRNAs per gene to enhance knockdown efficiency.

All these three CRISPR‐Q lines exhibited robust expression (Figure 9I), and no adverse effects were observed in the Tbx5a line (Figures S15A and S16A,C,E). To assess tissue‐specific expression of the target genes, we performed Hybridization Chain Reaction (HCR) staining, which allows highly sensitive and precise detection with high‐resolution imaging. As reported, we observed strong *tbx5a* signals in the heart and fin at 2 dpf, with additional eye staining at 3 dpf (Figure 9J) [81]. Notably, embryos expressing CRISPR‐Q_KD_ with gTbx5a (0.35±0.043) exhibited significantly reduced signal in the heart, while staining intensity in other tissues remained similar (Figure 9J,K). Similarly, HCR staining for *asb5a* showed heart‐specific expression, which was almost completely lost in larvae expressing CRISPR‐Q_KD_ with gAsb5a (0.29±0.041) (Figure 9L,M).

For tissue‐specific CRISPR‐Qa, we first measured *lin28a* expression using RT‐qPCR, as it is known to be expressed at very low levels at 3 dpf [82], allowing relatively accurate assessment without isolating the heart. As expected, we observed a significant upregulation of *lin28a* in embryos expressing CRISPR‐Qa with gLin28a (2.02±0.161) (Figure 9N). We then performed HCR staining for *lin28a*, confirming a remarkable increase in heart‐specific expression in embryos expressing CRISPR‐Qa with gLin28a (2.50±0.141), whereas embryos expressing only CRISPR‐Qa without gLin28a showed no detectable heart signal (Figure 9O,P).

After demonstrating the heart‐specific promoter, we next tested an additional promoter to further assess system versatility using an acute injection approach. We first screened six gRNAs targeting *tyr*, which encodes tyrosinase and is required for melanin synthesis. These gRNAs exhibited highly variable activities, with gTyr‐6 showing the most robust knockdown, underscoring the importance of gRNA screening for optimal efficiency (Figure S22A). Because embryos injected with gTyr‐2, gTyr‐3, and gTyr‐4 displayed only modestly reduced pigmentation phenotypes, whereas gTyr‐6 induced a strong phenotype (Figure S22B), we selected gTyr‐4 and gTyr‐6 for subsequent experiments. We then constructed a single plasmid containing *ubb*:QFvpr, *5×QUAS*:CasRx‐2A‐EGFP, and zU6:*tyr* (or 2*×tyr)* gRNAs, carrying either gTyr‐6 alone or a combination of gTyr‐4 and gTyr‐6 (gTyr‐4+6). Notably, embryos injected with plasmids carrying either gTyr‐6 or gTyr‐4+6 exhibited a noticeable reduction in pigmentation at 48 hpf, despite the mosaic expression typically observed in injected embryos (Figure S22C). These results demonstrate that a ubiquitously expressed, *ubb* promoter‐driven CRISPR‐Q_KD_ system can effectively induce gene knockdown, further supporting its applicability in zebrafish.

Taken together, these results demonstrate that the CRISPR‐Q system can efficiently achieve tissue‐specific gene modulation in transgenic zebrafish, thereby expanding its utility for transgenic applications.

## 3 Discussion

Since the emergence of the CRISPR system as a powerful tool for gene editing, various CRISPR systems have been created for gene regulation, including CRISPRa/i and new Cas proteins that target RNA for degradation, such as CasRx. However, in zebrafish, these systems have only been used for acute injections, which limits their usefulness for studying gene function or modeling human diseases at later stages or in spatial or temporal contexts. A significant challenge in achieving this lies in ensuring a consistent and strong expression of the Cas effector protein in zebrafish. To address this, employing a robust binary expression system is pivotal. However, the conventional Gal4/UAS binary system has exhibited limitations in zebrafish transgenesis, characterized by notable toxicity and transgene silencing. We have developed an enhanced binary expression system called QFvpr/QUAS, engineered to induce and control robust expression of Cas effectors in transgenic zebrafish. We have successfully demonstrated that QFvpr/QUAS‐driven Cas effectors, CRISPR‐Q_KD,_ and CRISPR‐Qa, can be used for gene regulation, such as transcript knockdown and gene activation. This improved CRISPR system offers great potential for researchers studying gene function or modeling human diseases in zebrafish. It allows for unprecedented precision and control over gene expression, making it a valuable tool for advancing their research.

CasRx expression during early zebrafish embryonic development can be harmful to the embryos. While acute injection of CasRx and gRNA may not exhibit overt toxicity [18], this could be attributed to the degradation of both the CasRx protein/mRNA and gRNA. We have occasionally observed toxicity in transgenic zebrafish expressing CasRx at elevated levels, with 3Tg larvae appearing more sensitive. This is likely due to the collateral activity of CasRx, although it was not detected in our experiments (Figure S7), consistent with recent studies in mice showing that CasRx can be toxic and lethal in transgenic mice due to unintended gene damage via collateral activity [44, 83]. Notably, CasRx has been reported to induce lethality on its own, even without gRNA, when expressed at high and widespread levels [83]. Tong and colleagues have engineered high‐fidelity Cas13 variants, hfCas13d (derived from CasRx) and hfCas13X (derived from Cas13X), which exhibit no discernible collateral effects, resulting in no toxicity or lethality in mice [83]. Additionally, a Cas13 variant with enhanced activity has been developed through structure‐guided engineering [84]. Given these advancements, it would be highly intriguing to evaluate the efficacy of these high‐fidelity Cas13 variants for transcript knockdown in the zebrafish transgenic system, particularly in combination with the QFvpr/QUAS system.

Regarding transcript knockdown, we observed that the efficiency of CasRx in RNA knockdown exhibited variability and appeared to be less potent in transgenic zebrafish compared to cell experiments, although all gRNAs were designed using a cutting‐edge CasRx activity prediction tool [85]. Furthermore, we occasionally encountered instances of insufficient knockdown by CasRx, even in cell experiments. This highlights the need for improvement of prediction algorithms to effectively identify the most efficient gRNAs and underscores the importance of testing multiple gRNAs to ensure the desired outcome. We also highly recommend using two to three gRNAs per gene to ensure efficient knockdown, as supported by our findings (Figure 9J–M). Indeed, previous studies employing acute CasRx‐mediated knockdown have consistently used three gRNAs per gene [18, 43].

The relationship between gRNA dose and knockdown efficiency in transient Cas13d delivery systems is likely influenced by both temporal dynamics and saturation effects. At early time points following injection, Cas13d and gRNAs may approach functional saturation, limiting the ability to detect a linear dose–response relationship. At later stages, as both gRNAs and Cas13d proteins or mRNAs undergo degradation, differences in gRNA dosage may become more apparent; however, this effect can be confounded by multiple factors, including variable gRNA stability, differences in gRNA quality, RNP formation efficiency, and gRNA target site selection. Consistent with this complexity, previous studies have reported gRNA dose dependence at early time points (4–6 hpf) using doses ranging from 300 pg to 1 ng, suggesting that functional saturation may not be complete even at 300 pg [18, 43]. Together with our findings (Figure S8B–E), these observations indicate that the optimal window for detecting gRNA titration effects is context dependent. Overall, these considerations highlight the complexity of interpreting gRNA dose–response relationships in transient knockdown experiments and underscore the need for cautious interpretation of titration data.

Commencing the development of the QFvpr/QUAS binary expression system, we initially opted for two separate components, QFvpr and QUAS, anticipating greater flexibility. However, we subsequently encountered some challenges with this approach. First, generating 2Tg zebrafish is time‐consuming and requires a subsequent cross with a zU6‐gRNA transgenic zebrafish line. Second, the expression levels of 2Tg or 3Tg zebrafish lines can be quite variable due to variability in the number of copies of each transgene. Based on these observations, we recommend adopting a single‐plasmid binary system that combines QFvpr and 5×QUAS‐Cas effector (CasRx or dCas9vpr) into a single Tol2 plasmid (Figure S20A). Furthermore, incorporating zU6‐gRNA into a single‐plasmid binary system (Figure S20B) allows for the injection of one plasmid containing all three components (QFvpr; 5×*QUAS*‐CasRx/dCas9vpr; zU6‐gRNA/3×U6‐sgRNA) into zebrafish embryos. This streamlined approach not only saves time but also has the potential to yield more reliable results.

We also found that the average knockdown efficiency in transgenic fish is lower than that typically achieved by transient injection. Nevertheless, despite relatively low knockdown efficiencies (∼30%–40%) for *smn1* and *tardbp*, these levels were sufficient to induce mild but detectable phenotypes, which may be advantageous for studying genes or disease models with severe phenotypes or early lethality (e.g., *smn1* and *tardbp* mutants, which survive only 4–8 dpf). However, such knockdown levels may be insufficient to elicit clear phenotypes for other genes. To improve knockdown efficiency in transgenic fish, we propose several strategies, including screening for the most effective gRNAs, combining multiple optimal gRNAs, and increasing CasRx expression levels, all of which are expected to enhance overall knockdown efficiency. In the present study, we utilized five repeats of the QUAS element (5×QUAS), as it provides activation levels comparable to 14×UAS in transient experiments. Given that QUAS is more resistant to gene silencing than UAS, it is highly plausible that increasing the number of QUAS repeats (e.g., to 10× or 15×) could further enhance the transactivation of Cas effector proteins. Additionally, our current constructs were not codon‐optimized for zebrafish, as each component was originally designed for mammalian systems. Expression levels of the CRISPR‐Q system, particularly CRISPR‐Qa, can sometimes be suboptimal. Codon optimization of the CRISPR‐Q components, especially the Cas effectors, may further improve expression efficiency in zebrafish. To demonstrate this, we synthesized a zebrafish codon‐optimized CasRx (zcoCasRx) using a recently developed algorithm for codon optimality [86] and compared its knockdown efficiency with that of CasRx through acute mRNA injection. We observed a modest but noticeable trend toward improved knockdown with zcoCasRx (Figure S23).

Regarding CRISPRa, the current version of CRISPR‐Qa employs three zU6‐driven sgRNAs per target gene. Generally, effective modulation of gene expression using CRISPRa or CRISPRi requires at least three sgRNAs and often benefits from five to ten. Therefore, increasing the number of sgRNAs to target additional regions may further enhance the transactivation efficiency of CRISPR‐Qa.

Together, these considerations highlight the potential for further refinement and improvement of the CRISPR‐Q system.

Beyond its current applications demonstrated here, our CRISPR‐Q system holds promise for CRISPR interference (CRISPRi) in zebrafish, and possibly in other organisms, a capability not yet demonstrated in transgenic zebrafish models. Typically, achieving a noticeable alteration in gene expression with CRISPRa or CRISPRi requires the use of multiple sgRNAs. To introduce multiple sgRNAs in transgenic zebrafish, it is necessary to construct a Tol2 plasmid containing multiple repeats of the RNA polymerase III‐dependent U6 promoter, with each promoter driving a distinct sgRNA. However, this process can be laborious and may be susceptible to recombination in *E. coli*. Moreover, introducing multiple promoters at a single locus in transgenic zebrafish could potentially result in unforeseen intricacies in gene expression, potentially including gene silencing. To address these challenges, several systems have been developed to facilitate the delivery of multiple sgRNAs, including Csy4 [87], ribozyme [88], and tRNA‐based multiplex [89]. Unlike others, tRNA‐based multiplexing is particularly appealing because it can produce more than three sgRNAs without reducing their activity or inducing toxicity [89]. The integration of our CRISPR‐Q with tRNA‐based multiplexing has the potential to simplify the generation of transgenic zebrafish systems for efficient CRISPRa or CRISPRi.

CRISPR‐Cas systems not only regulate gene expression but also offer tools to study specific RNA transcripts in various cellular contexts and modulate RNA processes, including splicing, nuclear export, RNA degradation and stability, subcellular localization, and translation [15, 90]. One crucial layer of RNA regulation involves methylation, particularly the N6‐methyladenosine (m^6^A) modification. The m^6^A‐modification is a reversible process mediated by three groups of proteins: methyltransferases (writers, e.g., METTL3), demethylases (erasers, e.g., FTO and ALKBH5), and binding proteins (readers).

To study the roles of RNA methylation, knockout or overexpression of these proteins has been commonly used. However, these approaches affect a large number of m^6^A‐modified RNAs, leading to complex biological outcomes or unintended consequences. Understanding the role of m^6^A modifications in individual RNA transcripts from a gene of interest can provide deeper insights into the mechanisms of signaling pathways in pathogenic conditions, such as cancer, and their roles in biological processes, such as development, stem cell biology, and neuronal functions. While depleting or overexpressing m^6^A modifiers provides valuable insights, it lacks precision, hindering the understanding of specific m^6^A sites on individual transcripts.

Various forms of catalytically dead Cas13 or Cas9 (dCas13 or dCas9) have been linked with m^6^A writers, erasers, or readers to genetically program m^6^A modification at specific sites via gRNA [91]. Zebrafish is an excellent model for applying these CRISPR‐Cas methods to discover the role of m^6^A modifications in individual RNA transcripts from a gene of interest in an in vivo setting. This approach requires sustained and robust expression of the Cas effector protein with spatiotemporal control, for which the CRISPR‐Q system is particularly well‐suited.

In addition to QFvpr, two new QF systems have been recently developed. One incorporates the original Gal4 activation domain to mitigate toxicity, resulting in QFGal4 [36], while the other is combined with the ecdysone receptor for temporal regulation [37]. This latter system is called IQ‐Switch, and it can be activated by the ecdysone agonist, tebufenozide. QFvpr is well‐tolerated because it does not contain the QF AD domain, which is known to be a major cause of toxicity after the middle domain is removed [36, 37]. However, we observed some toxicity at very high expression levels. It would be intriguing to investigate whether QFGal4 offers improved tolerability while maintaining sufficient robustness for effective modulation of CRISPR effectors for gene knockdown or upregulation to a comparable degree as QFvpr. IQ‐Switch presents an enticing tool for controlling transactivation in a spatiotemporal manner in zebrafish transgenesis. Therefore, further exploration of IQ‐Switch for the application of CRISPR effectors could prove to be highly valuable.

## 4 Conclusions

In this study, we establish a proof of concept for the effectiveness of enhancing the expression of CRISPR effectors using the QFvpr/QUAS binary expression system, thereby generating CRISPR‐Q, to achieve sustained and potent modulation of gene activation or downregulation in zebrafish. To the best of our knowledge, this application has not been previously demonstrated in this animal model. While the CRISPR‐Q system was initially designed for zebrafish transgenesis, its potential extends to various model organisms, including *C. elegans*, fruit flies, and mammals. CRISPR‐Q holds the promise of yielding enhanced outcomes for knockdowns, gene activation, or downregulation, as it amplifies the expression levels of CRISPR‐Cas effectors. Moreover, the QFvpr/QUAS binary system offers an efficient solution for research scenarios that demand sustained, robust expression levels of a target gene.

## 5 Experimental Section

### 5.1 Zebrafish Husbandry

Wild‐type AB line and all transgenic zebrafish were maintained under standard conditions at 28.5°C, with a 14‐h light/10‐h dark cycle. The embryos used in each experiment were collected from multiple matings involving one male and one to two females, and were placed in zebrafish E3 medium (5 mM NaCl, 0.17 mM KCl, 0.33 mM CaCl_2_, and 0.33 mM MgSO_4_) and raised in a 28.5°C incubator with a light‐dark cycle of 14 h light and 10 h dark. All animal procedures were approved by the Animal Care and Use Committee of Wuhan University (No. AF078).

### 5.2 Construction of Plasmids

All molecular cloning procedures were conducted using Gibson (HiFi) assembly (E2621S, New England Biolabs, USA). The QFvpr, QF2, KalTA4, Gal4vpr, or a Gal4 variant with varying numbers of VP16 repeats, and NLS was integrated into the NotI and XhoI sites in the pTol2EGFPpA vector, which contains a CMV promoter as well as Tol2 transposon arms and was engineered in our laboratory from the pCMV‐Tag3B vector [92]. These constructs were then utilized for a luciferase assay in HEK293T cells. For zebrafish transgenesis, *cmlc2*:mKate2 (CK) elements were incorporated into the pTol2EGFPpA vector at the BglII and MluI sites. The *cmlc2* promoter will allow the expression of mKate2 in the heart from 1 day postfertilization (dpf), serving as a discernible marker. Next, a CMV promoter was replaced with either the *ubb* or *hsp70l* promoter, which were integrated into SpeI and NheI sites, or into SpeI and SacII sites, respectively, upstream of QFvpr. This resulted in the generation of pTol2‐*ubb*‐QFvpr‐CK or pTol2‐*hsp70l*‐QFvpr‐CK, respectively.

To generate pTol2 plasmids expressing CasRx under the control of a 5×QUAS promoter, 5×QUAS, CasRx containing two NLS sequences, and 2TA‐EGFP were PCR‐amplified and co‐inserted into AgeI and EcoRI sites in pTol2EGFPpA. Subsequently, *cmlc2*:EGFP (CG) was inserted as a selection marker into BglII and MluI sites, resulting in the creation of the plasmid pTol2–5×*QUAS‐*CasRx‐T2A‐EGFP‐CG. To express dCas9vpr, PCR amplification was employed to amplify 5×QUAS, *ubc* intron to enhance expression [93], and 3×NLS‐sfGFP. These elements were then co‐inserted into the AgeI and BamHI sites in pTol2EGFPpA. Subsequently, *α‐crystallin*:mKate2 (AK) was integrated as a selection marker into BglII and MluI sites, enabling mKate2 expression in the lens starting from 3 dpf. Finally, PCR‐amplified dCas9 and VPR were co‐inserted into BamHI and SalI sites, leading to the formation of the pTol2–5×*QUAS*‐sfGFP‐dCas9vpr‐AK construct.

To activate transcription using dCas9vpr, we first inserted *α‐crystallin*:mVenus (AV) into the BglII and MluI sites of the pTol2EGFPpA vector as a selection marker. Following this, two zebrafish U6 promoters, namely zU6‐3 and an additional U6 promoter called zU6‐1s, each equipped with two unique type IIS restriction enzyme sites, were integrated in the order of zU6‐3‐EarI, zU6‐1‐BsaI, and zU6‐3‐BsmBI into the AgeI and XhoI sites. To add enzyme sites, each U6 promoter was PCR amplified with a reverse primer containing two of the same type IIS enzyme sites in a divergent direction. Three fragments from PCR were assembled by the HiFi assembly method. This process led to the formation of the pTol2‐3×zU6‐sgRNA‐AV construct. To drive the expression of gRNAs for the CasRx system, zU6‐3, along with a DNA fragment containing the direct repeat (DR) sequence from the CasRx system and two BsmBI sites for gRNA insertion, was incorporated into the AgeI and XhoI sites within pTol2‐3×zU6‐sgRNA‐AV. This resulted in the creation of the plasmid pTol2zU6‐gRx‐AV by replacing 3×zU6‐sgRNA with zU6‐gRx. All gRNAs were then inserted into the respective type IIS restriction enzyme sites using Golden Gate Cloning. Briefly, two primers containing overhang sequences for ligation were ordered from Sangon Biotech (China) and annealed by heating followed by slow cooling. The corresponding plasmid was digested with a type IIS restriction enzyme and purified with a PCR column purification kit. Annealed oligos and the digested plasmid were then ligated using T4 DNA ligase (C301‐01, Vazyme, China). For the cloning of 3×zU6‐gRNA, one sgRNA was inserted at a time as described above, followed by two additional rounds of cloning to include all three sgRNAs. To design sgRNAs for Cas9, CHOPCHOP (https://chopchop.cbu.uib.no/) was used, and for designing gRNAs for CasRx or Cas13X, cas13design (https://cas13design.nygenome.org/) was utilized. Details regarding all gRNAs can be found in Table S1.

### 5.3 In Vitro Transcription of mRNAs and gRNAs

To generate mRNAs for injection, coding sequences of genes of interest were cloned into plasmids downstream of a T7 or T3 promoter. Linearized plasmids were then subjected to in vitro transcription using the T7 or T3 mMESSAGE mMACHINE kit (AM1344 or AM1348, Invitrogen, USA). To synthesize gRNAs for injection, DNA templates were generated by fill‐in PCR using a universal primer containing a T7 promoter. For sgRNAs targeting the *sox9b* promoter, the spacer sequence was cloned into the DR274 plasmid, which contains a T7 promoter and an sgRNA scaffold. The resulting DNA templates or linearized plasmids were transcribed in vitro using the T7 High Yield RNA Transcription Kit (TR101, Vazyme, China).

### 5.4 Microinjection and Generation of Transgenic Zebrafish

To generate transgenic zebrafish, 25 pg of Tol2 transposase mRNA and 25 pg of a plasmid containing the desired transgene were injected into one‐ or two‐cell stage embryos. Plasmids were purified using phenol‐chloroform extraction before preparing the injection mix for microinjection. Tol2 transposase mRNA was produced by inserting the T7 promoter into the pCS2FA‐transposase plasmid, thereby creating the pCS2T7‐transposase plasmid. The linearized pCS2T7‐transposase was transcribed in vitro. Among the injected embryos, those exhibiting fluorescence in the heart at 1–2 dpf or in the lens at 3–4 dpf were collected for further breeding. The founder zebrafish underwent screening through outcrossing with the wild‐type AB line, and the resulting F1 positive embryos were raised for subsequent experiments.

### 5.5 Cell Culture and Transfection

HEK293T cells were cultured in Dulbecco's modified Eagle's medium (DMEM) with high glucose, 2 mM glutamine, and 1 mM sodium pyruvate, supplemented with 10% FBS, 100 U mL^−1^ penicillin, and 100 µg mL^−1^ streptomycin. The test for Mycoplasma yielded a negative result. Cells were incubated in an incubator with > 90% humidity at 37°C and 5% CO_2_. Transient transfections were performed using jetOPTIMUS (101000006, Polyplus, France) or LipoJet (SL100468, SignaGen, USA) according to the manufacturer's protocol.

### 5.6 Luciferase Assay

To measure the activity of the Gal4/UAS and QF/QUAS transcription systems, cells and zebrafish embryos were transfected or injected with different plasmids. In the cell experiment, each well of a 96‐well plate was transfected with 50 ng of either QF or Gal4 transactivator plasmid, along with 50 ng of 5×QUAS‐Luc2 (a Firefly luciferase derivative) or 14×UAS‐Luc2 plasmid, and 10 ng of SV40‐RL (Renilla luciferase). This was performed in triplicate. After 24 h, cell lysis was carried out using 100 µl of 1× passive lysis buffer. In zebrafish experiments, one‐cell‐stage embryos were co‐injected with 30 pg of *ubb*:QF2, *ubb*:QFvpr, *ubb*:KalTA4, or *ubb*:Gal4vpr plasmid, 30 pg of 5×QUAS‐Luc2 or 14×UAS‐Luc2 plasmid, and 10 pg of Renilla luciferase mRNA. At 6 hpf, 20–30 embryos were pooled per condition and placed in a 1.5 mL microtube. These embryos were then washed with 1 mL of phosphate‐buffered saline (PBS) and centrifuged for 30 s. The supernatant was carefully removed by aspiration. Lysates were prepared by adding 3 µl of 1× passive lysis buffer to each embryo. The mixture was homogenized using a plastic pestle and subsequently centrifuged for 1 min. 20 µl of lysates from either cells or embryos were transferred into a well of a white opaque 96‐well plate. The Dual‐Luciferase Reporter Assay system (E1910, Promega, USA) was used to measure the firefly and Renilla luciferase activities using a Synergy HTX plate reader from BioTek (USA). The Firefly luciferase activity was then normalized by the Renilla luciferase activity.

### 5.7 RNA Extraction and qRT‐PCR

For real‐time quantitative PCR (qPCR), approximately 20–30 zebrafish embryos/larvae were collected at the specified time point after heat shock treatment. For the cell experiment, 333 ng of CasRx or Cas13X plasmid, along with 666 ng of gRNA plasmid, was transfected into a single well of a 12‐well plate. Total RNA was then extracted using TRIzol (15596026, Invitrogen, USA) or RNAiso Plus (91085, Takara Bio, Japan) following the manufacturer's protocols. Subsequently, 500 ng of total RNA was used for cDNA synthesis using random hexamers and HiScript III RT SuperMix with gDNA wiper (R323‐01, Vazyme, China). The qPCR amplification was carried out using the CFX Connect Real‐Time PCR system (Bio‐Rad, USA), in conjunction with the 2× Taq Pro Universal SYBR qPCR Master Mix (Q712‐03, Vazyme, China). The levels of gene expression were determined utilizing the ΔΔCt method, with normalization against actin. Every sample was assessed in triplicate. The detailed list of primers employed for qPCR can be found in Table S2.

### 5.8 RNA‐seq Libraries and Analysis

To analyze the transcriptome, RNA samples that had been collected for qRT‐PCR were used to construct RNA libraries following standard procedures. These libraries were sequenced on the Illumina NovaSeq platform, generating 150 bp paired‐end reads. Subsequently, Trimmomatic version 0.39 was used to remove adapter sequences and low‐quality reads. The resulting sequencing reads were aligned to the *Danio rerio* genome version GRCz11 (danRer11) using STAR version 2.7.10b. Transcripts were quantified using Salmon v1.10.1 to determine transcript abundance in terms of TPM (Transcripts per Million).

### 5.9 Genomic DNA Extraction and qPCR for Copy Number Analysis

Genomic DNA was extracted from individual larvae at 4 dpf, and transgene copy numbers were determined by qPCR. DNA levels of QFvpr, CasRx, and dCas9vpr were quantified and normalized to *gapdh* genomic DNA. *actb1* copy number was measured as an internal control to validate the assay. gRNA copy numbers were normalized to wild‐type fish, which contain two endogenous copies (the AV‐qPCR‐F/R primer pair has two endogenous binding sites). Primer sequences are listed in Supplementary Table S2.

### 5.10 Video Recording

To record videos of zebrafish larvae to assess their locomotor activities and sensory responses, two larvae were placed in a single well of a 96‐well plate with 350 µl of E3 medium. The basal locomotor activity of juveniles was assessed by placing one fish per well in a 24‐well plate. Adult fish were individually placed in a four‐chamber transparent box (13 × 10 × 4 cm, one fish per chamber). The background was illuminated with an infrared LED (peak wavelength of 880 nm). Videos were captured with a Grasshopper3 USB3 camera (GS3‐U3‐41C6M‐C, Teledyne FLIR, USA) at 15 frames per second (fps) and a resolution of 2048 × 2048 for light stimulation assays, or at 120 fps for the vibrational startle response assay (VSRA). Refer to the VSRA section below for details. The camera was mounted on a telecentric lens (SO‐TEL‐H62C‐0680, Shanghai OPTICS, China) to ensure a constant and non‐angular field of view. An infrared pass filter (#89‐834, Edmund, USA) was used to block visible light and prevent any interference with the photic stimuli. Video recording and real‐time tracking of the animals were performed using MARGO [94]. The analysis of movement distance and the generation of plots were carried out using custom‐written R scripts for this purpose.

### 5.11 Light Stimulation Assay

At 7 dpf, larvae were transferred from dishes to 96‐well plates as described above and allowed to acclimatize for approximately 5 h before the behavioral assay. Two custom‐built LED light modules delivered multicolored light for the assay. The procedure for the light assay was as follows: three dark‐light cycles, each including 3‐min darkness and 3‐min white light; four dark‐strobe cycles, each consisting of 1‐min darkness and 1‐min white strobe light; three purple‐blue cycles, each with 1.5‐min purple light and 1.5‐min blue light. The white strobe light alternated between 100‐ms on and 100‐ms off (Figure S9E). Activity was quantified by averaging the mean values for each period. For the dark‐strobe cycles, mean values were calculated during the 31–60 s interval of each period, excluding the first cycle due to less reliable responses. Each well was considered a separate biological repeat.

### 5.12 Vibrational Startle Response Assay

The vibrational startle response assay (VSRA) utilized an automated system to deliver vibrational stimuli through two custom‐built tapping devices. The VSRA consisted of six vibrational stimuli at the highest intensity (intensity level 8), with a 5‐s interval between each stimulus (Figure S6E). Videos were recorded at 120 fps, and the VSR was analyzed for each well by measuring the maximum speed within the 5‐frame period following the stimulus.

### 5.13 Birefringence and Touch‐Evoked Escape Response Assay

Birefringence, as seen in structures like muscle sarcomeres, is the optical property where the refractive index changes with the polarization and direction of light. To measure birefringence, 4‐dpf larvae were anesthetized with 0.02% tricaine and carefully positioned in a stereomicroscope (SOPTOP SZ, China) between two polarizing filters. One filter was attached to the objective lens, while the other was placed on the stage plate. Birefringence was captured upon adjusting one of the filters to maximize brightness, and subsequently, a brightfield image was taken by a digital microscope camera (SOPTOP, China). The intensity of the pixels in the trunk region was measured using Fiji software.

To measure touch responses, zebrafish larvae were placed in a 10 cm Petri dish containing E3 medium. Tactile stimulation was applied to the larval tail using a polished 25 G needle. Images were captured at 50 fps using FlyCap2 software and a Grasshopper3 USB3 camera (Teledyne FLIR, USA), equipped with an infrared filter and a high‐resolution 16 mm focal length lens (#86–571, Edmund, USA). The resulting AVI video file was analyzed in Fiji to measure the distance traveled during the touch‐evoked movement. Typically, 10 frames were used for analysis, indicating that the touch‐evoked response lasted approximately 0.2 s.

### 5.14 Immunofluorescence Staining

The procedure of immunofluorescence staining largely follows the published protocol [95] with minor modifications. In brief, 0.003% propylthiouracil (PTU) was added to the E3 medium before 24 hpf to inhibit pigmentation. The 6‐dpf zebrafish larvae were fixed in 2% formaldehyde in PBS overnight at 4°C and washed twice for 10 min in PBS. Subsequently, larvae were transferred to a 60 mm dish, where the skin was removed by tweezers, followed by dehydration in methanol for 10 min and further incubation at −20°C in methanol for at least 2 h. The larvae were then treated with 3% H_2_O_2_ diluted in methanol for at least 1 h. Subsequent rehydration was achieved by successive incubations in 75%, 50%, and 25% methanol for 5 min each, followed by two 5‐min PBST (PBS with 0.1% Tween 20) washes. Larvae were then treated with 0.5 mg mL^−1^ collagenase (S10053, Shanghai Yuanye Bio‐Technology, China) for 20 min. After two PBST washes, larvae were refixed with 2% formaldehyde for 10 min, washed twice with PBST, permeabilized in PT (PBST with 1% Triton X‐100) for 1 h, preincubated in blocking buffer (5% goat serum and 2 mg mL^−1^ bovine serum albumin (BSA) in PBST), and incubated with the primary antibodies, a 1:200 dilution of rabbit polyclonal anti‐MYH4 (A15293, ABclonal, China) or mouse monoclonal anti‐Vinculin (66305‐1‐Ig, Proteintech, China) in blocking buffer, at 4°C overnight. After five 20‐min PT washes, the secondary antibody conjugated with Alexa Fluor 594 (1:1000) (ABclonal, China) was applied and incubated overnight at 4°C, followed by five 20‐min PT washes. Finally, the larvae were mounted in 70% glycerol in PBS onto slides and oriented to obtain a lateral view of the trunk before being covered with a coverslip. We looked for abnormal myocytes in the trunk between the eighth and 18^th^ somite. Images were acquired via z‐stacks of the representative areas.

### 5.15 Western Blot Analysis

Embryos or larvae, with or without treatment, were collected, washed with ice‐cold PBS, and lysed in RIPA buffer (50 mM Tris‐HCl pH 7.4, 150 mM NaCl, 1% Triton X‐100, 1 mM EDTA, 5% glycerol) supplemented with a protease inhibitor cocktail. Lysates were briefly sonicated, centrifuged, and protein concentration was determined by BCA assay. Samples were mixed with SDS sample buffer, boiled, separated by 10%–15% SDS‐PAGE, and transferred to PVDF membranes. Membranes were blocked with 5% milk in Tris‐buffered saline containing 0.05% Tween‐20 (TBST), overnight at 4°C with primary antibodies (1:500 anti‐GPX4, ET1706‐45, HUABIO; 1:5000 anti‐phospho‐p38, #9211, Cell Signaling Technology; 1:2000 anti‐β‐Tubulin, AC021, ABclonal), followed by HRP‐conjugated secondary antibody incubation. After washing, membranes were developed using Clarity Western ECL (1705060, Bio‐Rad).

### 5.16 Drug Treatment for Stress and Immune Response

Larvae and juveniles were treated in 24‐well plates, and adults were treated in 50 mL beakers. Apoptosis was induced using camptothecin (CPT; HY‐16560, MedChemExpress): 4 dpf larvae were treated with 2 µM CPT for 5 h, while juvenile and adult zebrafish were treated with 5 µM and 6 uM CPT, respectively, for 24 h. ER stress was induced with tunicamycin (TM; T101151, Aladdin): 3 dpf larvae, juveniles, and adults were treated with 2 µg/ml for 24 h, 4 µg/ml for 24 h, and 4 µg/ml for 8 h, respectively. ROS stress was induced using tert‐butyl hydroperoxide (tBHP; B106035, Aladdin): 4 dpf larvae were treated with 0.8 mM for 4 h, juveniles with 0.6 mM for 5 h, and adults with 0.6 mM for 4 h. Immune responses were induced by lipopolysaccharide (LPS) injection: 4 dpf larvae or juveniles were injected with 2 nL or 10 nL of 3 mg/ml LPS (L9143, Sigma), respectively, and analyzed after 24 h or 6 h; adults were injected with 20 µl of 1.5 mg/ml LPS (L2630, Sigma) and dissected after 12 h.

### 5.17 Terminal Deoxynucleotidyl Transferase (TdT) dig‐dUTP Nick End Labeling (TUNEL)

For 4 dpf larvae, fixation, skin removal, dehydration, rehydration, and collagenase digestion were performed as described for immunofluorescence staining. Juveniles and dissected adult organs were fixed overnight at 4°C in 4% formaldehyde, equilibrated in 30% sucrose at room temperature, and embedded in OCT reagent (G6059, Servicebio). Cryosections were cut at 20 µm thickness. TUNEL assays were performed using the One‐step TUNEL In Situ Apoptosis Kit (Red, Elab Fluor 594) (E‐CK‐A322, Elabscience) according to the manufacturer's instructions. Briefly, cryosections were post‐fixed and permeabilized with proteinase K. Both whole‐mount larvae and cryosections were equilibrated in TdT equilibration buffer, incubated with the TUNEL working solution, and counterstained with DAPI.

### 5.18 Hybridization Chain Reaction (HCR) RNA‐FISH

Whole‐mount staining of zebrafish embryos using HCR was performed as previously described with minor modifications [96, 97]. Briefly, embryos were fixed overnight at 4°C in 4% paraformaldehyde (PFA) in PBS, then washed with PBST. Samples were dehydrated through a graded methanol series and stored in 100% MeOH at −20°C. For rehydration, embryos were washed sequentially with 75%, 50%, and 25% MeOH in PBS, followed by PBST. Decolorization was carried out using 3% H_2_O_2_ + 0.5% KOH for 15 min, followed by permeabilization with 0.5% Triton X‐100 and treatment with 10 µg mL^−1^ proteinase K for 30 min. Embryos were post‐fixed in 4% PFA for 20 min and washed in PBST and 5× SSCT (5× SSC, 0.1% Tween‐20). Prehybridization was performed at 37°C for 30 min in hybridization buffer (30% formamide, 5× SSC, 9 mM citric acid pH 6.0, 0.1% Tween‐20, 50 µg mL^−1^ heparin, 1× Denhardt's solution, and 10% dextran sulfate). Probe hybridization was carried out by incubating embryos in HCR probe solution (10 pmol per probe) for 12–16 h at 37°C. Excess probe was removed by four 15‐min washes in prewarmed wash buffer (30% formamide, 5× SSC, 9 mM citric acid pH 6.0, 0.1% Tween‐20, 50 µg mL^−1^ heparin), followed by three 10‐min washes in 5× SSCT. Amplification was initiated by incubating embryos in amplification buffer (5× SSCT, 10% dextran sulfate) for 10 min. Snap‐cooled hairpins h1 and h2 (30 pmol each) were added to fresh amplification buffer and applied to samples for 2 h at room temperature in the dark. After amplification, excess hairpins were removed with three washes in 5× SSCT. Embryos were stored at 4°C until imaging.

### 5.19 Image Acquisition

Zebrafish embryos or larvae of different ages were anesthetized with 0.02% tricaine and carefully placed under a Zeiss Axio Zoom V16 fluorescence stereo microscope equipped with a PlanNeoFluar Z 1× objective and an Axiocam 506 mono camera. For images of immunofluorescence and HCR staining, z‐stack images of larvae were captured using a Nikon Ti2‐E coupled with an X‐Light V3 spinning disk (CrestOptics, Italy), an LDI‐7 laser system (89 North, USA), and a Prime 95B 25 mm camera (Teledyne Photometrics, USA), with a CFI Plan Apochromat Lambda 100× objective (N.A. 1.45) for immunofluorescence and a CFI Plan Fluor 10× objective (N.A. 0.30) for HCR staining and TUNEL. Images were processed using Fiji software.

### 5.20 Statistical Analysis

All results are expressed as mean ± s.e.m. from at least three independent experiments, unless otherwise stated. Statistical significance was determined using an unpaired two‐tailed Student's *t*‐test, unless otherwise stated. *P* < 0.05 was considered statistically significant. All statistical analyses were conducted using GraphPad Prism 9.5.0.

## Conflicts of Interest

The authors declare no conflicts of interest.

## Supporting information




**Supporting File**: advs74413‐sup‐0001‐SuppMat.pdf

**Supporting File**: advs74413‐sup‐0002‐MovieS1.mp4

**Supporting File**: advs74413‐sup‐0002‐MovieS2.mp4

## Data Availability

The data that support the findings of this study are openly available in [GEO] at [https://www.ncbi.nlm.nih.gov/geo/query/acc.cgi?acc═GSE268371], reference number [268371].
