# Supplementary material for: Versatile CRISPR‐Cas Tools for Gene Regulation in Zebrafish via an Enhanced Q Binary System
Source: Adv Sci (Weinh). 2026 Feb 11;13(23):e11485. doi: 10.1002/advs.202511485 (PMC13104143; doi:10.1002/advs.202511485)
Supplement: Supplementary file 1 — Supporting File: advs74413‐sup‐0001‐SuppMat.pdf [file ADVS-13-e11485-s003.pdf]

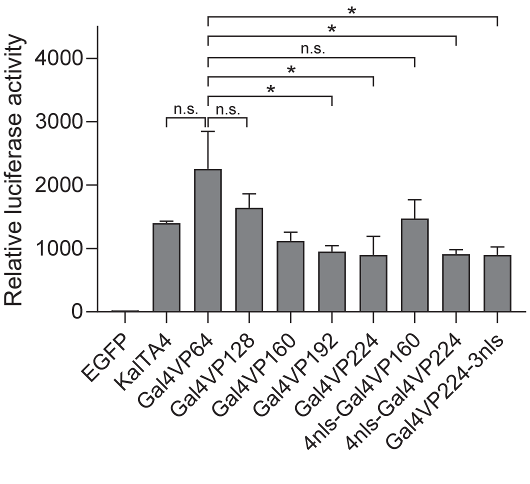

Supplementary Figure 1. Increasing the number of VP16 repeats or nuclear localization signal (NLS) in the Gal4 transactivator does not enhance gene activation. The Gal4 constructs, containing the indicated activation domain and NLS repeats, were co-transfected along with the 14×UAS-Luc2 luciferase reporter into HEK293T cells. Relative luciferase activity was determined as described in the methods. $n = 3$. Data are shown as mean ± s.e.m. Statistical analyses were performed using one-way ANOVA followed by Tukey's multiple comparisons test. * $P < 0.05$. n.s., not significant.

A

Zebrafish lines	Germline transmission
ubb:KalTA4-CK	0 % (0/98)
ubb:QFvpr-CK	2.1 % (1/47)

B

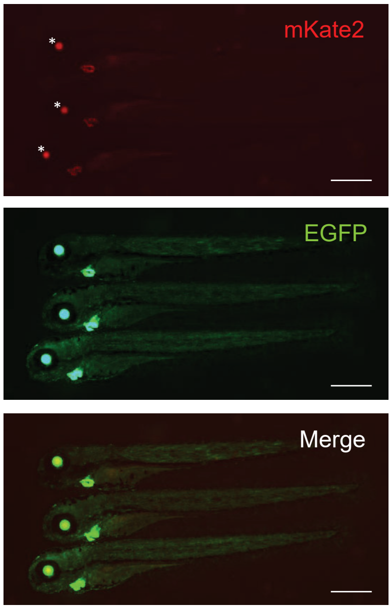

C

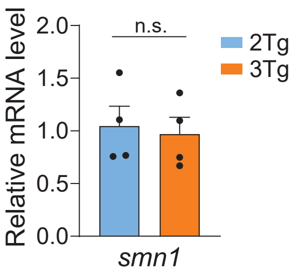

Supplementary Figure 2. QFvpr can be ubiquitously expressed, although the expression is relatively weak. (**A**) Summary of germline transmission rate of *ubb*:KalTA4-CK and *ubb*:QFvpr-CK zebrafish lines. (**B**) Representative fluorescence images of 3-dpf zebrafish embryos from 3Tg zebrafish Tg(*ubb*:QFvpr-CK; 5×*QUAS*:CasRx-2A-EGFP-CG; *zU6*:*smn1*-AV). The red fluorescence in the heart is generated by mKate2 under the control of the cardiac myosin light chain 2 (*cmlc2*) promoter, confirming the integration of *ubb*:QFvpr. Similarly, GFP fluorescence in the heart corroborates the integration of 5×*QUAS*:CasRx-2A-EGFP. GFP fluorescence in the eyes validate *zU6*:*smn1*-AV integration. CK, *cmlc2*:mKate2; CG, *cmlc2*:EGFP; AV, *α-crystallin*:Venus. The red fluorescence in eyes, marked by asterisks, is attributed to bleed-through from Venus. Scale bar, 500 µm. (**C**) The qPCR assays were conducted for *smn1*. $n = 4$. Data are presented as mean ± s.e.m. P values are derived from unpaired two-tailed t-test. n.s., not significant.

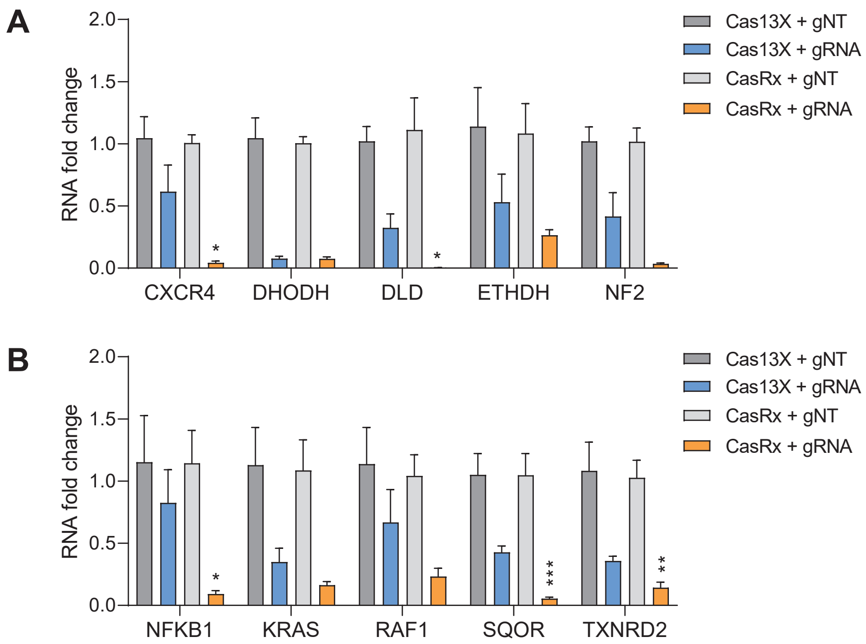

Supplementary Figure 3. CasRx exhibits greater knockdown efficiency compared to Cas13X. Plasmids of Cas13X or CasRx, along with their corresponding gRNAs targeting indicated genes, were co-transfected into HEK293T cells. (**A**) Quantitative plot of RNA fold changes for five endogenous genes (CXCR4, DHODH, DLD, ETHDH, and NF2) (**B**) Quantitative plot of RNA fold changes for additional five endogenous genes (NFKB1, KRAS, RAF1, SQOR, and TXNRD2) $n = 4$ (A, B). Data are presented as mean ± s.e.m. P values are derived from unpaired two-tailed t-test comparing Cas13X + gRNA and CasRx + gRNA. P values of comparisons that reached statistical significance are indicated. * $P < 0.05$; ** $P < 0.01$; *** $P < 0.001$. gNT, non-targeting guide RNA; gRNA, guide RNA.

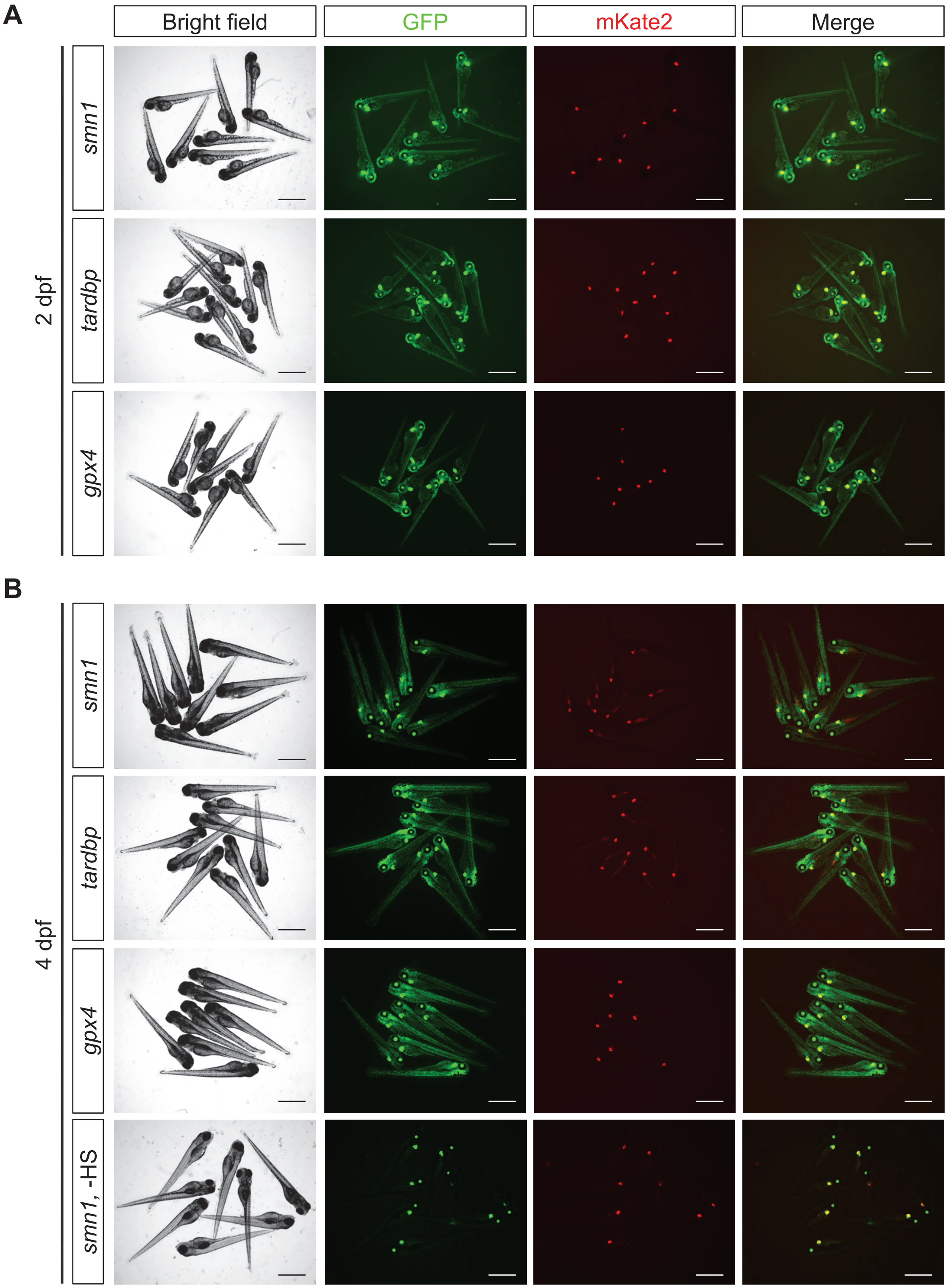

Supplementary Figure 4. Representative fluorescence images of 3Tg zebrafish embryos resulting from crosses between 2Tg zebrafish Tg(*hsp70l*:QFvpr-CK; 5×*QUAS*:CasRx-2A-EGFP-CG) and transgenic zebrafish expressing different gRNAs targeting *smn1*, *tardbp* covering both *tardbp* and *tardbpl*, or *gpx4* covering both *gpx4a* and *gpx4b*. The embryos were subjected to daily heat treatment at 2 and 3 dpf, and fluorescence images of EGFP were captured at 2 dpf (**A**) or 4 dpf (**B**). Heat shock-free (-HS) embryos derived from 3Tg *smn1* transgenic embryos was included as a negative control (**B**). Scale bar, 1 mm.

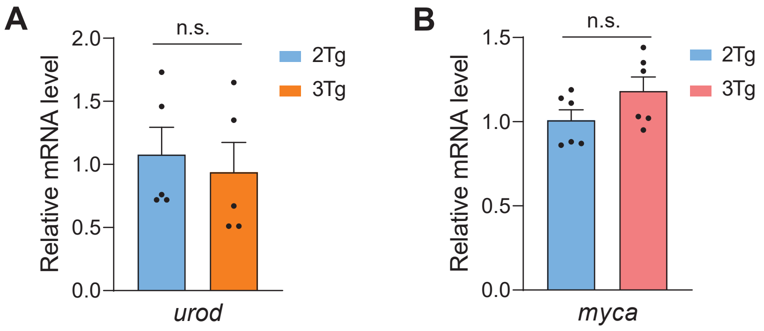

Supplementary Figure 5. Examples of no significant change in mRNA levels by CRISPR-Q_{KD} (**A**) or CRISPR-Qa (**B**). (**A**) For knockdown, 2Tg zebrafish Tg(*hsp70l*:QFvpr-CK; 5×*QUAS*:CasRx-2A-EGFP-CG) were crossed with transgenic zebrafish expressing gRNA targeting *urod*. (**B**) For gene activation, 2Tg zebrafish Tg(*hsp70l*:QFvpr-CK; 5×*QUAS*:sfGFP-dCas9vpr-AK) were crossed with transgenic zebrafish expressing 3×sgRNAs targeting three sites of promoter in the *myca* gene. Embryos were subjected to heat treatment at 2 and 3 dpf and collected at 4 dpf. The qPCR was performed for *urod* (A, $n = 5$) and *myca* (B, $n = 6$). Data are presented as mean ± s.e.m. P values are derived from unpaired two-tailed t-test. n.s., not significant.

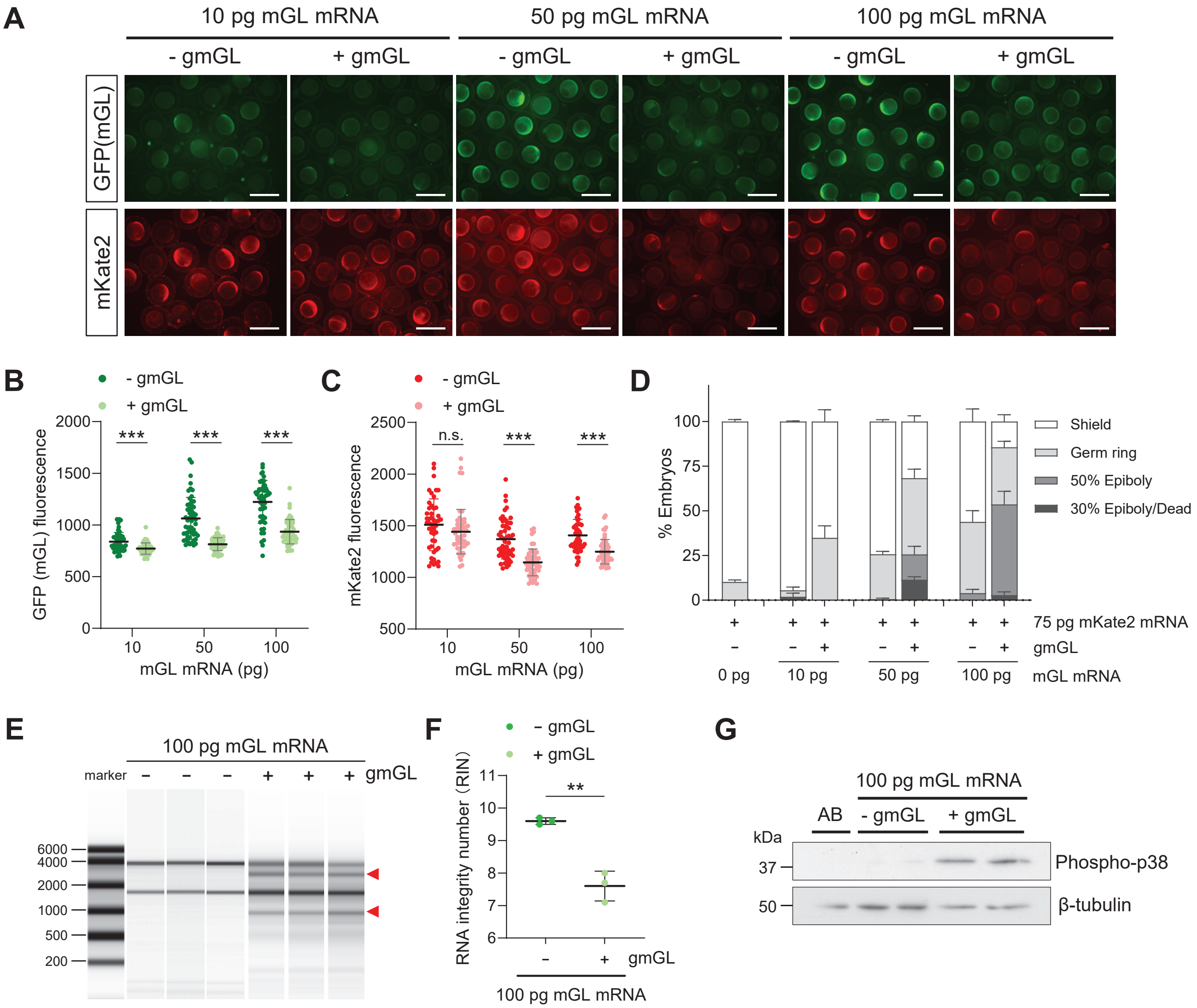

Supplementary Figure 6. CasRx exhibits collateral activity when targeting abundant ectopic mRNAs in zebrafish embryos. (**A**) Representative fluorescence images of embryos at 6 hpf injected with the indicated amounts of mGreenLantern (mGL) mRNA together with 75 pg mKate2 mRNA, 300 pg CasRx mRNA (mCasRx), and gRNAs targeting mGL (gmGL, a mixture of three gRNAs, 100 pg per gRNA). Scale bar, 1 mm. (**B**, **C**) Scatter plots show quantitative fluorescence intensities of mGL (**B**) or mKate2 (**C**) in embryos shown in (A). From left to right, n = 49, 54, 55, 54, 49, 54 (B, C). (**D**) Stacked bar plot shows developmental phenotypes (epiboly stages) at 6 hpf in embryos injected with mCasRx and the indicated materials. n = 3. Data are shown as mean ± s.e.m. (**E**) Bioanalyzer traces showing RNA integrity of samples purified at 6 hpf from embryos injected with 100 pg mGL mRNA and 300 pg CasRx mRNA (mCasRx), with or without gmGL (a mixture of three gRNAs; 300 pg per gRNA). (**F**) Dot plot of RNA integrity numbers (RINs) calculated from the samples shown in (E). n = 3. (**G**) Western blot analysis of phosphorylated p38 levels in 6-hpf uninjected AB embryos and embryos injected as described in (E). Data are presented as mean ± s.d (B, C, F). P values were determined using an unpaired two-tailed t-test. ** $P < 0.01$; *** $P < 0.001$; n.s., not significant.

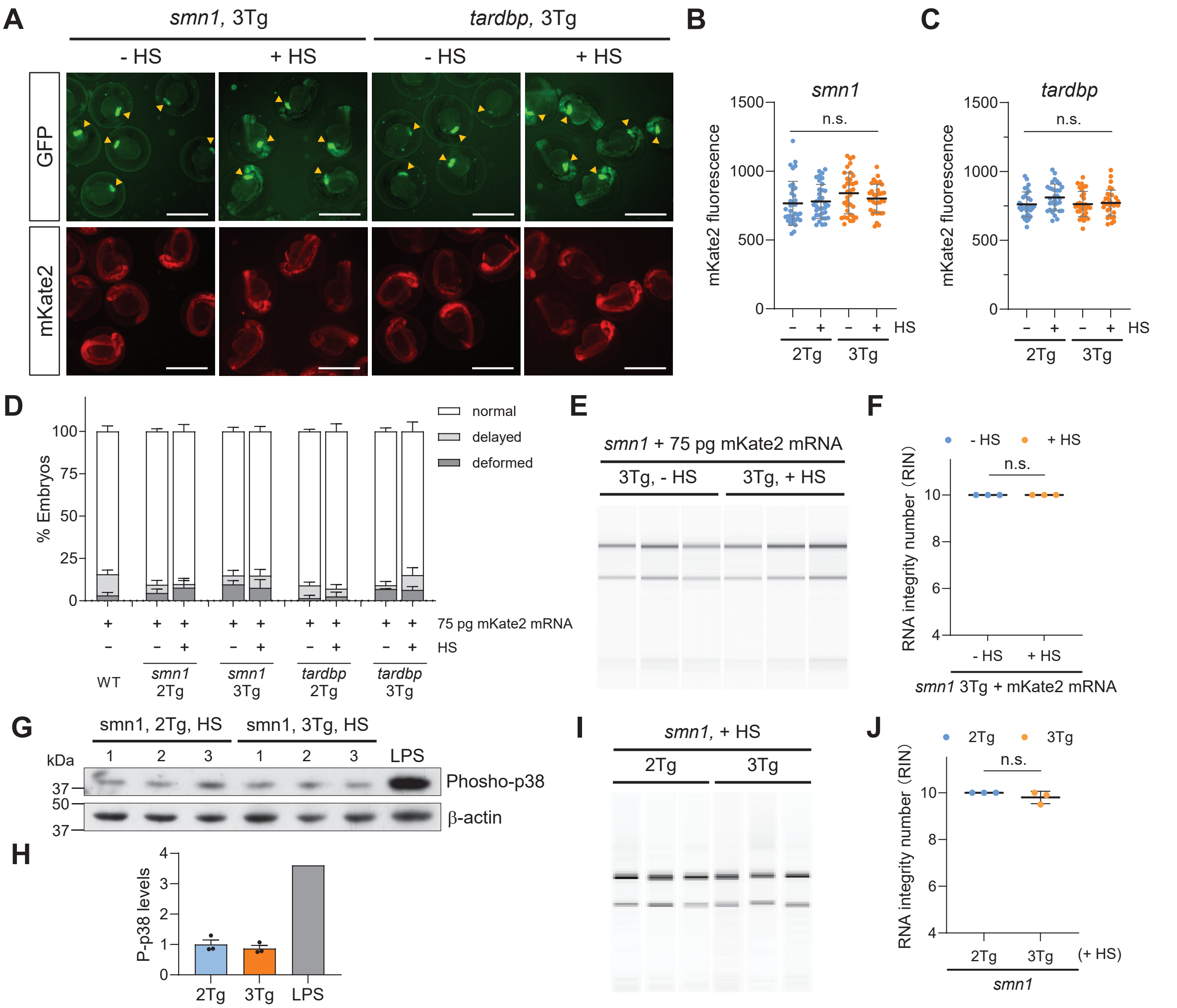

Supplementary Figure 7. CRISPR-Q$_{KD}$ does not induce detectable collateral effects when targeting endogenous transcripts. (**A**) Representative fluorescence images of *smn1* or *tardbp* 3Tg embryos at 30 hpf injected with 75 pg mKate2 mRNA, with or without heat shock (+ HS or - HS). Yellow arrowheads indicate transgene marker fluorescence in the heart. Scale bar, 1 mm. (**B**, **C**) Scatter plots show the quantitative mKate2 fluorescence intensities in 2Tg and 3Tg *smn1* (B) or *tardbp* (C) embryos. n = 35 (B); n = 32 (C). Data are shown as mean ± s.d. Statistical analyses were performed using one-way ANOVA followed by Tukey's multiple comparisons test. n.s., not significant. (**D**) Stacked barplot showing developmental phenotypes at 24 hpf in embryos injected with 75 pg mKate2 mRNA. n = 3. Data are shown as mean ± s.e.m. (**E**) Bioanalyzer traces showing RNA integrity of samples purified at 30 hpf from smn1 3Tg embryos injected as described in (A). (**F**) Dot plot of RNA integrity numbers (RINs) calculated from the samples shown in (E). (**G**) Western blot analysis of phosphorylated p38 protein levels in 4-day-old 2Tg and 3Tg *smn1* zebrafish larvae, and wild-type larvae injected with 3 mg/ml lipopolysaccharide (LPS) at 3 dpf. (**H**) Quantitation of Western blot data shown in (G). For heat shock, embryos were subjected to heat treatment at 37 °C for 45 min at 12 hpf (A-F) or daily heat treatment at 2 and 3 dpf (G, J). (**I**) Bioanalyzer traces showing RNA integrity of the samples shown in Figure 3E. (**J**) Dot plot of RNA integrity numbers (RINs) calculated from the samples in (I). n = 3. Data are presented as mean ± s.d. (F, J). P values were determined using an unpaired two-tailed t-test. n.s., not significant.

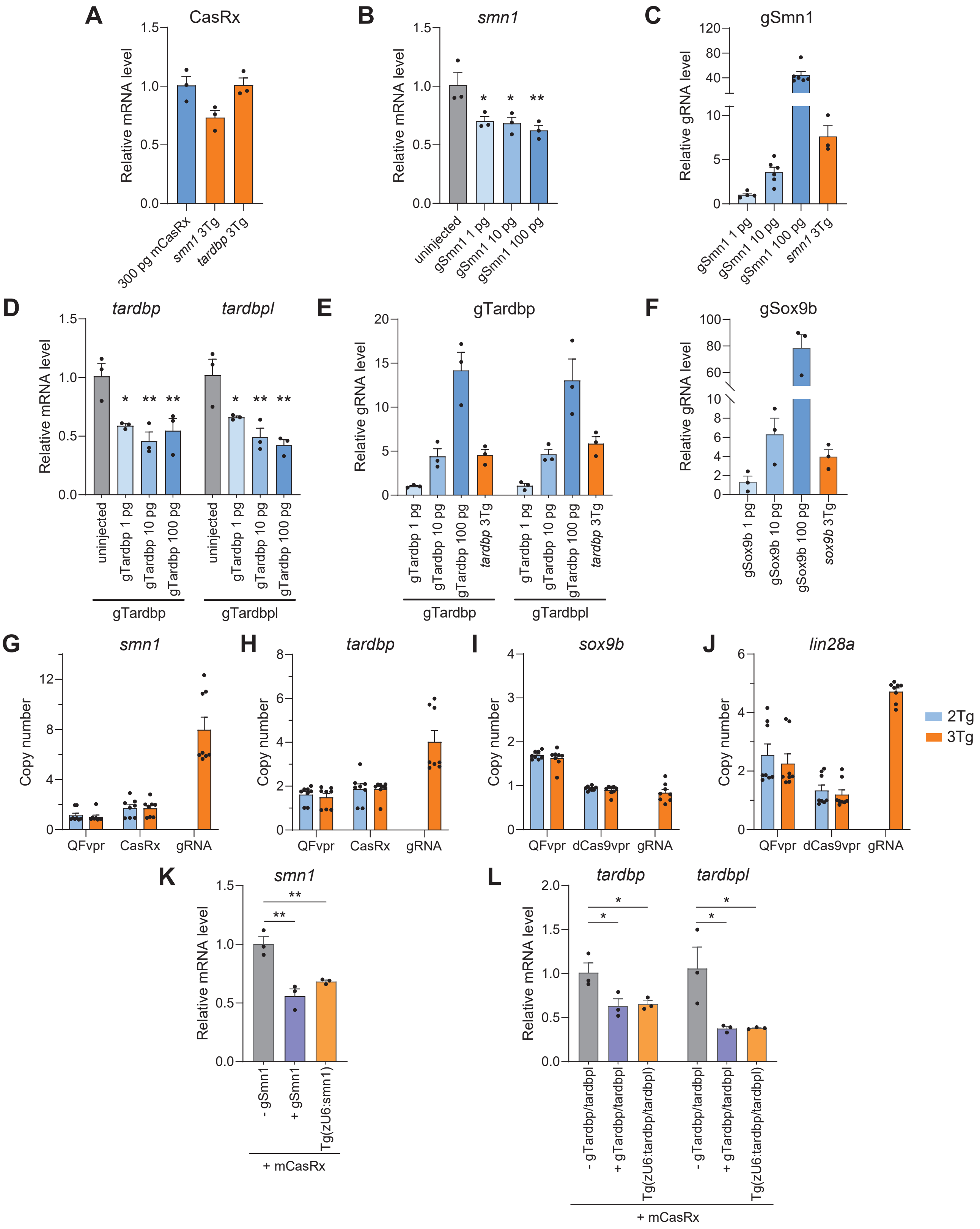

A
CasRx
Relative mRNA level
300 pg mCasRx
smn1 3Tg
tardbp 3Tg
B
smn1
uninjected
gSmn1 1 pg
gSmn1 10 pg
gSmn1 100 pg
C
gSmn1
Relative gRNA level
smn1 3Tg
D
tardbp
tardbpl
gTardbp 1 pg
gTardbp 10 pg
gTardbp 100 pg
gTardbp
gTardbpl
E
gTardbp
tardbp 3Tg
F
gSox9b
gSox9b 1 pg
gSox9b 10 pg
gSox9b 100 pg
sox9b 3Tg
G
smn1
Copy number
QFvpr
CasRx
gRNA
H
tardbp
I
sox9b
dCas9vpr
J
lin28a
2Tg
3Tg
K
smn1
- gSmn1
+ gSmn1
Tg(zU6:smn1)
+ mCasRx
L
- gTardbp/tardbpl
+ gTardbp/tardbpl
Tg(zU6:tardbp/tardbpl)

Supplementary Figure 8. Estimation of expression levels and copy numbers of CRISPR-Q effectors and gRNAs. (**A**-**F**) Bar plots show the indicated mRNA or gRNA expression levels in injected embryos at 24 hpf or in transgenic larvae at 4 dpf. For injected conditions, embryos were co-injected with 300 pg mCasRx and the indicated amounts of gRNAs. Transgenic embryos were subjected to daily heat treatment at 2 and 3 dpf. (**G**-**J**) Bar plots show the copy numbers of the indicated transgenes in 2Tg and 3Tg *smn1* (G), *tardbp* (H), *sox9b* (I), and *lin28a* (J) lines. (**K**, **L**) Bar plots show knockdown efficiency in 24 hpf embryos injected with 300 pg mCasRx and 150 pg of gRNAs targeting *smn1* (**K**) or *tardbp/tardbpl* (**L**), compared with transgenic gRNA conditions from Tg(zU6:*smn1*) or Tg(zU6:*tardbp*) embryos injected with 300 pg mCasRx. Each data point represents a single embryo in (K, L), while approximately 20 embryos were pooled for each qPCR sample in other experiments. $n = 3$ (A, B, D-F, K, L); $n = 4, 6, 6, 3$ (C); $n = 8$ (G-J). Data are shown as mean ± s.e.m. Statistical analyses in (B, D, K, L) were performed using one-way ANOVA followed by Dunnett's multiple comparisons test. The uninjected condition served as a control in (B, D). * $P < 0.05$; ** $P < 0.01$. gSmn1, gRNA targeting *smn1*; gTardbp, gRNAs targeting *tardbp* and *tardbpl*.

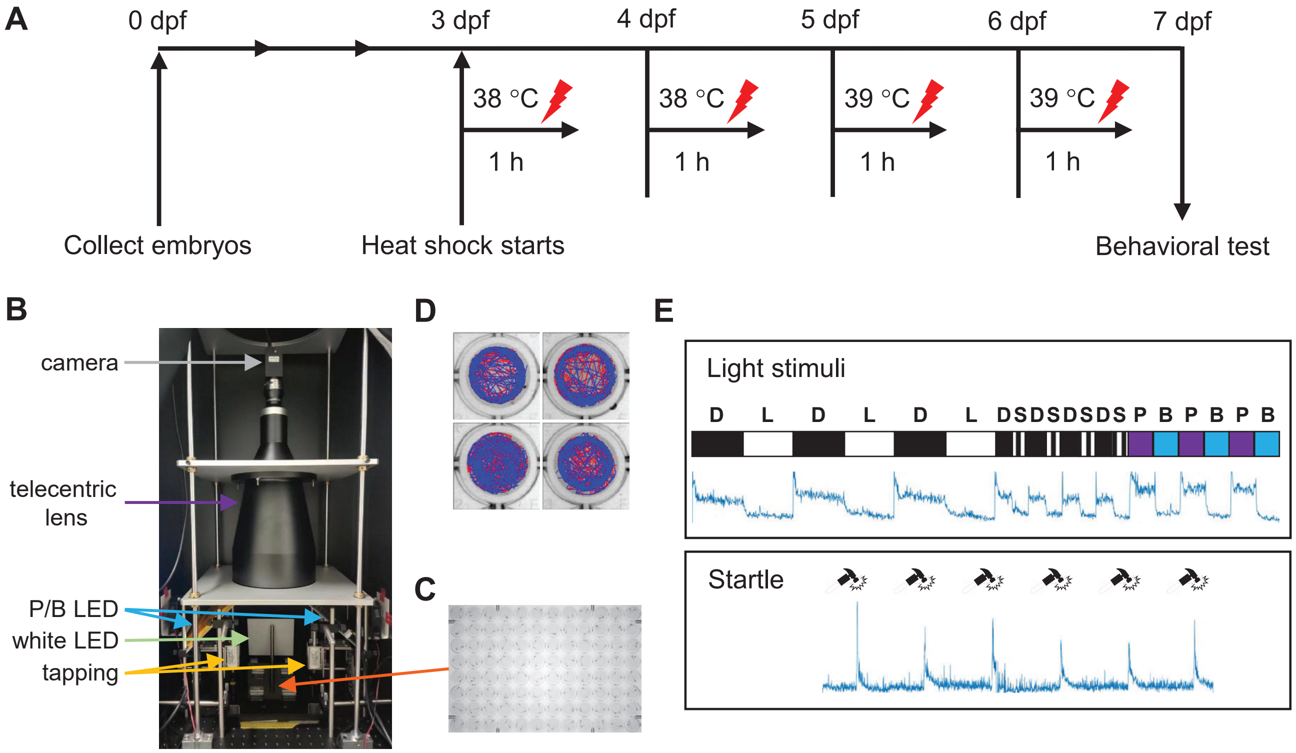

Supplementary Figure 9. Schematic design of zebrafish larval locomotion assay for responses to multiple stimuli. (**A**) Outline of the experimental procedure. To evaluate behavior at 7 dpf, larvae were exposed to a daily 1-h heat shock from 3 dpf to 6 dpf. Larvae were then transferred to a 96 well plate for behavioral assessment at 7 dpf. (**B**) Overview of the behavioral monitoring chamber: The locomotion of larvae in a 96-well plate was captured using a USB digital camera equipped with an infrared filter. A telecentric lens was attached to the camera to ensure a constant, undistorted view of each well across the entire plate. Light and vibrational stimuli were elicited using LED modules and tapping devices, respectively. (**C**) The 96-well plate was placed on a black, infrared-transparent acrylic board and received illumination from an infrared light source at the base. (**D**) An example of visualizing larval movement in wells. Tracks of two individual larvae were differentiated by blue and red colors. (**E**) Schematic representation of light and vibrational stimuli: corresponding locomotor responses in larvae. D, dark; L, white light; S, strobe light; P, purple light; B, blue light.

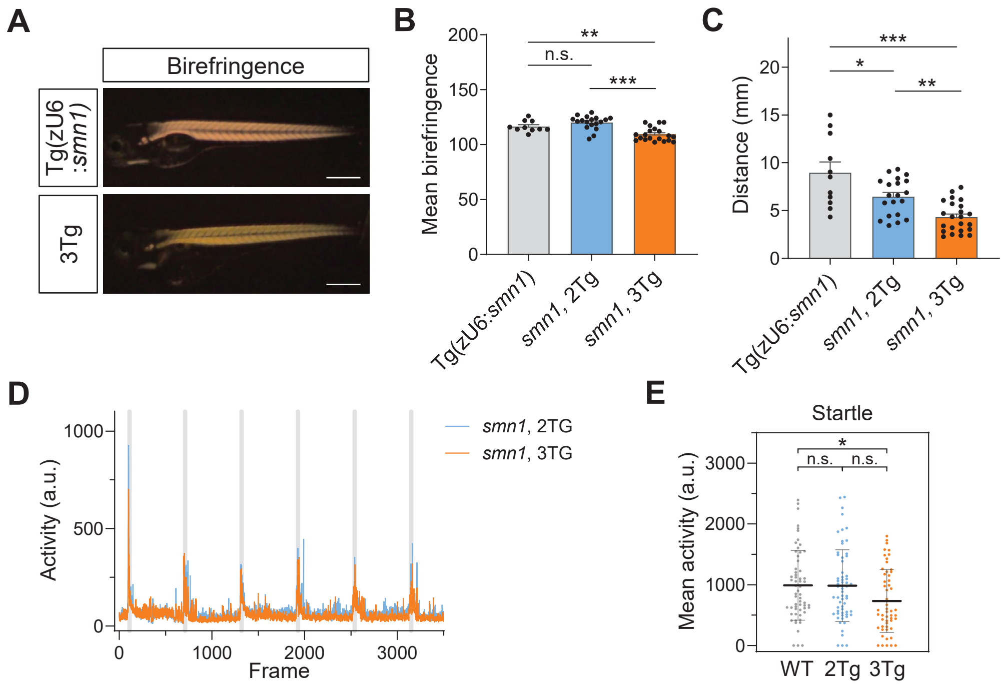

Supplementary Figure 10. Knockdown of *smn1* by CRISPR-Q_{KD} results in impairment in muscle but does not significantly alter vibrational startle responses. (**A**) Representative bright-field and birefringence images of larvae expressing only gSmn1 or 3Tg at 4 dpf. Scale bar, 500 µm. (**B**) The plot shows the quantitative analysis of birefringence of gSmn1, 2Tg, and 3Tg larvae as shown in A. n = 10 for gSmn1, n = 18 for 2Tg, and n = 20 for 3Tg. (**C**) Quantification of the touch-evoke response for gSmn1 (n = 11), 2Tg (n = 20), and 3Tg (n = 23) larvae. Statistical analyses (B and C) were performed using one-way ANOVA followed by Tukey's multiple comparisons test. * P < 0.05; ** P < 0.01; *** P < 0.001; n.s., not significant. (**D**, **E**) The average speed curves for all samples were analyzed at 7 dpf for locomotor activities. (D) and the quantitative scatter plot of each well (E) in the vibrational startle response assay (VSRA) are displayed. Each data point represents the six maximum speeds within the 5-frame period following six startle events for a single well (E). The grey bars in (D) indicate the vibrational tapping events. Speed curves for 96 wells are presented in Supplementary Figure 8D. n = 60 for WT, n = 62 for 2Tg, n = 54 for 3Tg (E) Data are shown as mean ± s.d. P values are derived from unpaired two-tailed t-test. n.s., not significant.

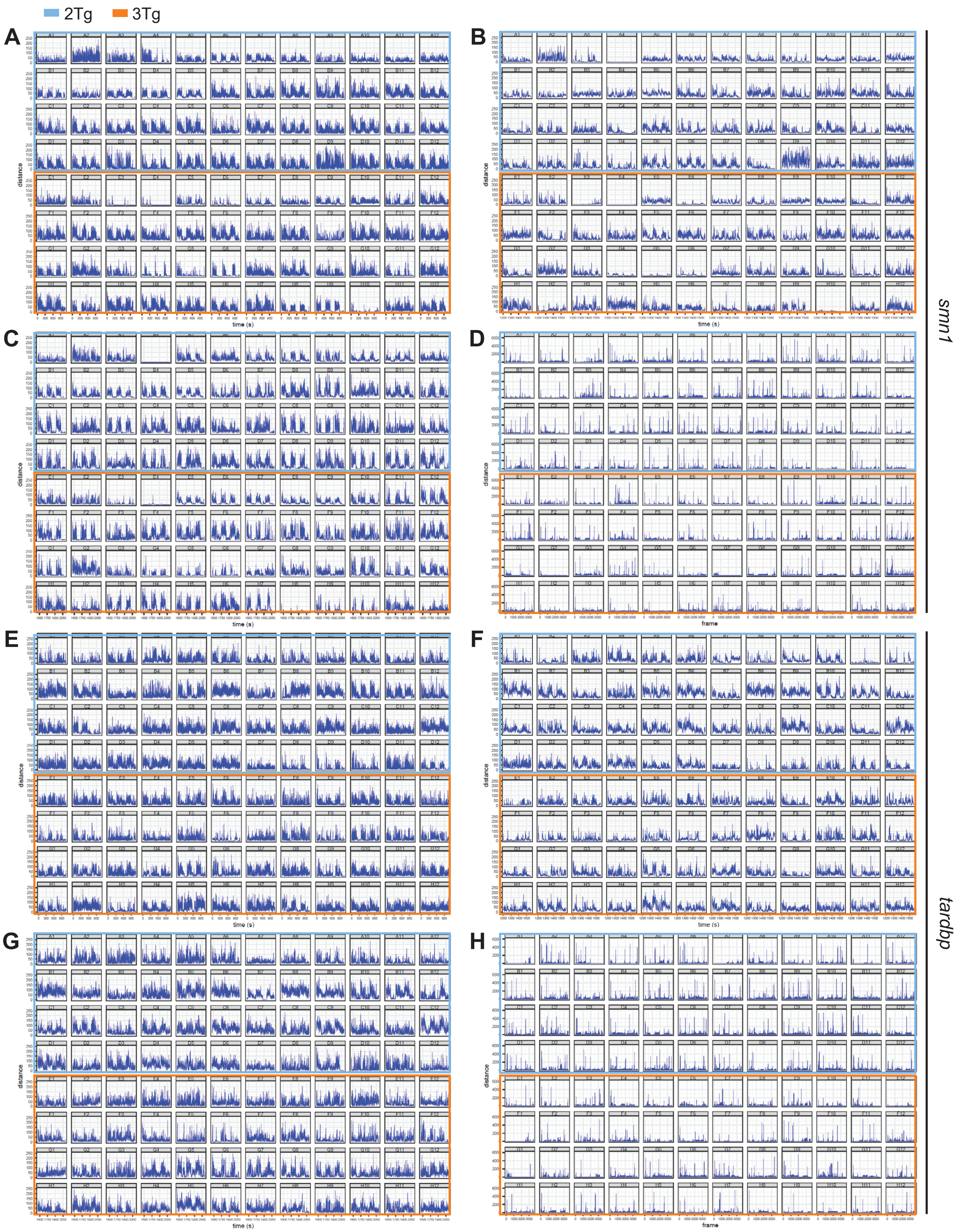

Supplementary Figure 11. Speed curves of individual wells in 96-well plates. Movement traces were generated from the combined activity of two larvae per well and analyzed at 7 dpf for locomotor activity. (**A-D**) Speed curves of 2Tg and 3Tg larvae for knockdown of *smn1* by CRISPR-Q_{KD} during the dark-light cycle (**A**), dark-strobe cycle (**B**), purple-blue cycle (**C**), and vibrational startle response (**D**). (**E-H**) Speed curves of 2Tg and 3Tg larvae for knockdown of *tardbp,* targeting both *tardbp* and *tardbpl,* by CRISPR-Q_{KD} during the dark-light cycle (**E**), dark-strobe cycle (**F**), purple-blue cycle (**G**), and vibrational startle response (**H**).

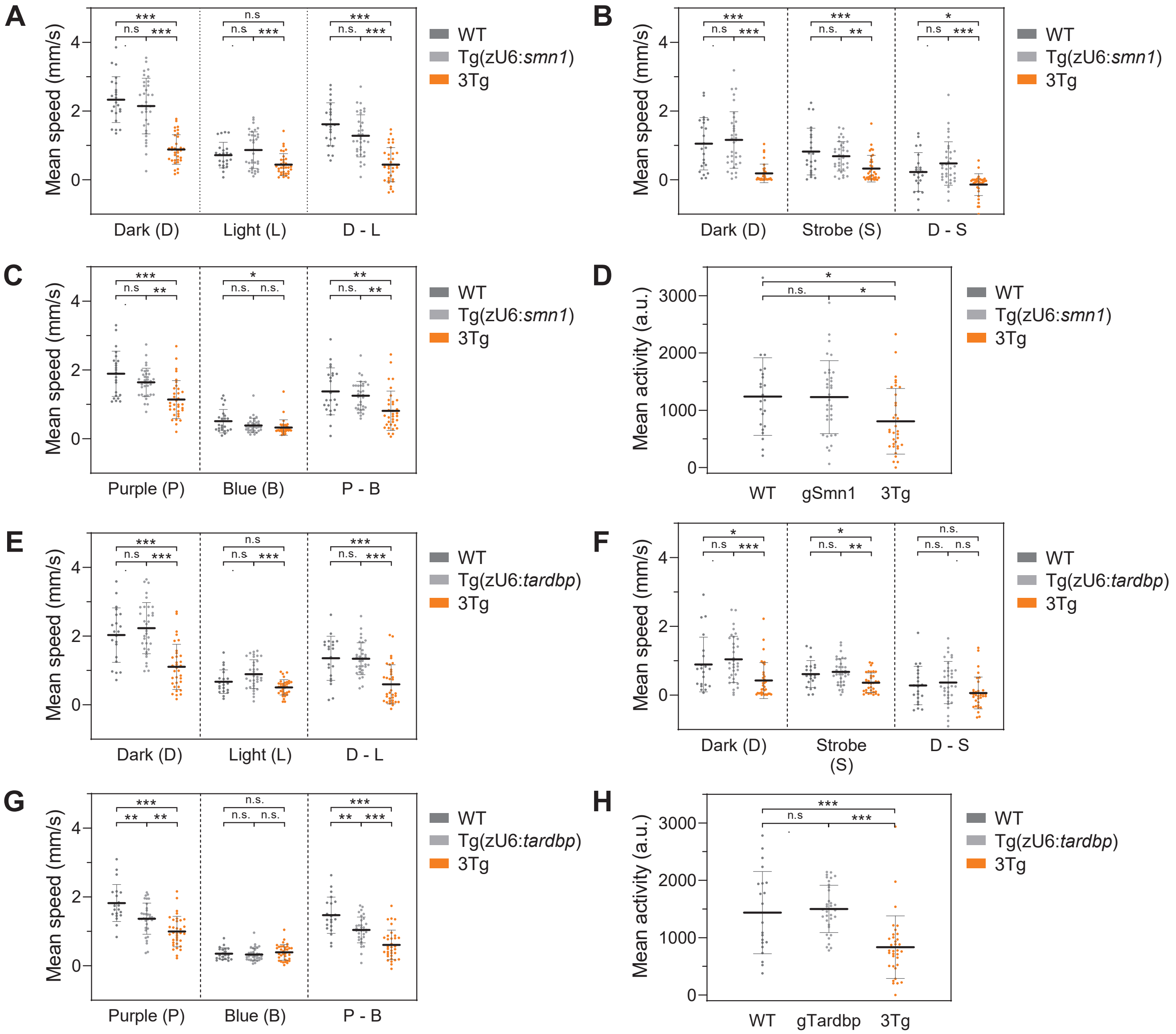

Supplementary Figure 12. Knockdown by CRISPR-Q_{KD} induces behavioral deficits. Wild-type larvae and transgenic larvae Tg(zU6:*smn1*) or Tg(zU6:*tardbp*) expressing gRNAs targeting *smn1* or both *tardbp* and *tardpl*, respectively, and 3Tg larvae expressing both Tg(*hsp70l*:QFvpr-CK; 5×*QUAS*:CasRx-2A-EGFP-CG) and the gRNA transgenes were subjected to daily heat treatment starting at 3 dpf and analyzed at 7 dpf for locomotor activities. Quantitative scatter plots are presented for four behavioral paradigms: dark-light cycle (**A**, **E**), dark-strobe cycle (**B**, **F**), purple-blue cycle (**C**, **G**), and the vibrational startle response assay (**D**, **H**). Each data point represents the average speed of two larvae per well during three defined cycles (A-C, E-G) or the six maximum speeds within the 5-frame window following six startle stimuli per well (D, H). D, dark; L, white light; S, strobe light; P, purple light; B, blue light. $n \geq 17$ (A-D); $n \geq 21$ (E-H). Data are shown as mean ± s.d. P values are derived from unpaired two-tailed t-test. * $P < 0.05$; ** $P < 0.01$; *** $P < 0.001$; n.s., not significant.

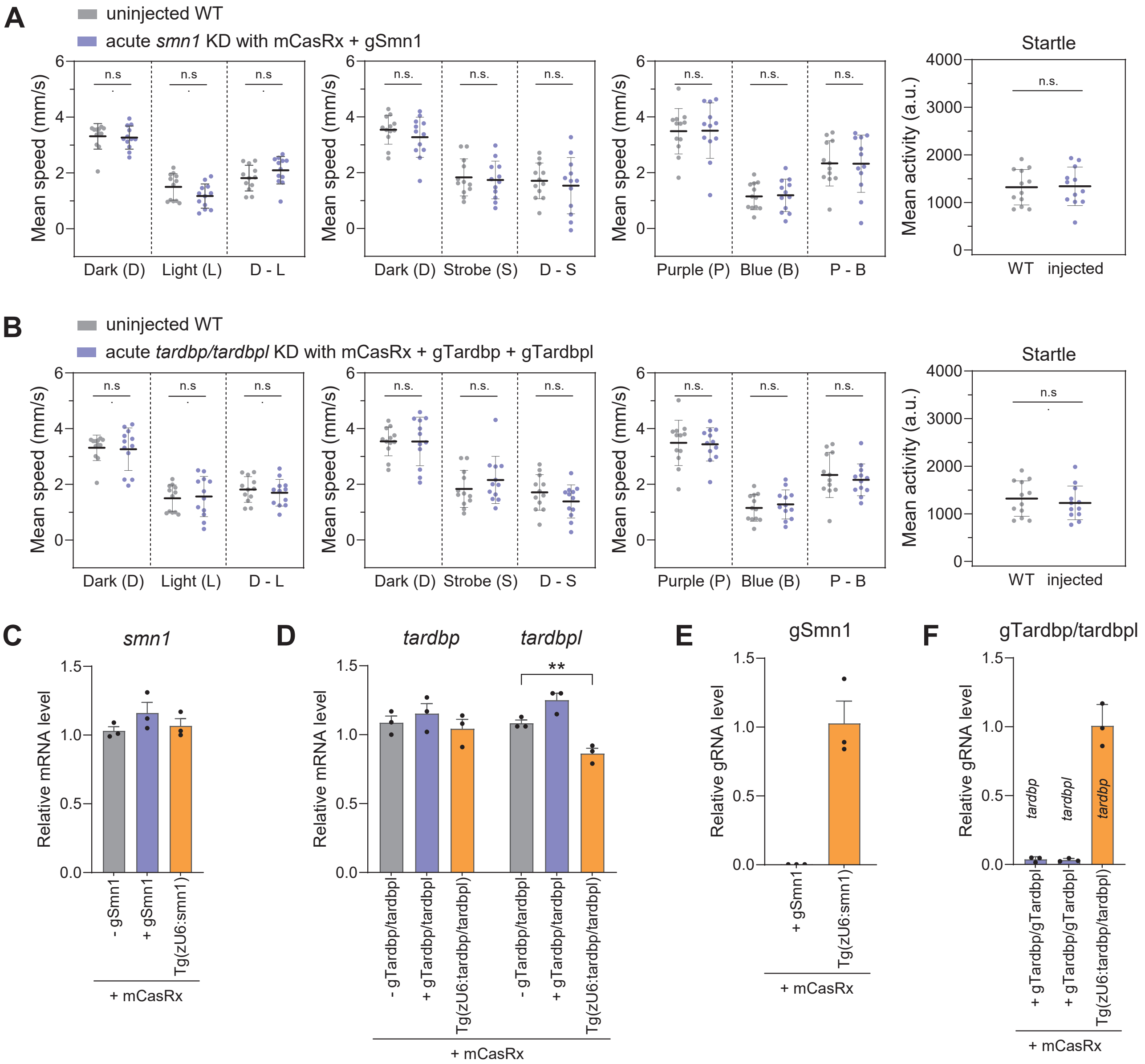

Supplementary Figure 13. Acute knockdown of *smn1* or *tardbp*/*tardbpl* by injection does not induce behavioral phenotypes or alter mRNA levels after 4 dpf. (**A**, **B**) Quantitative scatter plots are presented for four behavioral paradigms from left to right: dark-light cycle, dark-strobe cycle, purple-blue cycle, and the vibrational startle response assay. For acute *smn1* or *tardbp*/*tardbpl* KD, 300 pg CasRx mRNA (mCasRx) and 150 pg gRNA were co-injected into wild-type embryos. Larvae were subjected to behavioral assays at 7 dpf. $n = 12$. Data are shown as mean ± s.d. (**C**-**F**) Plots show KD efficiency (C, D) and gRNA levels (E, F) in larvae at 4 dpf. Wild-type embryos were co-injected with 300 pg mCasRx and 150 pg gRNAs, while transgenic embryos expressing gRNAs targeting *smn1* or *tardbp/tardbpl* were injected with 300 pg mCasRx. $n = 3$. Data are shown as mean ± s.e.m. *P* values are derived from unpaired two-tailed *t*-test. ** $P < 0.01$; n.s., not significant.

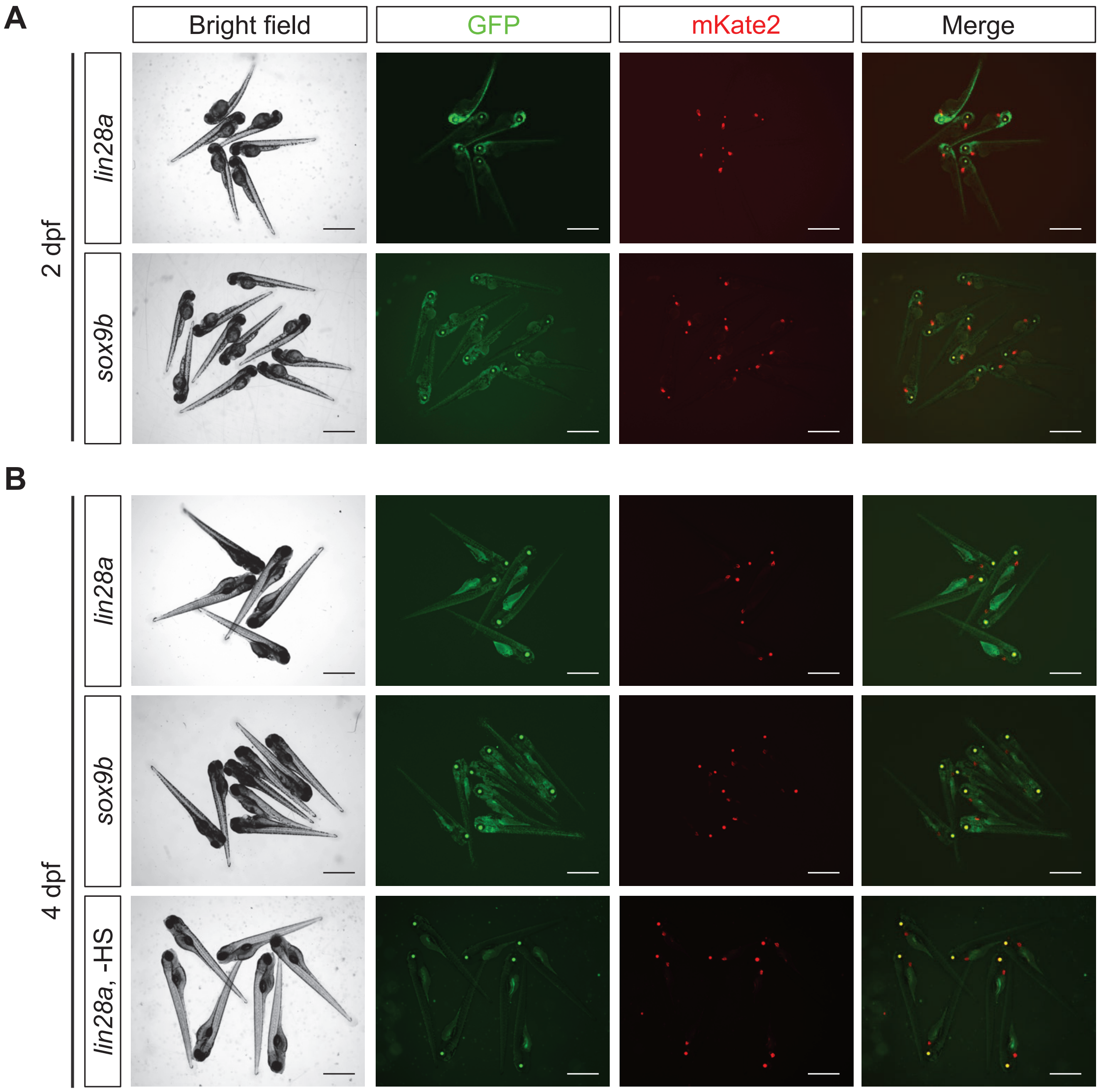

Supplementary Figure 14. Representative fluorescence images of 3Tg zebrafish embryos resulting from crosses between 2Tg zebrafish Tg(*hsp70l*:QFvpr-CK; 5×*QUAS*:sfGFP-dCas9vpr-AK) and transgenic zebrafish expressing different sgRNAs targeting *lin28a* or *sox9b.* Embryos were subjected to daily heat treatment at 2 and 3 dpf, and fluorescence images of EGFP were captured at 2 dpf (**A**) or 4 dpf (**B**). Heat shock-free (-HS) embryos derived from 3Tg *lin28a* transgenic embryos was included as a negative control (B). Scale bar, 1 mm.

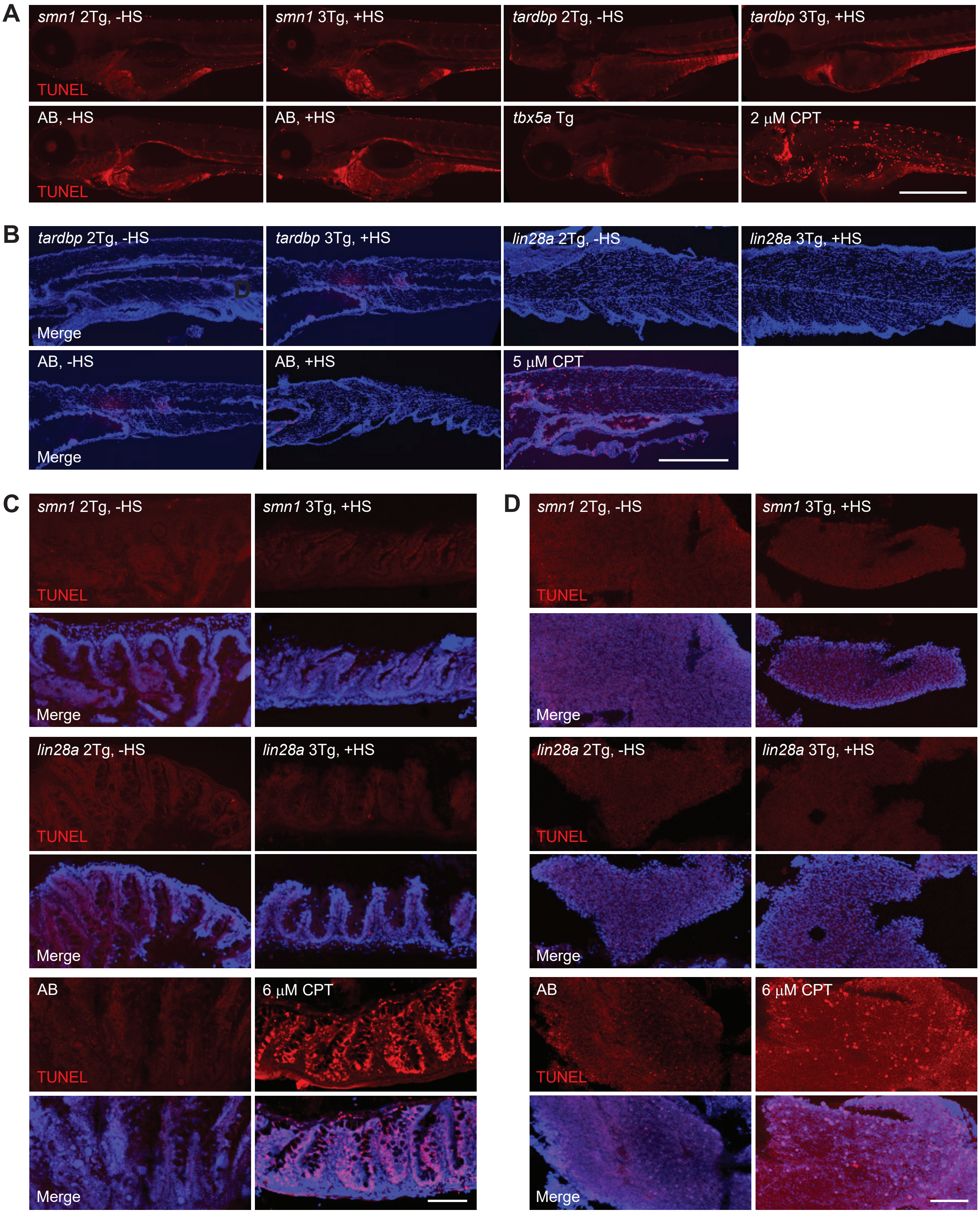

Supplementary Figure 15. Expression of CRISPR-Q effectors after heat shock does not cause cell death in larval, juvenile or adult zebrafish. (**A-D**) TUNEL staining of 4-day-old larvae (**A**), cryosections of 2-week-old juveniles (**B**), and cryosections of the gut (**C**) and liver (**D**) dissected from 3-month-old adults. Transgenic embryos, except for the *tbx5a* heart-specific CRISPR-Q_{KD} line, were subjected to daily heat treatment at 2 and 3 dpf (A). Transgenic juvenile and adult zebrafish underwent two heat shock treatments at 39 °C for 1 h each, with an 8-h interval between treatments (B-D). Scale bar, 500 µm (A, B); 100 µm (C, D). "Merge" denotes overlay of TUNEL and DAPI channels. CPT, camptothecin (positive control).

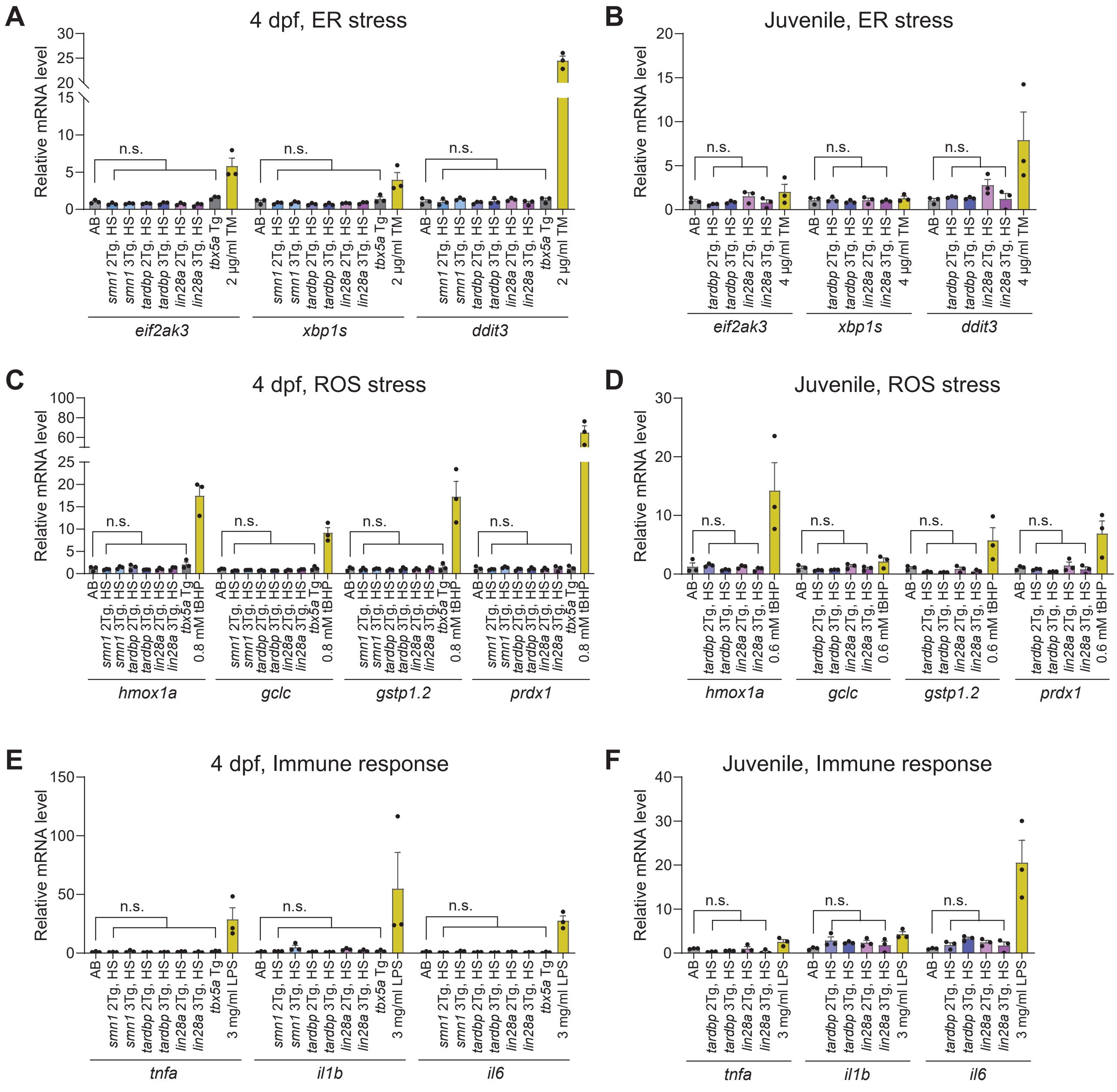

Supplementary Figure 16. Expression of CRISPR-Q effectors after heat shock does not induce ER stress, ROS stress, or immune responses in larval or juvenile zebrafish. (**A**-**F**) Bar plots show mRNA levels of the indicated genes in wild-type and transgenic larvae (**A**, **C**, **E**) or juvenile zebrafish (**B**, **D**, **F**), used to assess ER stress (A, B), ROS stress (C, D), and immune responses (E, F). Transgenic embryos, except for the *tbx5a* heart-specific CRISPR-Q_{KD} line, were subjected to daily heat treatment at 2 and 3 dpf (A , C, E). Transgenic juveniles underwent two heat shock treatments at 39 °C for 1 h each, with an 8-h interval between treatments (B, D, F). $n = 3$. Data are shown as mean ± s.e.m. Statistical analyses were performed using one-way ANOVA followed by Dunnett's multiple comparisons test. n.s., not significant. TM, tunicamycin; tBHP, tert-butyl hydroperoxide; LPS, lipopolysaccharide.

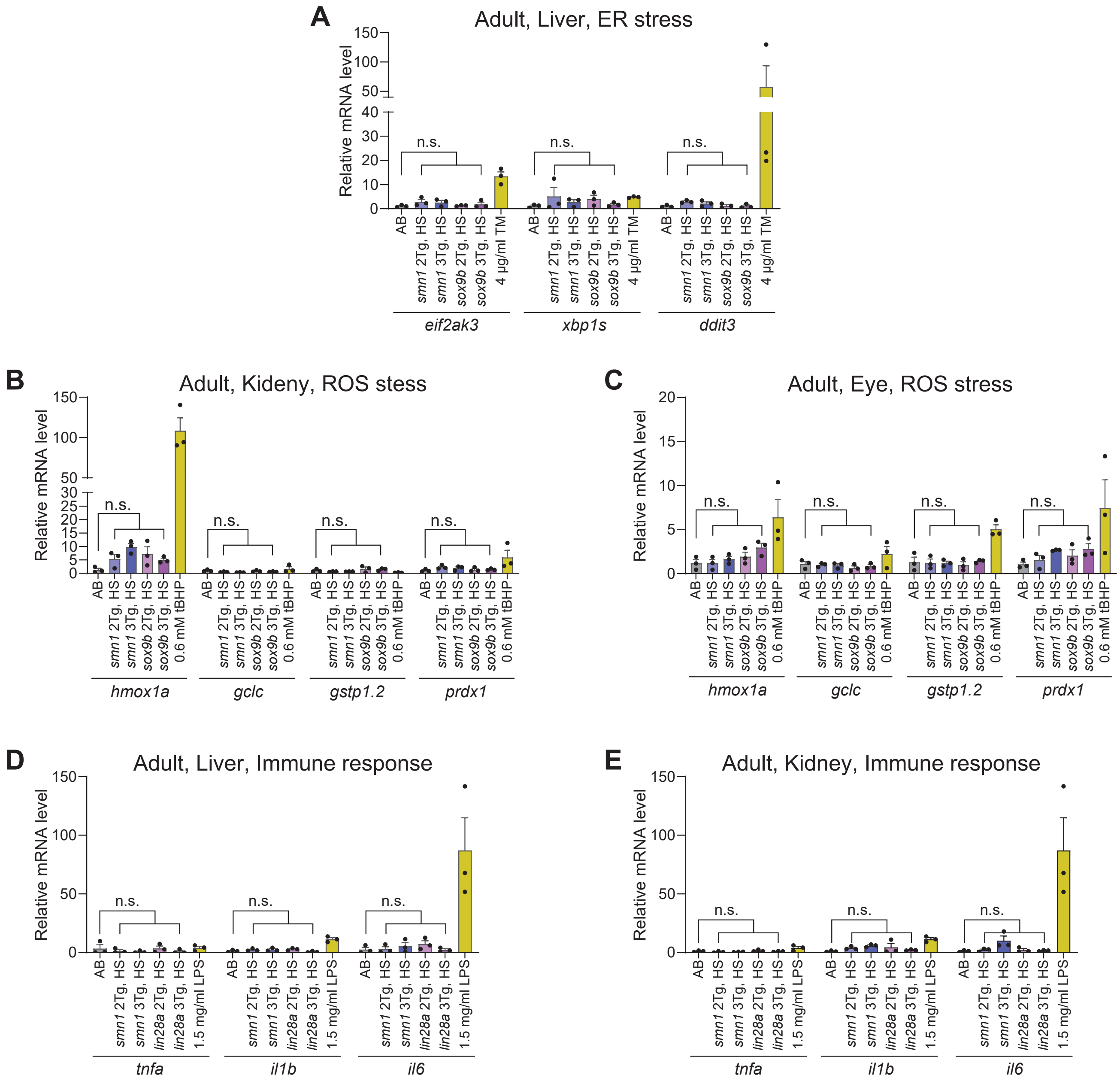

Supplementary Figure 17. Expression of CRISPR-Q effectors after heat shock does not induce ER stress, ROS stress, or immune responses in adult zebrafish. (**A-E**) Bar plots show mRNA levels of the indicated genes in different organs from wild-type and transgenic adult zebrafish, used to assess ER stress (**A**, **B**), ROS stress (**C**, **D**), and immune responses (**E**, **F**). Transgenic adult zebrafish underwent two heat shock treatments at 39 °C for 1 h, with an 8-h interval between treatments. n = 3. Data are shown as mean ± s.e.m. Statistical analyses were performed using one-way ANOVA followed by Dunnett's multiple comparisons test. n.s., not significant.

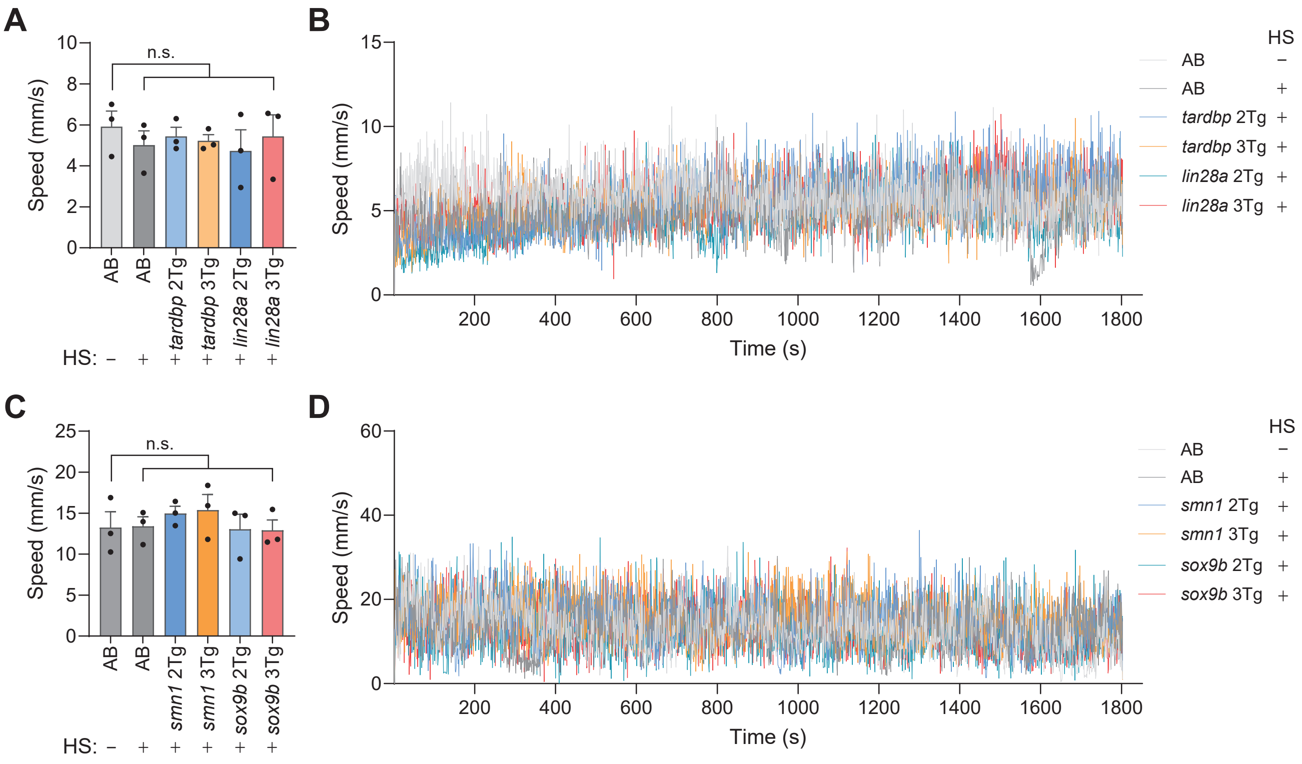

Supplementary Figure 18. Expression of CRISPR-Q effectors after heat shock does not affect basal locomotor activity in juvenile and adult zebrafish. (**A-D**) Plots show average swimming speed (**A**, **C**) and speed curves (**B**, **D**) recorded over 30 min under white light for juvenile (**A, B**) or adult zebrafishes (**C**, **D**) to assess baseline locomotor activity. $n = 3$. Data are shown as mean ± s.e.m. Statistical analyses were performed using one-way ANOVA followed by Dunnett's multiple comparisons test. n.s., not significant (A, C).

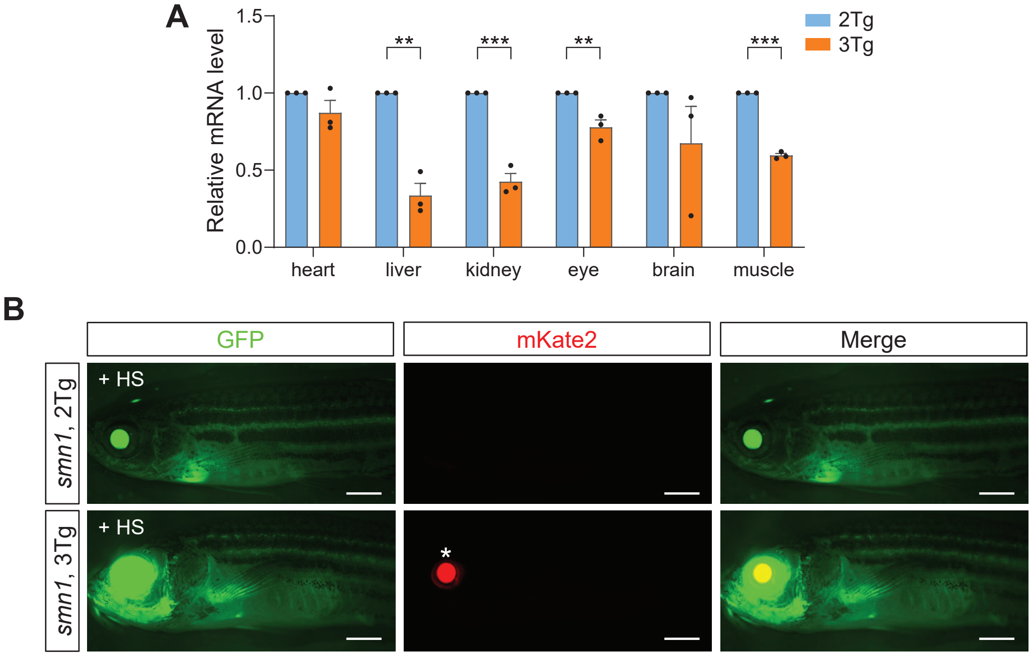

Supplementary Figure 19. CRISPR-Q_{KD} efficiently knocks down *smn1* in adult zebrafish. (**A**) qPCR analysis of *smn1* expression in the indicated organs. $n = 3$. Data are shown as mean ± s.e.m. P values were calculated using unpaired two-tailed t-tests. * $P < 0.05$; ** $P < 0.01$. (**B**) Representative images of 3-month-old *smn1* 2Tg and 3Tg adult zebrafish following two heat shock treatments at 39 °C for 1 h, with an 8-h interval between treatments. The asterisk indicates red fluorescence resulting from Venus signal bleed-through. Scale bar, 2 mm.

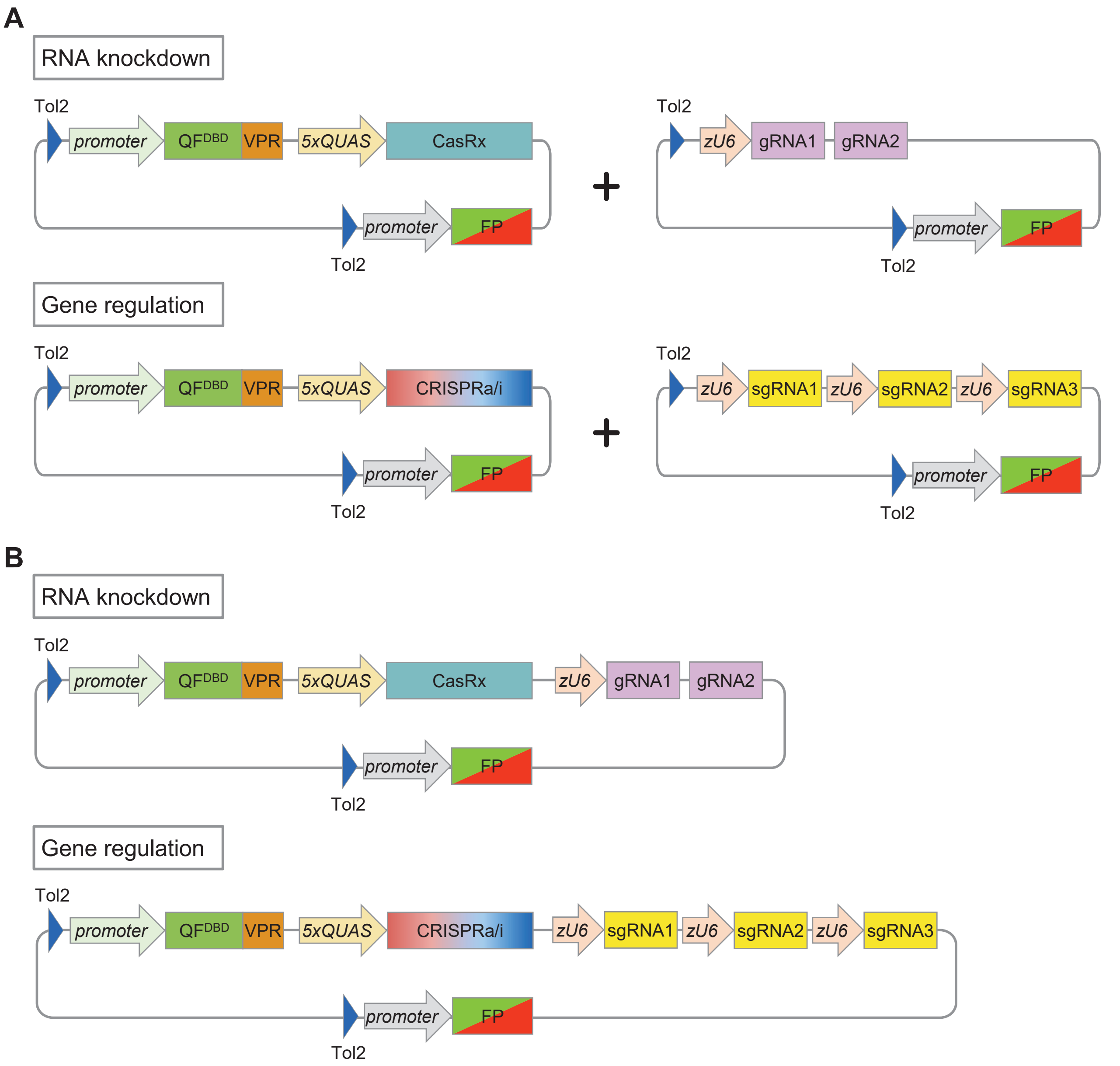

Supplementary Figure 20. Schematic design of plasmid constructs for the CRISPR-Q system. (**A**) A single-plasmid binary system containing QFvpr and 5×QUAS-Cas effector. The transgenic zebrafish line will be crossed with another transgenic zebrafish line expressing gRNAs for RNA knockdown or 3×sgRNAs for gene regulation. (**B**) A single-plasmid system for CRISPR-Q by combining QFvpr/QUAS, 5×QUAS-Cas effector, and gRNA/sgRNA in one plasmid construct. FP, fluorescence protein (FP) such as EGFP or mKate2, which is included as an expression marker.

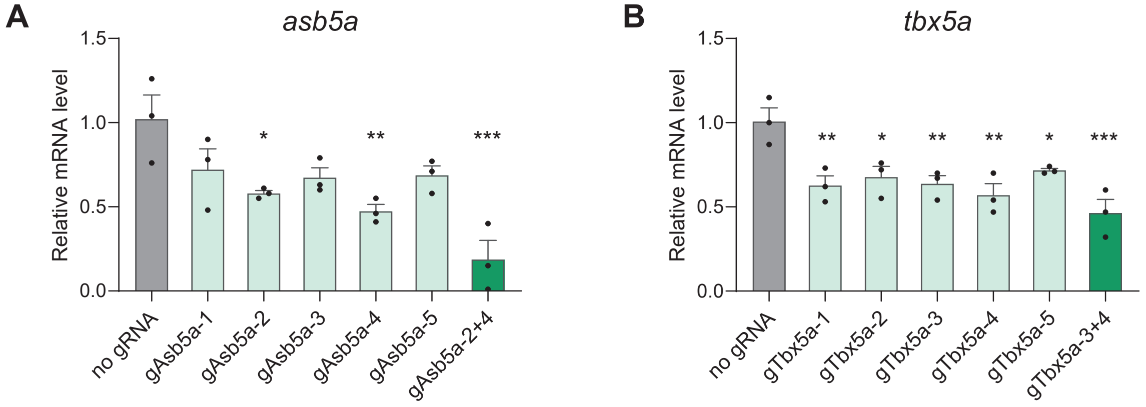

Supplementary Figure 21. Combination of gRNAs enhances knockdown efficiency by CasRx. (**A**, **B**) qPCR analysis of *asb5a* (**A**) or *tbx5a* (**B**) expression at 24 hpf in embryos injected with 300 pg CasRx mRNA and 300 pg indicated gRNA. $n = 3$. Data are presented as mean ± s.e.m. Statistical analyses were performed using one-way ANOVA followed by Dunnett's multiple comparisons test. * $P < 0.05$; ** $P < 0.01$; *** $P < 0.001$. gAsb5a, gRNA targeting *asb5a*; gTbx5a, gRNA targeting *tbx5a*.

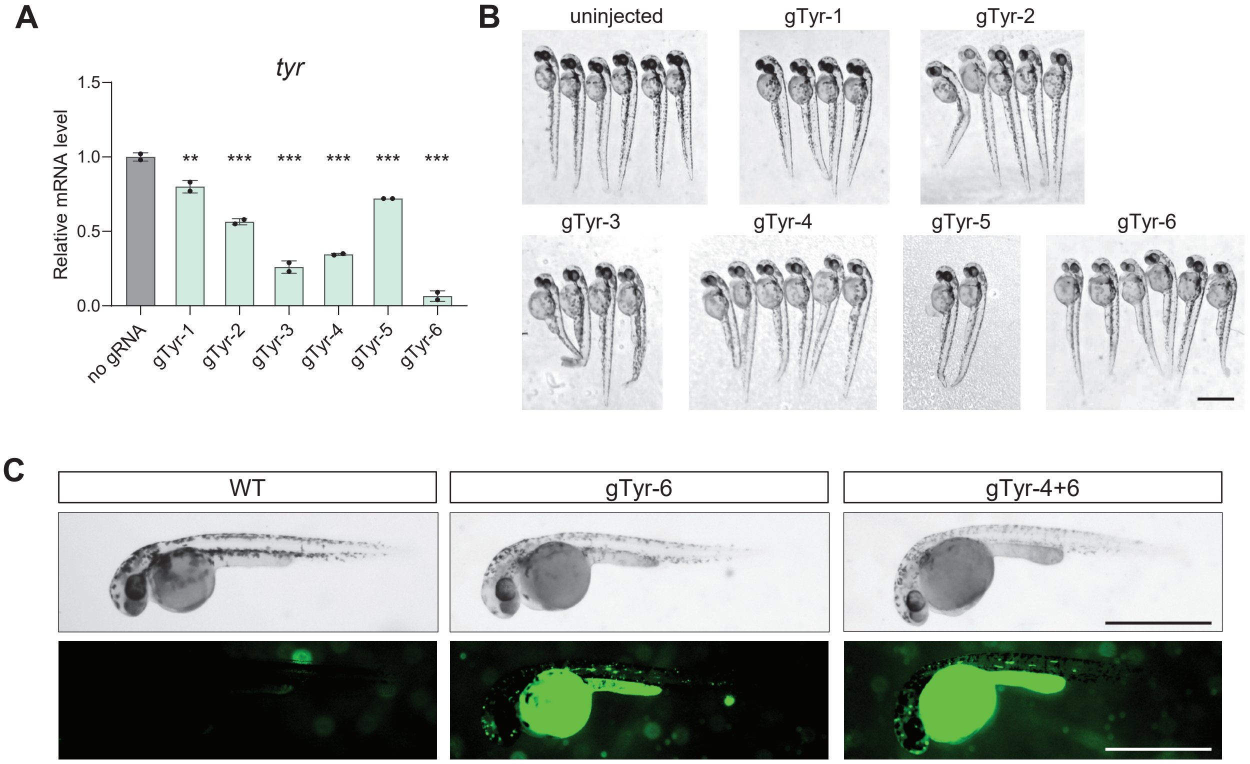

Supplementary Figure 22. Acute injection of CRISPR-Q_{KD} targeting *tyr* reduces pigmentation in embryos. (**A**) qPCR analysis of *tyr* expression at 24 hpf in embryos injected with 300 pg CasRx mRNA and 300 pg of the indicated gRNA ($n = 2$; mean ± s.d.). Statistical significance was assessed by one-way ANOVA followed by Tukey's multiple comparisons test. ** $P < 0.01$; *** $P < 0.001$. gTyr, gRNA targeting *tyr*. (**B**) Representative images of 2-dpf embryos injected with 300 pg CasRx mRNA and 300 pg of the indicated gRNA. (**C**) Representative images of 48-hpf uninjected AB wild-type embryos and embryos co-injected with 10 pg *ubb*:QFvpr-*5xQUAS*:CasRx-2AG-zU6:*tyr*-CG plasmid within indicated gRNAs and 20 pg Tol2 transposase mRNA. Scale bar, 1 mm (B, C).

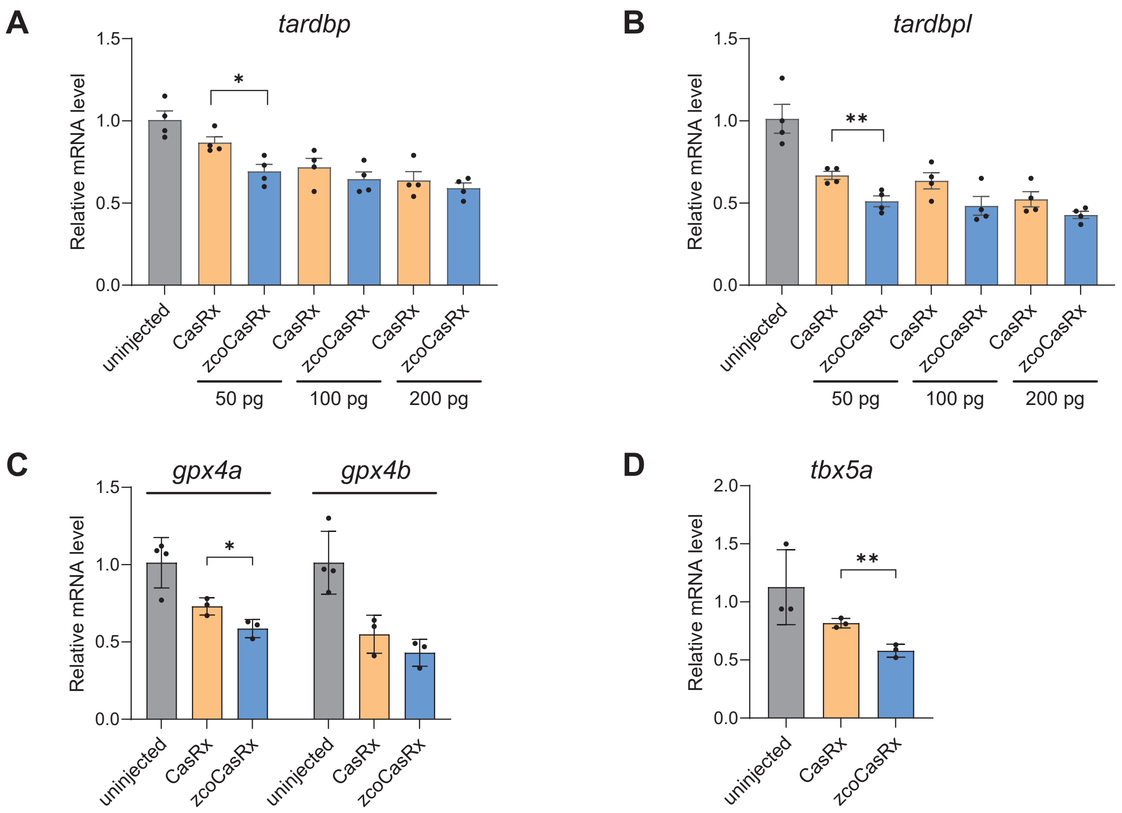

Supplementary Figure 23. Codon-optimized CasRx improves knockdown efficiency. (**A**, **B**) qPCR analysis of *tardbp* (**A**) or *tardbpl* (**B**) expression at 24 hpf in embryos injected with the indicated amount of CasRx or zcoCasRx (zebrafish codon-optimized CasRx) mRNA together with 100 pg gTardbp/tardbpl. (**C**, **D**) qPCR analysis of gpx4a and gpx4b (C) or tbx5a (D) expression at 24 hpf in embryos injected with 300 pg CasRx or zcoCasRx and 300 pg gGpx4a/gpx4b or gTbx5a-3. $n = 4$ (A, B). $n = 3$ (C, D). Data are presented as mean ± s.e.m. P values were calculated using unpaired two-tailed t-tests. * $P < 0.05$.

Supplementary Table S1. List of gRNAs used for transcript knockdown and activation

gRNAs for transcript knockdown

Gene	Transcript ID	Target sequence	spacer sequence
smn1	NM_131191	AGCTGTTGCATCATTCAAGAATG	CATTCTTGAATGATGCAACAGCT
gpx4a	ENSDART00000099056.5	CTGCTGTCGTTTTCTCTCTAGTG	CACTAGAGAGAAAACGACAGCAG
gpx4b	ENSDART00000113589.4	AGGAGCCTGGAAGTGAAGCGGAG	CTCCGCTTCACTTCCAGGCTCCT
tardbp	ENSDART00000058555.7	GATGATGGCACGGTTTTGCTTTC	GAAAGCAAAACCGTGCCATCATC
tardbpl	ENSDART00000139223.4	ACGGAGTGCTATATTCGTGTGGC	GCCACACGAATATAGCACTCCGT
urod	ENSDART00000014568.7	GCTTGCTGTGAGCTTACATTACA	TGTAATGTAAGCTCACAGCAAGC
tbx5a	ENSDART00000037691.6	1) TCGCGCTTGGAATATATTTATCA	TGATAAATATATTCCAAGCGCGA
		2) TTCGCCGATAACAAATGGTCTGT	ACAGACCATTTGTTATCGGCGAA
		3) CTTCGGCCATATCATCTTGAACT	AGTTCAAGATGATATGGCCGAAG
		4) GACTGCTCTTTTATTGCATCTTT	AAAGATGCAATAAAAGAGCAGTC
		5) TGAAGCAGCAAACATTGTGCTTT	AAAGCACAATGTTTGCTGCTTCA
asb5a	ENSDART00000138376.3	1) TCGCTCTTCTGCTTCAAACTTTT	AAAAGTTTGAAGCAGAAGAGCGA
		2) GGAGTGACTCCATTGTTTCGTGC	GCACGAAACAATGGAGTCACTCC
		3) ATGCGGCATCTCAGAAAGATTGT	ACAATCTTTCTGAGATGCCGCAT
		4) AGGGTTTCCTATTGATTTATGAA	TTCATAAATCAATAGGAAACCCT
		5) TGTTGTGGGTTAATAATACTATA	TATAGTATTATTAACCCACAACA
myh6	ENSDART00000126983.3	TCACGATGGGTGATGCTTTAATGGCAGAGT	ACTCTGCCATTAAAGCATCACCCATCGTGA
		TGCGGTGCTGTTCAATCTAAAGG	CCTTTAGATTGAACAGCACCGCA
myl7	ENSDART00000040013.3	AAACCATCCTTGCTGCTTTTAAATTGTTCG	CGAACAATTTAAAAGCAGCAAGGATGGTTT
		GTGGCTGGAAATATTGATTATAA	TTATAATCAATATTTCCAGCCAC
mGL		ACCTTAGGCTACGGCGTGGCCTG	CAGGCCACGCCGTAGCCTAAGGT
		GCCATCAGTCCAAACTGAGCAAA	TTTGCTCAGTTTGGACTGATGGC
		GATTACACATGACATGGACGAGC	GCTCGTCCATGTCATGTGTAATC
tyr	ENSDART00000122238.3	1) TCTGTGCGCAGAAATATATTCCA	TGGAATATATTTCTGCGCACAGA
		2) GATGGCCTTTAGTGTTTTACAAC	GTTGTAAAACACTAAAGGCCATC
		3) CGTACGCGCAGATGAACAACGGC	GCCGTTGTTCATCTGCGCGTACG
		4) GTGTGGGCTGATATTGATTTTGC	GCAAAATCAATATCAGCCCACAC
		5) ACCCCATTTTCATCATACATCAT	ATGATGTATGATGAAAATGGGGT
		6) AAGTGCACTACATGTGTTACAGT	ACTGTAACACATGTAGTGCACTT

gRNAs for transcription activation

Gene	gRNA No.	Target sequence	Strand
lin28ab	1	AGAGCAGTGAGTCAGTCTGC	antisense
	2	GCGGCTTTAATTGTGTTTGT	sense
	3	ATACCGCGCGTTATTTAAAA	antisense
myca	1	AAGCAACGCCCCCTGACGTA	antisense
	2	ATTCGCAAACTACCGTGTAT	sense
	3	AAGGAAAACAGTACGCAGAC	antisense
sox9b	1	GACACTTATATAGTGCAGAG	antisense
	2	ACTAGCAAGTGCAGTAAAGT	antisense
	3	AAGTCAAGTCAGACCAAGAG	antisense

Supplementary Table S2. List of primers for qPCR

Primers for CRISPR-Q_{KD} or CRISPR-Qa efficiency		
Gene	Forward primers	Reverse primers
actin	GATGATGAAATTGCCGCACTG	ACCAACCATGACACCCTGATGT
smn1	GACATTTGGGATGATACAGCTTTG	TTCCCTGGGTTGTCGTTTT
urod	CACGGGCAGGGAAAGATT	CAGGATGTCGGAGAAGATGATG
tardbp	GGTCGTTCTTGGTCAGAGTTT	CTTTGTTAGCACCGCCAGCAGC
tardbpl	GGCTCCGTTTGTTGCCTTAG	CCATCGGCTCCTCGTTTT
gpx4a	AGTTCAGGGACATTGAGGATTT	GACATAGTCTGCAACACTAGAGAG
gpx4b	CGTACGCTGAGAAGGGTTTAC	CTCCTTAATCTCCGCTTCACTTC
myca	GCGCAGGAATGAACTCAAAC	GCACTCTGTCGCCTTCTTTA
lin28a	GTACAAAGACAGAGGAGGAAGAAG	GGTGGGTCATGGACAGAAAT
sox9b	CTGAAGTCCGCCTGAATCAC	TGGACACCCTCAGGTTAACG
tyr	CGAGTCTGTGCGCAGAAATA	GCGTACGTTCCTGTTACGATC
asb5a	AAGAGTTCCTATGTGCCCGC	TTCATAAATCAATAGGAAACCCT
tbx5a	TGAAGATTCCTGCTTCCTTGA	CTAGCGGTTGATAGCGAACT
sox9a	ATTCGACGTCAATGAGTTTGACC	TCGGTTTTAATATGCGTCGTTCG
col2a1a	TCGTGCTGTCCGCGTTTAG	TGGTAGTGCTCGCATGTTCG
col2a1b	TTCTGGTGCTCGTGGTAATGAC	TCAGAACCAGGGTTACCAGAG
col11a1b	CAAAGGAAACCCAGGTCCTATCG	ATCTCCATCATCTCCCTTGTCTC
col11a2	ACTCGCTGTACTAAACAATGTGG	ATCATACAGAGAAGGCGGATTTG
cspg4	TCAAACTCATCAATGACTCGGAC	TGTGTGATATTGAGCACGTCTTG
lrrc74a	GCCATCGAGGACATCAACATTTG	GAGCTTTCTCTGATCGAGGTAATC
mfsd11	CCTTCGCATACTCTGGTCTGG	ACACGCCACCTCCAACTATTTC
ppp2r2bb	TCTCCCGGTACCTGCCTTAC	CACCACTCGTCCACCTTTGTC
tshr	TCAGAGACACCTATGATCACCAG	CCATGTTCTCATCTGCATGTCTG
myh6	ATTCAAGCTAACCCTGCGCT	GCTCGTCCCGAAATGAATGC
myl7	GGAGGCTTTTGGCTGCATAG	ATGGGCCCTTTTCCTTCTGT

Primers for gRNA quantification	
Name	Sequence
gRx-F	GAACCCCTACCAACTGGTCGG
gRx-smn1-R	AAAAGCTGTTGCATCATTCAAG
gRx-tardbp-R	AAGATGATGGCACGGTTTTGC
gRx-tardbpl-R	AAAACGGAGTGCTATATTCGTG
Cas9-sgRNA-R	GACTCGGTGCCACTTTTTCAAG
sox9b-sgRNA-F1	GACACTTATATAGTGCAGAGGTTTAAGAG

Primers for copy number analysis		
Name	Forward primers	Reverse primers
gapdh	AACCGTGTATGTGACCTGATGG	TCAACCAGATGGGAGAATGGTC
actb1	ATGTTCGAGACCTTCAACACCC	TCACCAGAGTCCATCACAATACC
QF-DBD	CGCTTGCTTTCCCTGTGTTTC	GCCAGCTCCAGAGTTCTAATGTATC
CasRx	GGTCTACACCATGATGGACTTTG	TCGCTCAGGGACTTCTCATTATC
dCas9	CCCAAGAGGAACAGCGATAAGC	GCCACCACCAGCACAGAATAG
AV	ATGTTGTCAGACCTGGTAACTCC	CTAGCCTCTATTAGCCTCCTACTTG

Primers for assessment of stress and immune responses		
Gene	Forward primers	Reverse primers
eif2ak3	acgtacatcgtccgcaagttc	gcagggacgcatatctgtgac
xbp1s	agcaagtaccttcctccgcac	tgcgggtttggtgtagatgtg
ddit3	tgcatgacagcaattaaactaagcg	tggtcaatgtagttttggacgttttg
hmox1a	gcaggacttggagcacttcttc	tcgggactgctcttgccaatc
gclc	tgccaagccggatcacatctac	cgctcagagccatcactatgg
gstp1.2	acctgtgtctttgggcagttg	ttcaacgccgtcgttcatcac
prdx1	gcgagacttgagcacgacctat	tgctttggctgtgaaatccgg
tnfa	acaagatggaagtgtgctgagac	ttcaagccacctgaagaaaaggc
il1b	ggacttcgcagcacaaaatgaag	ttcacttcacgctcttggatgac
il6	tcctctcctcaaaccttcagacc	tgctgtgtttgatgtcgttcacc
